# Microhydrated 3‐Methyl‐3‐oxetanemethanol: Evolution of the Hydrogen‐Bonding Network from Chains to Cubes

**DOI:** 10.1002/anie.202210819

**Published:** 2022-11-09

**Authors:** Wenhao Sun, Melanie Schnell

**Affiliations:** ^1^ Deutsches Elektronen-Synchrotron DESY Notkestr. 85 22607 Hamburg Germany; ^2^ Institut für Physikalische Chemie Christian-Albrechts-Universität zu Kiel Max-Eyth-Str. 1 24118 Kiel Germany

**Keywords:** Hydrogen Bonding, Microsolvation, Quantum Chemistry, Rotational Spectroscopy, Water Clusters

## Abstract

Broadband rotational spectroscopy is used to investigate the geometries of 3‐methyl‐3‐oxetanemethanol and its complexes with up to six water molecules, which are produced in supersonic jets. The main low‐energy isomers of these clusters are unambiguously identified in the spectra with the support of quantum‐chemical calculations. The conformation of the 3‐methyl‐3‐oxetanemethanol geometry is found to be influenced by the microsolvation effects. The hydrogen‐bond arrangements in the hydrate complexes, which are governed by the water‐water and water‐solute interactions, exhibit characteristic configurations with increasing number of water molecules and resemble the main isomers of the corresponding pure water clusters. Evolution of the hydrogen‐bonding structures from one‐dimensional chains to two‐dimensional rings and further to multicyclic three‐dimensional networks is observed, which provides information about the build‐up process.

## Graphical abstract

The gas‐phase MOM‐(H_2_O)_*n*_ (*n*=0–6) clusters are studied using rotational spectroscopy. The growth of the H‐bonding networks in these complexes exhibits characteristic transitions from one‐dimensional chains, to two‐dimensional rings, and further to three‐dimensional arrangements that leads to a “cube”‐like network resembling the pure water octamer.

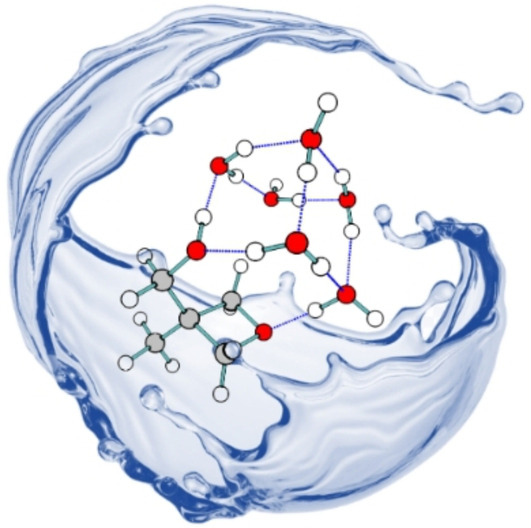

## Introduction

In recent years, oxetane derivatives, especially the 3,3‐disubstituted oxetanes, have received growing attention in drug discovery and medicinal chemistry.[^1^, ^2^] As an alternative for the carbonyl group and the *gem*‐dimethyl group, the oxetane ring can introduce a stable motif into medicinal compounds, for instance, the 1,25‐dihydroxyvitamin D_3_ analogues,^[3]^ and often provides better physicochemical properties, such as binding and solubility effects. In this regard, the properties of the oxetane‐containing compounds in different chemical environments, including the flexibility of the four‐membered ring, the reactivity of the ether moiety, and the cooperativity of the hydrogen bond (H‐bond), are of particular concern.[^4^, ^5^] Rotational spectroscopy, which can characterize molecules in the gas phase with high structural sensitivity,^[6]^ offers a robust approach to study these molecules in the “interaction‐free” environment of the gas phase. During solvation processes, their physical and chemical properties, such as conformational preferences, may deviate from those in vacuum or in the solution phase.^[7]^ Microsolvated clusters can be produced in the gas phase using supersonic jets. Investigation of such microsolvated clusters provides valuable insight into solvation at the molecular level, by pinpointing the initial steps of the solvation process and explicitly establishing the subtle non‐covalent interactions between the solute and solvent molecules.[^8^, ^9^]

As water and water solutions are ubiquitous in nature and play a fundamental role in life sciences, it is imperative to explore solvation with water as the solvent. In the last decades, great efforts have been made to elucidate the structures of microsolvated complexes with water aggregates as well as pure water clusters and to gain molecular details of them, such as the cooperativity in H‐bonding and the rearrangement dynamics of their H‐bond networks.[^9^, ^10^, ^11^, ^12^] On the one hand, it is a challenge to experimentally prepare and characterize size‐specific solvent‐solute complexes in the gas phase, including pure water clusters. On the other hand, it is also demanding to precisely model and predict such molecular systems with quantum‐chemistry methods. To date, pure water clusters ranging from the dimer to the decamer have been extensively studied using THz vibration‐rotation‐tunneling (VRT) spectroscopy[^12^, ^13^, ^14^, ^15^, ^16^, ^17^] and broadband Fourier transform microwave (FTMW) spectroscopy,[^10^, ^18^, ^19^] in combination with a supersonic expansion source. These water clusters have clear preferences regarding the H‐bonding architecture. While the water molecules in a water dimer are simply bound by a linear H‐bond,^[13]^ two‐dimensional (2D) quasi‐planar H‐bonded cycles are preferred in the water trimer, tetramer, and pentamer.^[16]^ Starting from the water hexamer, multicyclic three‐dimensional (3D) H‐bonded networks become more favored. Larger clusters (up to ten water molecules) expand on the basis of the hexamer structures,[^18^, ^19^] known as the prism, cage, and book isomers.^[10]^

When the solute molecules are introduced into the system, not only are the water‐water interactions crucial but also the interplay between the solute molecules and the pure water H‐bonding networks. By means of resonant two‐photon ionization, ultraviolet hole‐burning, and resonant ion‐dip infrared spectroscopy, molecular systems including aromatic species, such as benzene (Bz)‐water[^9^, ^20^, ^21^, ^22^] and phenol‐water,[^23^, ^24^, ^25^] were investigated with up to eight water molecules. The experimentally identified H‐bonding networks in these microsolvated complexes resemble the pure water clusters of the similar size, as they are either barely affected with the presence of the aromatic ring or mildly perturbed via OH⋅⋅⋅π bonds. The re‐arrangements from 2D rings to 3D networks occur for benzene‐(H_2_O)_6_ and phenol‐(H_2_O)_5_, while the favored H‐bonding networks are different from the pure water clusters.[^20^, ^24^] Moreover, numerous microsolvated organic molecules have been explored with microwave spectroscopy. However, the detected weakly bound complexes are mostly limited to up to three water molecules.[^26^, ^27^, ^28^, ^29^, ^30^, ^31^] The largest one reported so far is the β‐propiolactone (BPL)‐(H_2_O)_5_ cluster,^[32]^ in which the five water molecules form a 2D H‐bonded ring, resembling the pure water pentamer. The ring is parallelly bound to the BPL plane through four H‐bonds. Recently, with the developments of instrumentation and quantum‐chemical approaches, larger clusters with up to six and seven water molecules are experimentally discovered, such as glycolaldehyde‐(H_2_O)_6_ ^[33]^ and fenchone‐(H_2_O)_7_,^[34]^ where the H‐bonding architectures undergo further structural evolution from 2D arrangements to 3D networks.

In the present study, 3‐methyl‐3‐oxetanemethanol (MOM) is extensively explored in the gas phase, including its monomer and complexes with water molecules, using broadband chirped‐pulse FTMW spectroscopy. The heavy atom backbone structures of the MOM conformers are precisely determined via the analysis of the ^13^C and ^18^O singly substituted isotopologues in their natural abundances and with the deuteration of the hydroxyl group. Furthermore, the microsolvated clusters with up to six water molecules are unambiguously identified with the support of quantum‐chemical calculations. The growth of the H‐bonding networks in these clusters is rationalized, which exhibit characteristic rearrangements from linear chains to quasi‐planar cycles and to 3D networks.

## Results and Discussion

**Figure 1:**
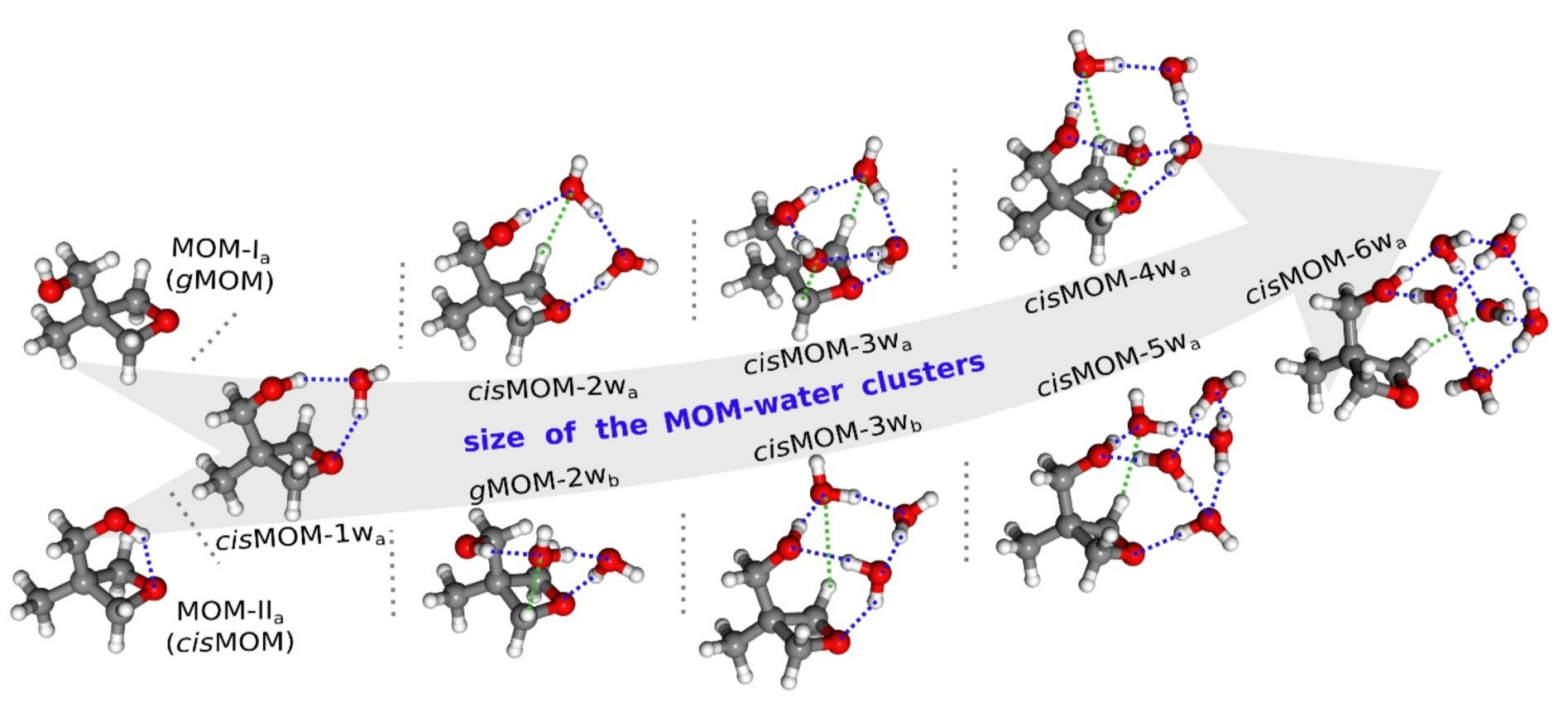
The experimentally observed geometries corresponding to the 3‐methyl‐3‐oxetanemethanol (MOM) monomer and its complexes with up to six water molecules. The blue and green dashed lines indicate the primary hydrogen bonds (O−H⋅⋅⋅O) and the secondary hydrogen bonds (C−H⋅⋅⋅O), respectively.

The rotational spectra were measured using broadband FTMW spectroscopy in the frequency ranges of 18–26 GHz and 2–8 GHz. By combining microwave spectroscopy and theoretical calculations, the geometry of the MOM monomer and its complexes with up to six water molecules are unambiguously assigned based on the rotational constants, the electric dipole moment components, and their relative energies, which are provided in the Supporting Information. The experimentally observed geometries are summarized in Figure 1. In the following sections, the structural differences and evolution of clusters with increasing number of water molecules will be discussed in detail.

**Figure 2:**
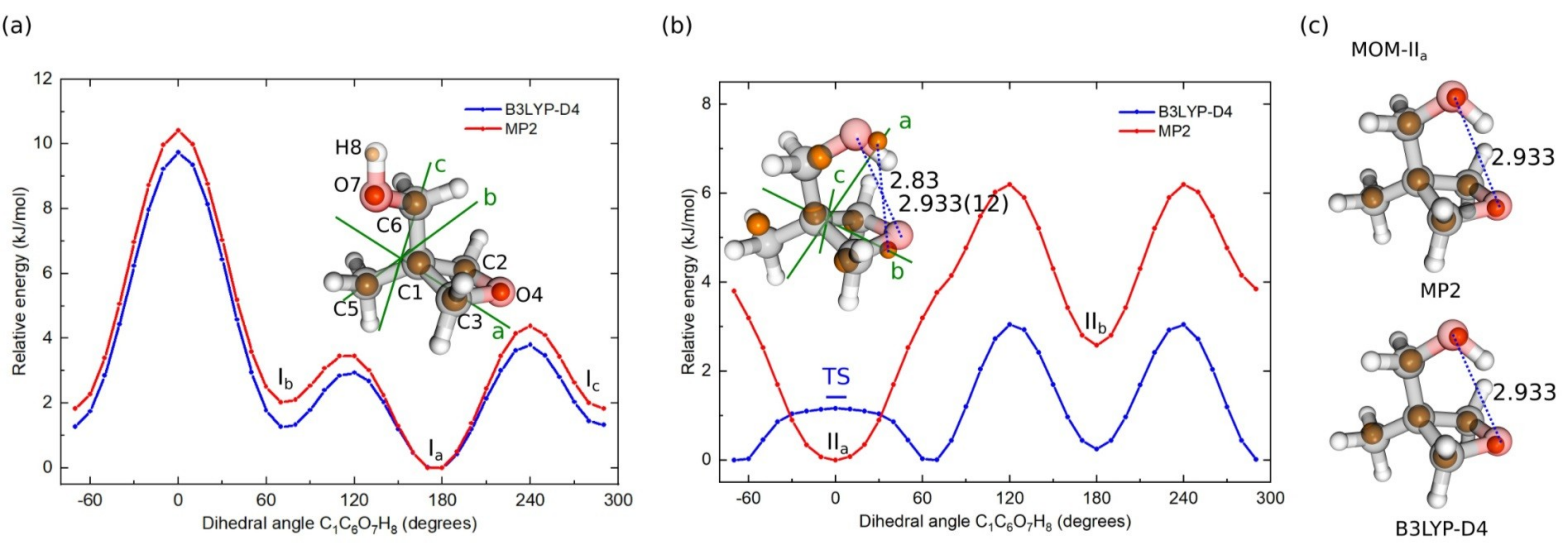
Potential energy curves (PECs) obtained by rotating the hydroxyl group (−O_7_H_8_) about the C_6_–O_7_ bond in the *gauche*‐ (a) and *cis*‐O_4_C_1_C_6_O_7_ (b) conformations of the MOM monomer at the B3LYP‐D4/def2‐QZVP and MP2/cc‐pVQZ levels of theory. The relative energy of MOM‐I_a_ and MOM‐II_a_ is set to 0 kJ mol^−1^, respectively. The blue bar at 0° in panel (b) indicates the relative energy of the transition state (TS) at the B3LYP‐D4/def2‐QZVP level with zero‐point energy (ZPE) corrections with respect to the local minimum geometry at 67°. The experimentally determined *r_s_* atomic positions (orange spheres) in MOM‐I_a_ (a) and MOM‐II_a_ (b) are displayed together with the corresponding equilibrium *r_e_* structures in their principal inertial axes systems, obtained with the MP2 method. Panel (c) displays the comparisons of the experimental *r_s_* coordinates (orange spheres) with the geometries of MOM‐II_a_, calculated from constraint optimizations at the MP2/cc‐pVQZ (top) and the B3LYP‐D4/def2‐QZVP (bottom) levels of theory, where the O_4_⋅⋅⋅O_7_ distance is fixed to 2.933 Å.

**Figure 3:**
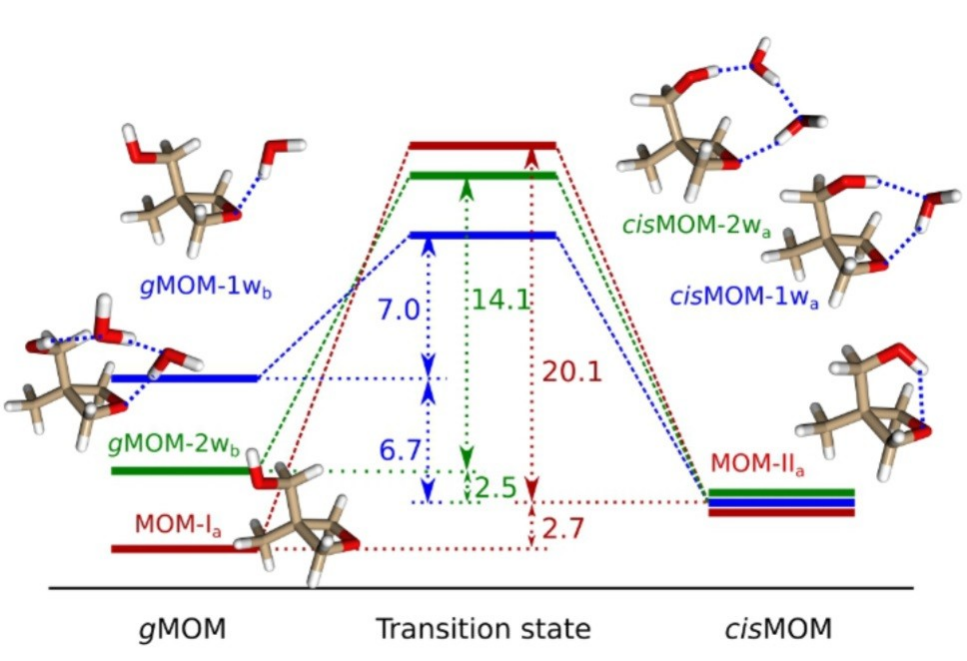
Interconversion pathways between *g*MOM and *cis*MOM for the MOM monomer (red), monohydrate (blue), and dihydrate (green), calculated at the B3LYP‐D4/def2‐QZVP level of theory with ZPE corrections. The values (in kJ mol^−1^) represent the conversion barriers from the higher energetic isomer to the lower energetic form and the relative energies between the two isomers. The relative energies of the *cis*MOM isomeric forms are set to 0 kJ mol^−1^. The blue dashed lines in the geometries indicate the hydrogen bonds.

For the MOM monomer, five conformers adopting two heavy‐atom backbone arrangements are predicted at both B3LYP‐D4/def2‐QZVP[^35^, ^36^, ^37^, ^38^, ^39^] and MP2/cc‐pVQZ[^40^, ^41^, ^42^] levels of theory, as shown in Figure 2, and two conformers are observed in the spectrum. Three of the predicted conformers (MOM‐I_a_, MOM‐I_b_, and MOM‐I_c_) adopt a *gauche* configuration of O_4_C_1_C_6_O_7_ with the hydroxyl group (OH) pointing in different directions, while the other two (MOM‐II_a_ and MOM‐II_b_) adopt a *cis* configuration. The interconversion barrier between the *gauche* and *cis* conformation is about 20 kJ mol^−1^ (Figure 3). Among the *gauche* conformers, MOM‐I_a_, in which the hydroxyl group takes the *trans* arrangement, is the energetically most stable form. MOM‐I_b_ and MOM‐I_c_ are higher in energy and are readily relaxed to MOM‐I_a_ in the supersonic expansion, as the interconversion barriers (MOM‐I_b_–>MOM‐I_a_ and MOM‐I_c_–>MOM‐I_a_) are below 4.2 kJ mol^−1^, which was determined as an approximate energy threshold of the conformer/isomer relaxation in a supersonic expansion when using Ne as carrier gas.^[43]^ The rotational spectrum arising from the parent species of MOM‐I_a_ and all its singly substituted rare isotopologues of the heavy atoms, namely five ^13^C and two ^18^O, are observed in natural abundances and agree with the quantum‐chemical predictions, as given in Tables S1–S3 in the Supporting Information. The *gauche*‐*trans* conformation is further confirmed with the deuteration of the OH group, denoted as MOM‐OD, which is obtained by proton exchange with heavy water (D_2_O). The substitution (*r_s_*) structure calculated using the Kraitchman's equations^[44]^ is represented in Figure 2a together with the optimized equilibrium geometry (*r_e_*) at the MP2/cc‐pVQZ level of theory to show the good agreement. The *r_e_* geometry predicted with the B3LYP‐D4/def2‐QZVP method is also consistent with the experimental *r_s_* structure.

When it comes to the two *cis‐*O_4_C_1_C_6_O_7_ conformers (MOM‐II_a_ and MOM‐II_b_), the two computational methods (B3LYP‐D4 and MP2) agree on the arrangement of the OH group in the higher energetic form (MOM‐II_b_) and predict a similar barrier for the conversion (about 2 kJ mol^−1^) from MOM‐II_b_ to MOM‐II_a_, but they disagree on the OH orientation in MOM‐II_a_, as indicated in Figure 2b. The MP2 method predicts that MOM‐II_a_ has a *C_s_* molecular symmetry, where the OH group is situated in the *ab*‐plane. However, at the B3LYP‐D4 level, the global minimum exhibits a 67° rotation of the OH group out of the *ab‐*plane, and the *C_s_* configuration becomes a transition state that is located 1.5 kJ mol^−1^ higher (including zero‐point energy (ZPE) corrections). This is presumably due to different descriptions of the attractive forces in the intramolecular H‐bond O_7_‐H_8_⋅⋅⋅O_4_ and the ring strain of the oxetane motif. In order to further evaluate this disagreement, the potential energy curve (PEC) is re‐computed with single‐point energy calculations at the CCSD(T)/cc‐pVTZ level of theory using the optimized geometries of the B3LYP‐D4 and MP2 PECs, respectively, and with relaxed potential energy scans using various other methods, including the B2PLYP‐D4/def2‐QZVP, PWPB95‐D4/def2‐TZVP, M06‐2X−D3/def2‐QZVP, and PW6B95‐D4/def2‐QZVP levels of theory. The results are summarized in Figure S7 in the Supporting Information. All of them agree with the MP2 PEC, where the geometry with *C_s_* symmetry is the minimum configuration. Furthermore, only *a‐* and *b‐*type transitions are observed in the spectra, which agrees better with the electric dipole moment components predicted by the MP2 method (B3LYP‐D4: |*μ_a_* |, |*μ_b_* |, |*μ_c_* |=0.2, 2.1, 1.1 Debye; MP2: |*μ_a_* |, |*μ_b_* |, |*μ_c_* |=2.9, 0.8, 0.0 Debye). The obtained rotational constants also match better with the MP2 prediction, as provided in Tables S2 and S3 in the Supporting Information. Note that this difference in the dipole‐moment components most likely arises from the somewhat different arrangement of the O_7_‐H_8_ bond, as described above. In addition, the ^13^C isotopologues at C_2_ and C_3_ are found to be equivalent, with the spectra twice as intense as that of other ^13^C isotopologues (C_1_, C_5_, and C_6_), which favorably supports the MP2 prediction. The second moment^[45]^ along the inertial *c*‐axis (*P_cc_*), which measures the extensions of the masses along the *c*‐axis, is 39.76 amu Å^2^ for the normal species and remains unchanged with the singly substituted ^13^C isotopologues at C_1_, C_5_, and C_6_, the ^18^O isotopologues at O_4_ and O_7_, and the OH deuterated species (MOM‐OD). This also indicates that these atoms are situated in the same plane (*ab*‐plane) and that MOM‐II_a_ has *C_s_* symmetry about the *ab*‐plane. Note that, although the assignment of MOM‐OD helps to locate the H_8_ in the *ab*‐plane, the position of the proton H_8_ cannot be accurately determined via the structural evaluations, as the H‐bond becomes stronger with the deuteration, making the O_4_⋅⋅⋅O_7_ distance shorter in MOM‐OD.^[46]^

Although the MP2 method predicts the correct orientation of the OH group in MOM‐II_a_, it overestimates the intramolecular H‐bond. It predicts an O_4_⋅⋅⋅O_7_ distance of 2.83 Å, which is about 0.1 Å shorter than the experimentally determined value. The theoretical structure cannot be well aligned with the experimentally determined *r_s_* structure in the principal inertial axes system, as shown in Figure 2b. By fixing the O_4_⋅⋅⋅O_7_ distance to the experimental value (2.933 Å) and re‐optimizing the geometry, the alignment gets substantially improved (see Figure 2c). Due to this issue, the MP2 method fails to compute the correct relative energy of the conformers: it predicts MOM‐II_a_ to be about 0.4 kJ mol^−1^ lower in energy than MOM‐I_a_, whereas based on the spectral strengths from our experiment and the calculated electric dipole moment components (*μ_a_* and *μ_b_*) of the observed conformations, MOM‐I_a_ is about three times more abundant than MOM‐II_a_ in the jet expansion, suggesting that MOM‐I_a_ is about 1–2 kJ mol^−1^ more stable than MOM‐II_a_.

Next, with the presence of water vapor in the gas line, a variety of water complexes with MOM were formed in the jet expansion. The formed water clusters around MOM will affect its structure. In order to search for the geometries of the MOM hydrates, the GFN‐xTB method is used in the CREST routine to obtain preliminary geometry predictions.^[47]^ Although the B3LYP‐D4/def2‐QZVP level of theory has problems to predict the correct conformation of the MOM monomer, we observed that it offers a reliable performance with a reasonable computational cost for optimizing the geometries of the MOM hydrates and is, thus, used to further optimize the preliminary geometries from CREST. The main MOM hydrate isomers are confirmed with frequency calculations at the same B3LYP‐D4/def2‐QZVP level and with structural optimizations at the MP2/cc‐pVQZ level of theory, which are provided in the Supporting Information. In the following discussions, the relative energies of the water complexes are from the B3LYP‐D4/def2‐QZVP level of theory with ZPE corrections, unless otherwise mentioned. Although MOM‐I_a_ and MOM‐II_a_ are experimentally observed monomeric conformers, which adopt *trans‐* and *cis*‐C_1_C_6_O_7_H_8_ arrangement, respectively, the ‐O_7_H_8_ group may rotate and take different arrangements due to the water‐solute interactions in the hydrate complexes. Therefore, in what follows *g*MOM and *cis*MOM are used to indicate the heavy atom backbone structures, which adopt *gauche*‐ and *cis*‐O_4_C_1_C_6_O_7_ conformations, regardless of the orientation of the ‐O_7_H_8_ group.

According to the calculations for the MOM monohydrate, there are two isomers predicted within a relative energy window of 10 kJ mol^−1^, which are formed with *cis*MOM and *g*MOM, respectively, denoted as *cis*MOM‐1w_a_ and *g*MOM‐1w_b_. Despite that *g*MOM is more favored for the isolated monomers, its monohydrate is located about 6.7 kJ mol^−1^ higher in energy than *cis*MOM‐1w_a_. The conversion barrier from *g*MOM‐1w_b_ to *cis*MOM‐1w_a_ is about 7.0 kJ mol^−1^, as shown in Figure 3. In the rotational spectrum, only *cis*MOM‐1w_a_ is detected. In both MOM monohydrates, the water molecule forms a H‐bond with the ether oxygen in MOM as a proton donor. The arrangement of *cis*MOM allows a second H‐bond to be formed in *cis*MOM‐1w_a_, making it energetically more stable.

**Figure 4:**
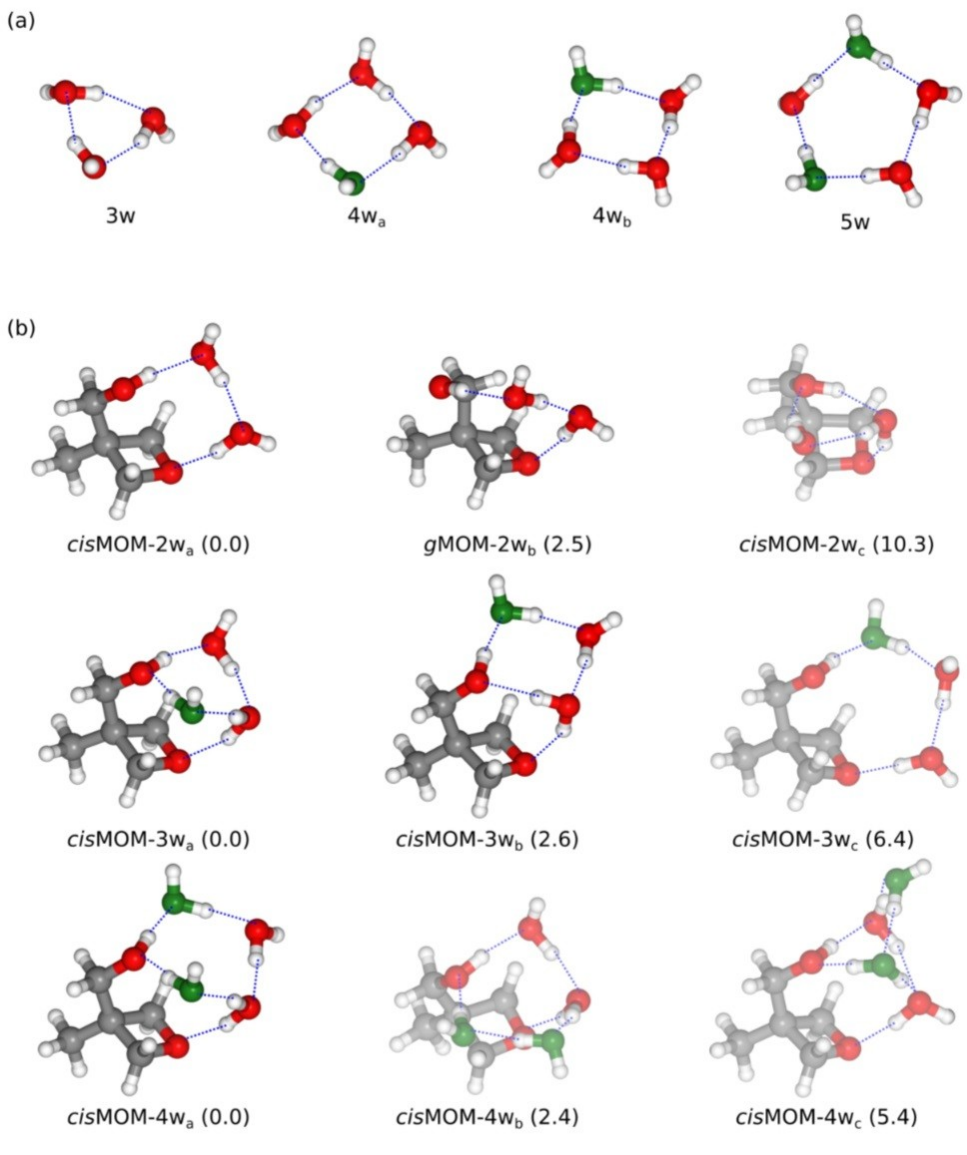
a) The most stable isomers of isolated water trimer (3w), tetramer (4w), and pentamer (5w).^[17]^ b) The three most stable isomers of the MOM dihydrate (MOM‐2w), trihydrate (MOM‐3w), and tetrahydrate (MOM‐4w). Their relative energies (in kJ mol^−1^) at the B3LYP‐D4/def2‐pVQZ level of theory after ZPE corrections are given in parentheses, where the most stable isomers are set to 0 kJ mol^−1^. For the MOM hydrates, the experimentally observed isomers are shown with clear geometries, while the unobserved isomers are displayed with faint colors. The green water molecules in MOM hydrates are the suggested water units that newly join the H‐bonding ring network, referring to *cis*MOM‐2w_a_, and the water units at the same positions in the pure water clusters are also displayed in green to make a comparison. The blue dashed lines indicate the primary hydrogen bonds within the structures.

For MOM dihydrates, there are three isomers predicted within a relative energy window of about 10 kJ mol^−1^, as shown in Figure 4b. The two most stable isomers are formed with *cis*MOM (*cis*MOM‐2w_a_) and *g*MOM (*g*MOM‐2w_b_), respectively. Similar to MOM‐1w, *cis*MOM‐2w_a_ is energetically more stable than *g*MOM‐2w_b_. They can be generated from *cis*MOM‐1w_a_ and *g*MOM‐1w_b_ by inserting one water unit between the OH group of MOM and the existing water molecule, or formed from the *cis*MOM and *g*MOM by directly complexing with water dimers (see their geometries in Figure 3). One end of the water dimer is the proton donor and is H‐bonded with the ether oxygen atom, while the other end binds with the OH group as a proton acceptor, forming an H‐bonded one‐dimensional (1D) chain between the two oxygen atoms in MOM. The energies of these two isomers differ by 2.5 kJ mol^−1^, and the conversion barrier from *g*MOM‐2w_b_ to *cis*MOM‐2w_a_ is 14.1 kJ mol^−1^. Both are formed in the gas jet and are probed in the rotational spectrum. In addition, according to the theoretical predictions, the third most stable energetic isomer (*cis*MOM‐2w_c_) exhibits a different H‐bonded network, where the water dimer and the OH group in MOM form a closed ring. This 2D ring structure resembles the geometry of the isolated water trimer (3w),^[14]^ as provided in Figure 3a. Each subunit in the ring network, including the two water molecules and the linked OH group, acts as a single donor‐single acceptor (DA). The water molecule close to the ether oxygen atom forms an additional H‐bond with it, making this water unit a double donor‐single acceptor (DDA). Although this arrangement allows it to form four primary H‐bonds instead of three as in *cis*MOM‐2w_a_ and *g*MOM‐2w_b_, the ring structure is not favored over the H‐bond chains, making it 10.3 kJ mol^−1^ higher than *cis*MOM‐2w_a_, and it is not observed in our experiment.

With one more water molecule joining the complex, the preference of the H‐bonding network changes. There are three isomers predicted for the MOM trihydrate within 10 kJ mol^−1^ as well (Figure 4b). However, the first two low‐energy isomers, *cis*MOM‐3w_a_ and *cis*MOM‐3w_b_, which are observed in the experiment, adopt the 2D cyclic arrangement above the oxetane ring. They are similar to *cis*MOM‐2w_c_ with an expanded H‐bonded ring. Their cyclic networks resemble the structures of the two most stable isomers of the water tetramer (4w_a_ and 4w_b_), respectively.[^48^, ^49^] Comparing with the geometries of MOM‐2w_a‐c_, it is evident that the expansion of the ring network can be linked with *cis*MOM‐2w_a_. The new water molecule could join from either side of the H‐bonding chain in *cis*MOM‐2w_a_, leading to the formation of a cyclic network in *cis*MOM‐3w_a_ and *cis*MOM‐3w_b_. The newly joined water unit is suggested and colored in green as shown in Figure 4b. Interestingly, the third isomer, *cis*MOM‐3w_c_, has a geometry that evolves from *cis*MOM‐2w_a_ with an elongated H‐bonded chain between the two oxygen atoms of MOM. In some similar molecular systems, such as formamide trihydrate, such chain arrangement is the preferred one.^[26]^ For MOM, to fit a water trimer chain, the oxetane plane is bent by about 10° away from the OH group of MOM, causing the O_4_⋅⋅⋅O_7_ distance in MOM to be 0.1 Å longer, compared to that in *cis*MOM‐2w_a_, *cis*MOM‐3w_a_, and *cis*MOM‐3w_b_. Compressed by the MOM backbone framework, this chain network in *cis*MOM‐3w_c_ is thus not favored. Even though it might be formed in the jet, it can presumably relax to *cis*MOM‐3w_b_ to close the H‐bonding network by overcoming a barrier of 3.8 kJ mol^−1^. By contrast, the ring networks in *cis*MOM‐3w_a_ and *cis*MOM‐3w_b_ reduce the tension in MOM, and the complexed water molecules also have better positions to establish contacts with the oxetane ring of MOM, not only to form the H‐bond with the ether oxygen, but also to form secondary H‐bonds with the adjacent C−H protons.

In the two low‐energy isomers of the water tetramer, 4w_a_ and 4w_b_, their energy difference is reported to be 3.6 kJ mol^−1^, calculated from stationary points at the CCSD(T)/aug‐cc‐pVDZ level of theory,^[49]^ and is re‐calculated to be at 3.2 kJ mol^−1^ at the B3LYP‐D4/def2‐QZVP level of theory in this work. Of them, the equilibrium structure of 4w_a_ possesses an *S_4_* molecular symmetry, where the ring network has a near‐square structure, and the configuration of the free O−H bonds is up‐down‐up‐down (denoted as “udud”) referring to the orientations of the free O−H bonds with respect to the plane of the cyclic network. 4w_b_ has a similar structure, but the arrangement of the free O−H bonds adopts uudd, making it a *C_i_* symmetry. The experimental observation of isolated 4w_a_ has been reported by Saykally and co‐workers using THz VRT spectroscopy,^[15]^ while 4w_b_ has yet to be observed, as it can easily transfer to 4w_a_ through a twofold flipping motion of free O−H bonds.[^48^, ^50^] With the presence of the solute molecules, the flipping motion of the free O−H bonds can be quenched, and either arrangement can be preferred depending on the water‐solute interactions. For instance, the H‐bonding network in benzophenone trihydrate resembles 4w_a_,^[31]^ while that in β‐propiolactone (BPL) tetrahydrate has a similar configuration as 4w_b_.^[32]^ In *cis*MOM‐3w_a_ and *cis*MOM‐3w_b_, where the C−O‐H moiety of the MOM replaces one water unit in 4w_a_ and 4w_b_, both arrangements have been locked and observed in the rotational spectrum. The energy difference between *cis*MOM‐3w_a_ and *cis*MOM‐3w_b_ is 2.6 kJ mol^−1^ at the B3LYP‐D4/def2‐QZVP level of theory, which is similar to that between the isolated 4w_a_ and 4w_b_ clusters.

Next, the MOM‐water system expands from the MOM trihydrate to tetrahydrate. The three most stable isomers are provided in Figure 4b, and their relative energies are within 6 kJ mol^−1^. Of them, only the global minimum geometry, *cis*MOM‐4w_a_, is identified in the rotational spectrum. The expanded H‐bonded ring in *cis*MOM‐4w_a_ resembles the geometry of the global minimum isomer of the pure water pentamer.[^16^, ^51^] It can be generated from both *cis*MOM‐3w_a_ and *cis*MOM‐3w_b_ by inserting a water unit in the cyclic network from either side of the MOM OH group. A similar arrangement has been observed in other gas‐phase water‐solute systems as well, such as Bz‐(H_2_O)_5_ ^[9]^ and BPL‐(H_2_O)_5_.^[32]^ The second lowest isomer, *cis*MOM‐4w_b_, has the same H‐bonding cyclic network as *cis*MOM‐4w_a_ and a similar arrangement of the free O−H bonds. The only difference is the water unit coordinated with the MOM ether oxygen, making *cis*MOM‐4w_b_ 2.4 kJ mol^−1^ higher in energy. *cis*MOM‐4w_b_ can be formed on the basis of *cis*MOM‐3w_a_ or *cis*MOM‐2w_a_. Overall, *cis*MOM‐4w_a_ is energetically and kinetically more favored in the aggregation process than *cis*MOM‐4w_b_.

**Figure 5:**
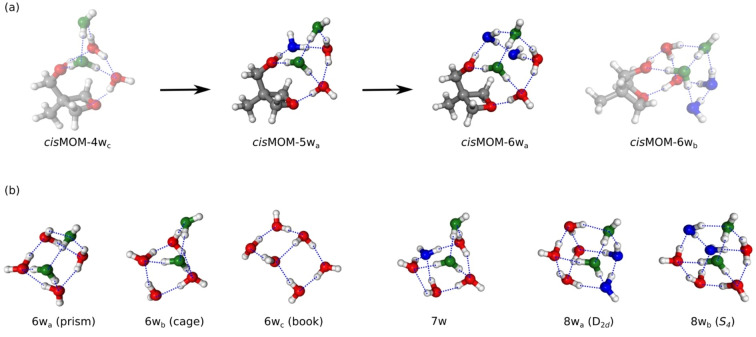
a) Structurally related 3D H‐bonding network in the MOM tetrahydrate (cisMOM‐4w_c_), to pentahydrate (cisMOM‐5w_a_), and further to hexahydrate (cisMOM‐6w_a_). The experimentally observed isomers (cisMOM‐5w_a_ and cisMOM‐6w_a_) are shown with clear geometries, while the unobserved isomers (cisMOM‐4w_c_ and cisMOM‐6w_b_) are displayed with faint colors. The suggested new water units in this process are colored in blue, whereas the green water molecules indicate the ones joined to form cisMOM‐4w_c_ in the former aggregation process, as depicted in Figure 4. b) The three most stable isomers of the pure water hexamer, known as the prism, cage, and book isomers, the most stable isomer of the water pentamer (7w), and the two lowest energy isomers of the water octamer (8w_a_ and 8w_b_), which have a molecular symmetry of D_2d_ and S_4_, respectively. The water units are colored in the manner to compare with the MOM hydrates. The blue dashed lines indicate the primary hydrogen bonds within the structures.

Two larger microsolvated clusters, MOM pentahydrate (*cis*MOM‐5w_a_) and hexahydrate (*cis*MOM‐6w_a_), are detected in the experiment as well. The assigned geometries are provided in Figure 5a, which are also the predicted global‐minimum isomers. Both of them have the multicyclic 3D H‐bonding arrangement instead of a 2D ring. However, as shown in Figures 4b and 5a, the 3D networks cannot be straightforwardly formed on basis of *cis*MOM‐4w_a_ but rather *cis*MOM‐4w_c_, which is located 5.4 kJ mol^−1^ higher than *cis*MOM‐4w_a_ (2.0 kJ mol^−1^ at the MP2/cc‐pVQZ level of theory) and is not observed experimentally. The suggested new water units are colored in blue in this process. In *cis*MOM‐4w_c_, the water unit above the ether oxygen atom acts as a single donor‐double acceptor (DAA) and both the neighboring water molecules act as DDA. The same arrangement is also present in *cis*MOM‐5w_a_, while in *cis*MOM‐4w_a_, the water unit above the ether group is a DDA and is coordinated with two DA water molecules. Therefore, *cis*MOM‐5w_a_ is more structurally related to *cis*MOM‐4w_c_. Nevertheless, also other formation pathways of *cis*MOM‐5w_a_ are plausible, such as complexation of *cis*MOM‐3w_b_ with a water dimer.

Previously, the characteristic multicyclic 3D H‐bonded structures of the lowest energetic isomers regarding the water hexamer and heptamer and their relative energies have been unambiguously resolved by Pérez et al. using rotational spectroscopy in combination with theoretical calculations,[^10^, ^18^] while the two lowest isomers of the nonpolar water octamer have been explored using mid‐IR spectroscopy^[52]^ and THz VRT spectroscopy.^[12]^ The main isomers are summarized in Figure 5b. Unlike the aforementioned MOM trihydrates (*cis*MOM‐3w_a_ and *cis*MOM‐3w_b_) and tetrahydrates (*cis*MOM‐4w_a_), the H‐bonding networks above the MOM ether group in *cis*MOM‐5w_a_ and *cis*MOM‐6w_a_ do not show similarities with the main isomers of the pure water clusters. Notably, by including the ether oxygen atom, the H‐bonding structures in *cis*MOM‐5w_a_ and *cis*MOM‐6w_a_ resemble the global minimum isomer of the water heptamer (7w) and the second lowest energy isomer of the water octamer (8w_b_), respectively, by replacing one edge with the two oxygen atoms of MOM. The H‐bonding structure in *cis*MOM‐5w_a_ is “prism”‐like as in 7w and can be presumably expanded from the “cage”‐like hexamer structure in *cis*MOM‐4w_c_. As a comparison, without the perturbation from MOM, the main low‐energy isomers of the water heptamer are proposed to be derived by adding a water unit to the edge of the hexamer prism.^[18]^ Moreover, for the gas‐phase Bz‐(H_2_O)_6,7_ clusters (Bz‐6w, Bz‐7w), the water hexamer bound to benzene in Bz‐6w is an inverted book isomer, while the H‐bonded structure in the main isomer of Bz‐7w is fully re‐arranged and forms an inserted‐cubic structure, where the benzene molecule replaces a water unit in the *S_4_*‐symmetry cube of the water octamer.^[20]^ The transition from a 2D ring to a 3D network in different microsolvated complexes prefers different H‐bonding structures, which are sensitive to the solute‐water interactions. But these H‐bonding structures evolve to similar “cube”‐like H‐bonding networks eventually.

Interestingly, even though the H‐bonding networks of *D_2d_* and *S_4_* symmetry are often found to be similar in energy (<0.5 kJ mol^−1^) for the isolated water octamer[^12^, ^53^] or similar molecular systems, such as phenol heptahydrates^[25]^ and benzene octahydrates,^[21]^ the H‐bonding networks in both Bz‐7w and MOM‐6w_a_ prefer the *S_4_*‐symmetry cube structure over the *D_2d_* cube. For the MOM hexahydrate, the isomer resembling the *D_2d_*‐symmetry cube H‐bond structure (*cis*MOM‐6w_b_) lies 5.1 kJ mol^−1^ above *cis*MOM‐6w_a_, and the O_4_⋅⋅⋅O_7_ distance in MOM is enlarged by 0.1 Å in MOM‐6w_b_. In addition, the benzene molecule in Bz‐7w and the MOM ether oxygen atom in *cis*MOM‐6w_a_ replace the same water unit in the H‐bonding network, which implies that the formation dynamics might have similarities.

According to the observed microsolvated species from the MOM monohydrate to hexahydrate, it is evident that the water H‐bonding structures always adapt themselves to the interaction space between the two oxygen atoms of the MOM monomer. In turn, the geometry and the conformational preference of MOM are influenced by the microsolvation environment. For 3,3‐disubstituted oxetanes, the *gauche* backbone arrangement is normally preferred in order to reduce the steric repulsion between the strained ring and the substituents.^[54]^ This is also observed for the MOM monomer, where *g*MOM (MOM‐I_a_) is found to be about three times more abundant than *cis*MOM (MOM‐II_a_) in the gas jet. However, as *cis*MOM seems to better incorporate small water clusters, it becomes more dominant in the microhydrated complexes compared to the *gauche* backbone. As a result, seven out of the eight experimentally observed MOM‐water complexes are formed with *cis*MOM, in agreement with the computed relative energies of the respective cluster sizes.

**Figure 6:**
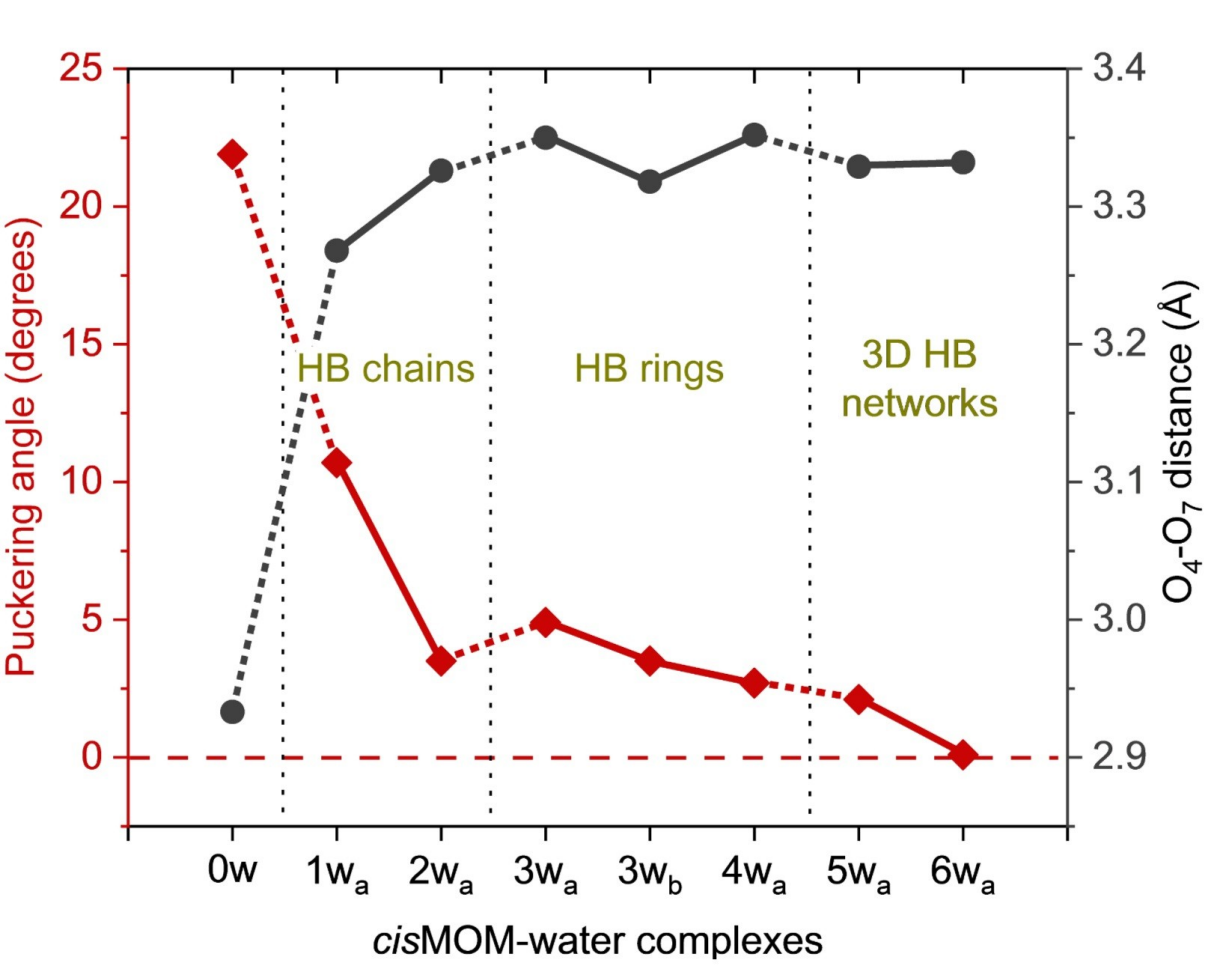
Evolution of the puckering angle of the oxetane ring (in degrees) and the O_4_‐O_7_ distance (in Å) in *cis*MOM with increasing degree of solvation, from the monomer MOM‐II_a_ (0w) to *cis*MOM‐6w_a_. The puckering angle denotes the deviation of the dihedral angle C_1_C_2_C_3_O_4_ from the planar form (180°).

In addition, with different degree of microsolvation, the MOM geometry also undergoes subtle changes, such as the O_4_⋅⋅⋅O_7_ distance and the planarity of the oxetane ring, as shown in Figure 6 for the *cis*MOM‐water complexes. A previous microwave spectroscopy study suggests that the unsubstituted oxetane has an effectively planar heavy‐atom backbone structure in the gas phase with a low puckering‐inversion barrier.^[55]^ The geometry remains planar in the microsolvation environment involving one or two water molecules.^[4]^ With the introduction of the substituents to the oxetane moiety, the ring is often distorted due to the increased eclipsing interactions. The puckering angle of the oxetane unit in MOM‐I_a_ is 11.0°, which is typical for a 3,3‐disubstituted oxetane (about 10°),^[54]^ whereas that in MOM‐II_a_ is 21.9°. The large puckering angle in MOM‐II_a_ can be attributed to the intramolecular H‐bonding interaction between the cyclic ether and the OH group. When one water molecule complexes with *cis*MOM, the intramolecular H‐bond is broken and the oxetane ring is released by about 10° to reduce the ring strain (see Figure 6). With increasing number of water molecules incorporated into the complexes, the strained oxetane ring gradually turns into a planar arrangement. The O_4_⋅⋅⋅O_7_ distance also changes accordingly in this process. This establishes the good flexibility of the oxetane ring, which helps with its binding affinity. Collectively, this provides us with valuable insight into the structure‐property relationships.

## Conclusion

In summary, a rotational spectroscopy study of the gas‐phase MOM‐(H_2_O)_*n*_ (*n*=0–6) clusters is reported, accompanied by exhaustive theoretical calculations. In the jet expansion, two conformations are adopted by the MOM monomer, namely *gauche*‐ and *cis*‐O_4_C_1_C_6_O_7_. As the *cis* conformer provides a better interaction space, most of the observed MOM‐water clusters are formed with the *cis* conformation, despite that the *gauche* conformer is energetically favored. Starting with the MOM monohydrate and dihydrate, 1D H‐bonded chains are formed in between the two oxygen atoms of MOM. The interplay between the MOM conformation and the water chain is observed. For the MOM trihydrate and tetrahydrate, 2D H‐bonded rings are more favored instead of the 1D chain, restricted by the space between the two MOM oxygen atoms. The H‐bonded rings resemble the isolated water tetramer and pentamer, with the hydroxyl group of MOM replacing one water unit. Most intriguingly, for the MOM pentahydrate and hexahydrate, 3D networks from H‐bond rearrangements become dominant, which resemble the isolated water heptamer and octamer, respectively.

Overall, the conformations of the MOM monomer and the water H‐bonding network adapt to each other in the above‐discussed MOM‐water clusters. The evolution of the H‐bonding networks to 3D cubes established in this work sheds more light on the H‐bonding cooperativity in larger microsolvated molecular systems, going beyond a variety of previously reported 1D/2D H‐bond arrangements investigated with microwave spectroscopy. Even though MOM is strongly integrated in these clusters, there is a clear tendency for the favored H‐bonding networks to mimic the main isomers of the pure water clusters of similar size. This suggests that the frameworks of these H‐bond arrangements have an advantage to reduce the energy of such molecular systems.

## Conflict of interest

The authors declare no conflict of interest.

1

## Supporting information

As a service to our authors and readers, this journal provides supporting information supplied by the authors. Such materials are peer reviewed and may be re‐organized for online delivery, but are not copy‐edited or typeset. Technical support issues arising from supporting information (other than missing files) should be addressed to the authors.

Supporting Information

## Data Availability

The data that support the findings of this study are provided in the Supporting Information of this article and available from the corresponding author upon reasonable request.
