# Supplementary material for: Microhydrated 3‐Methyl‐3‐oxetanemethanol: Evolution of the Hydrogen‐Bonding Network from Chains to Cubes
Source: Angew Chem Int Ed Engl. 2022 Nov 9;61(49):e202210819. doi: 10.1002/anie.202210819 (PMC10099544; doi:10.1002/anie.202210819)
Supplement: Supplementary file 1 — Supporting Information [file ANIE-61-0-s001.pdf]

Angewandte Chemie
Eine Zeitschrift der Gesellschaft Deutscher Chemiker

Supporting Information

Microhydrated 3-Methyl-3-oxetanemethanol: Evolution of the Hydrogen-Bonding Network from Chains to Cubes

*W. Sun, M. Schnell**

Table of Contents

List of Tables and Figures 1

1. Method Summary 4

2. Results 5

   2.1. MOM monomer 5

   2.2. MOM monohydrate 12

   2.3. MOM dihydrate 14

   2.4. MOM trihydrate 17

   2.5. MOM tetrahydrate 20

   2.6. MOM pentahydrate 24

   2.7. MOM hexahydrate 27

   2.8. Observed Transition Frequencies 31

List of Tables and Figures

Figure S1. Potential energy curves obtained by rotating the hydroxyl group about the C_6-O_7 bond in the *gauche*- (a) and *cis*-$O_4C_1C_6O_7$ (b) conformations of MOM monomer at the B3LYP-D4/def2-QZVP level of theory. 5

Table S1. Spectroscopic parameters of the MOM monomer calculated at the B3LYP-D4/def2-QZVP level of theory. 5

Figure S2. Potential energy curves obtained by rotating the hydroxyl group about the C_6-O_7 bond in the *gauche*- (a) and *cis*-$O_4C_1C_6O_7$ (b) conformations of MOM monomer at the MP2/cc-pVQZ level of theory. 5

Table S2. Spectroscopic parameters of the MOM monomer calculated at the MP2/cc-pVQZ level of theory. 6

Figure S3. Part of the broadband spectrum of the parent species of MOM-I_a and MOM-II_a collected with 2.1×10^6 FIDs. 6

Figure S4. Highlighted rotational transition J_{kakc}: 3_{22} — 3_{12} for the ^{13}C isotopologues of MOM-I_a. 6

Figure S5. Highlighted rotational transition 4_{13} — 3_{12} for the ^{13}C isotopologues of MOM-II_a. 7

Figure S6. Part of the broadband spectrum the $^2H_8$ isotopologues of MOM-I_a and MOM-II_a collected with 1.9×10^6 FIDs. 7

Table S3. Ground state spectroscopic constants of the parent species of MOM-I_a and MOM-II_a. 7

Table S4. Ground state spectroscopic constants of the ^{13}C, ^{18}O and $^2H_8$ isotopologues of MOM-I_a. 8

Table S5. Ground state spectroscopic constants of the ^{13}C, ^{18}O and $^2H_8$ isotopologues of MOM-II_a. 8

Table S6. Spectroscopic parameters of the MOM-II_a calculated at the B3LYP-D4/def2-QZVP and MP2/cc-pVQZ levels of theory, where the O···O distance is constrained at 2.933 Å, together with the 9

Table S7. Substitution coordinates (r_s) of heavy atoms (^{13}C and ^{18}O) and H_8 in the principal inertial axes systems of the parent species of MOM-I_a and equilibrium coordinates (r_e) calculated at the B3LYP-D4/def2-QZVP and MP2/cc-pVQZ levels of theory, respectively. 10

Table S8. Substitution coordinates (r_s) of heavy atoms (^{13}C and ^{18}O) in the principal inertial axes systems of the parent species of MOM-II_a and equilibrium coordinates (r_e) optimized at the B3LYP-D4/def2-QZVP and MP2/cc-pVQZ levels of theory, respectively, while the O_4···O_7 distance is fixed at 2.933 Å. 10

Figure S7. Potential energy curves obtained by rotating the hydroxyl group about the C_6-O_7 bond in the *cis*-$O_4C_1C_6O_7$ (b) conformations of the MOM monomer at various levels of theory, including (a). B3LYP-D4/def2-QZVP(B3D4) and CCSD(T)//B3LYP-D4, which is single-point energy calculations with the CCSD(T)/cc-pVTZ functional performed on the scanned geometries of the B3LYP-D4 PEC; (b). MP2/cc-pVQZ (mp2) and CCSD(T)//MP2, which is single-point energy calculation with the CCSD(T)/cc-pVTZ functional performed on the scanned geometries of the MP2 PEC; (c). M06-2X-D3/def2-QZVP (M06-2X-D3) and PW6B95-D4/ def2-QZVP (PW6B95-D4); (d) B2PLYP-D4/def2-QZVP (B2PLYP-D4) and PWPB95-D4/def2-QZVP (PWPB95-D4) levels of theory. 11

Figure S8. Relative energies (in kJ/mol) of the five conformers of the MOM monomer calculated at different levels of theory, including (a). B3LYP-D4/def2-QZVP (A1), CCSD(T)/cc-pVTZ//B3LYP-D4/def2-QZVP (A2), DLPNO-CCSD(T)/cc-pVTZ//B3LYP-D4/def2-QZVP (A3), and DLPNO-CCSD(T)/cc-pVQZ//B3LYP-D4/def2-QZVP (A4); (b). MP2/cc-pVQZ (B1), CCSD(T)/cc-pVTZ//MP2/cc-pVQZ (B2), DLPNO-CCSD(T)/cc-pVTZ// MP2/cc-pVQZ (B3), and DLPNO-CCSD(T)/cc-pVQZ// MP2/cc-pVQZ (B4); (c). M06-2X/def2-QZVP (C1), PW6B95-D4/def2-QZVP (C2), B2PLYP-D4/def2-QZVP (C3), and PWPB95-D4/def2-TZVP (C4). Note that the CCSD(T)//B3LYP-D4, DLPNO-CCSD(T)//B3LYP-D4, CCSD(T)//MP2, and DLPNO-CCSD(T)//MP2 functionals are used for performing single-point energy calculations with the CCSD(T) or DLPNO-CCSD(T) functional on the geometries optimized at the B3LYP-D4/def2-QZVP and MP2/cc-pVQZ levels of theory, respectively. 12

Table S9. Cartesian coordinates (Å) for the equilibrium structure of *cis*MOM-1w_a obtained at the B3LYP-D4/def2-QZVP level of theory. 12

Table S10. Cartesian coordinates (Å) for the equilibrium structure of *g*MOM-1w_b obtained at the B3LYP-D4/def2-QZVP level of theory. 13

Figure S9. Part of the broadband spectrum of *cis*MOM-1w_a collected with 2.4×10^6 FIDs. The upper trace is obtained from the experiment and the bottom trace is the simulation based on the fit. 13

Table S11. Spectroscopic constants for *cis*MOM-1w_a and *g*MOM-1w_b obtained at the B3LYP-D4/def2-QZVP level of theory. 13

Table S12. Spectroscopic constants for *cis*MOM-1w_a and *g*MOM-1w_b obtained at the MP2/cc-pVQZ level of theory. 14

Table S13. Experimental spectroscopic constants of *cis*MOM-1w_a. 14

Table S14. Cartesian coordinates (Å) for the equilibrium structure of *cis*MOM-2w_a obtained at the B3LYP-D4/def2-QZVP level of theory. 14

Table S15. Cartesian coordinates (Å) for the equilibrium structure of *g*MOM-2w_b obtained at the B3LYP-D4/def2-QZVP level of theory. 15

Table S16. Cartesian coordinates (Å) for the equilibrium structure of *cis*MOM-2w_c obtained at the B3LYP-D4/def2-QZVP level of theory. 15

Figure S10. Part of the broadband spectrum of *cis*MOM-2w_a and *g*MOM-2w_b collected with 2.4×10^6 FIDs. 16

Table S17. Spectroscopic constants for *cis*MOM-2w_a, *g*MOM-2w_b, and *cis*MOM-2w_c obtained at the B3LYP-D4/def2-QZVP level of theory. 16

Table S18. Spectroscopic constants for *cis*MOM-2w_a, *g*MOM-2w_b, and *cis*MOM-2w_c obtained at the MP2/cc-pVQZ level of theory. .. 16

Table S19. Experimental spectroscopic constants of *cis*MOM-2w_a and *g*MOM-2w_b. 16

Table S20. Cartesian coordinates (Å) for the equilibrium structure of *cis*MOM-3w_a obtained at the B3LYP-D4/def2-QZVP level of theory. 17

Table S21. Cartesian coordinates (Å) for the equilibrium structure of *cis*MOM-3w_b obtained at the B3LYP-D4/def2-QZVP level of theory. 17

Table S22. Cartesian coordinates (Å) for the equilibrium structure of *cis*MOM-3w_c obtained at the B3LYP-D4/def2-QZVP level of theory. 18

Figure S11. Part of the broadband spectrum of *cis*MOM-3w_a and *cis*MOM-3w_b collected with 3.1×10^6 FIDs. 19

Table S23. Spectroscopic constants for *cis*MOM-3w_a, *cis*MOM-3w_b, and *cis*MOM-3w_c obtained at the B3LYP-D4/def2-QZVP level of theory. 19

Table S24. Spectroscopic constants for *cis*MOM-3w_a, *cis*MOM-3w_b, and *cis*MOM-3w_c obtained at the MP2/cc-pVQZ level of theory. 19

Table S25. Experimental spectroscopic constants of *cis*MOM-3w_a and *cis*MOM-3w_b. 19

Table S26 Cartesian coordinates (Å) for the equilibrium structure of *cis*MOM-4w_a obtained at the B3LYP-D4/def2-QZVP level of theory. 20

Table S27. Cartesian coordinates (Å) for the equilibrium structure of *cis*MOM-4w_b obtained at the B3LYP-D4/def2-QZVP level of theory. 20

Table S28. Cartesian coordinates (Å) for the equilibrium structure of *cis*MOM-4w_c obtained at the B3LYP-D4/def2-QZVP level of theory. 21

Figure S12. Part of the broadband spectrum of *cis*MOM-4w_a collected with 0.5×10^6 FIDs. 22

Table S29. Spectroscopic constants for *cis*MOM-4w_a, *cis*MOM-4w_b, and *cis*MOM-4w_c obtained at the B3LYP-D4/def2-QZVP level of theory. 22

Table S30. Spectroscopic constants for *cis*MOM-4w_a, *cis*MOM-4w_b, and *cis*MOM-4w_c obtained at the MP2/cc-pVQZ level of theory. 22

Table S31. Experimental spectroscopic constants of *cis*MOM-4w_a. 22

Table S32. Spectroscopic constants for *cis*MOM-4w_d – *cis*MOM-4w_h obtained at the B3LYP-D4/def2-QZVP level of theory. 23

Figure S13. Five high-energy isomers of the MOM tetrahydrate within an energy window of 8 kJ/mol predicted at the B3LYP-D4/def2-QZVP level of theory. The relative energies (in kJ/mol) to *cis*MOM-4w_a are given in the parentheses. 23

Figure S14. Nine isomers of the MOM pentahydrate within an energy window of 6 kJ/mol predicted at the B3LYP-D4/def2-QZVP level of theory. The relative energies (in kJ/mol) are given in the parentheses. 24

Table S33. Spectroscopic constants for *cis*MOM-5w_a – *cis*MOM-5w_i obtained at the B3LYP-D4/def2-QZVP level of theory. 24

Table S34. Cartesian coordinates (Å) for the equilibrium structure of *cis*MOM-5w_a obtained at the B3LYP-D4/def2-QZVP level of theory. 24

Table S35. Cartesian coordinates (Å) for the equilibrium structure of *cis*MOM-5w_b obtained at the B3LYP-D4/def2-QZVP level of theory. 25

Table S36. Cartesian coordinates (Å) for the equilibrium structure of *cis*MOM-5w_c obtained at the B3LYP-D4/def2-QZVP level of theory. 25

Figure S15. Part of the broadband spectrum of *cis*MOM-5w_a collected with 0.5×10^6 FIDs. 26
Table S37. Spectroscopic constants for *cis*MOM-5w_a, *cis*MOM-5w_b, and *cis*MOM-5w_c obtained at the MP2/cc-pVQZ level of theory. 26
Table S38. Experimental spectroscopic constants of *cis*MOM-5w_a. 26
Table S39. Spectroscopic constants for *cis*MOM-6w_a – *cis*MOM-6w_i obtained at the B3LYP-D4/def2-QZVP level of theory. 27
Figure S16. Nine isomers of the MOM hexahydrate within an energy window of 8 kJ/mol predicted at the B3LYP-D4/def2-QZVP level of theory. The relative energies (in kJ/mol) are given in the parentheses. Note that in the main text, *cis*MOM-6w_c is denoted as *cis*MOM-6w_a'. 28
Table S40. Cartesian coordinates (Å) for the equilibrium structure of *cis*MOM-6w_a obtained at the B3LYP-D4/def2-QZVP level of theory. 28
Table S41. Cartesian coordinates (Å) for the equilibrium structure of *cis*MOM-6w_b obtained at the B3LYP-D4/def2-QZVP level of theory. 29
Table S42. Cartesian coordinates (Å) for the equilibrium structure of *cis*MOM-6w_c obtained at the B3LYP-D4/def2-QZVP level of theory. 29
Figure S17. Part of the broadband spectrum of *cis*MOM-6w_a collected with 0.5×10^6 FIDs. 30
Table S43. Spectroscopic constants for *cis*MOM-6w_a, *cis*MOM-6w_b, and *cis*MOM-6w_c obtained at the MP2/cc-pVQZ level of theory. 30
Table S44. Experimental spectroscopic constants of *cis*MOM-6w_a. 30
Table S45. Assigned transitions for the parent species of MOM-I_a. 31
Table S46. Assigned transitions for the $^{13}C_1$ isotopologue of MOM-I_a. 32
Table S47. Assigned transitions for the $^{13}C_2$ isotopologue of MOM-I_a. 33
Table S48. Assigned transitions for the $^{13}C_3$ isotopologue of MOM-I_a. 33
Table S49. Assigned transitions for the $^{18}O_4$ isotopologue of MOM-I_a. 34
Table S50. Assigned transitions for the $^{13}C_5$ isotopologue of MOM-I_a. 34
Table S51. Assigned transitions for the $^{13}C_6$ isotopologue of MOM-I_a. 34
Table S52. Assigned transitions for the $^{18}O_7$ isotopologue of MOM-I_a. 35
Table S53. Assigned transitions for the $^{2}H_8$ isotopologue of MOM-I_a. 35
Table S54. Assigned transitions for the parent species of MOM-II_a. 36
Table S55. Assigned transitions for the $^{13}C_1$ isotopologue of MOM-II_a. 37
Table S56. Assigned transitions for the $^{13}C_2$/$^{13}C_3$ isotopologue of MOM-II_a. 37
Table S57. Assigned transitions for the $^{18}O_4$ isotopologue of MOM-II_a. 37
Table S58. Assigned transitions for the $^{13}C_5$ isotopologue of MOM-I_a. 38
Table S59. Assigned transitions for the $^{13}C_6$ isotopologue of MOM-II_a. 38
Table S60. Assigned transitions for the $^{18}O_7$ isotopologue of MOM-II_a. 38
Table S61. Assigned transitions for the $^{2}H_8$ isotopologue of MOM-II_a. 39
Table S62. Assigned transitions for the parent species of *cis*MOM-1w_a. 39
Table S63. Assigned transitions for the parent species of *cis*MOM-2w_a. 40
Table S64. Assigned transitions for the parent species of *g*MOM-2w_b. 41
Table S65. Assigned transitions for the parent species of *cis*MOM-3w_a. 42
Table S66. Assigned transitions for the parent species of *cis*MOM-3w_b. 43
Table S67. Assigned transitions for the parent species of *cis*MOM-4w_a. 45
Table S68. Assigned transitions for the parent species of *cis*MOM-5w_a. 46
Table S69. Assigned transitions for the parent species of *cis*MOM-6w_a. 47

1. Method Summary

1.1. Experimental details

The pure rotational spectrum of MOM was measured in the frequency range of 18 – 26 GHz with our segmented K-band chirped-pulse Fourier transform microwave (CP-FTMW) spectrometer.[1] MOM (purity: 98%) is commercially available from Sigma-Aldrich and was used without further purification. The liquid sample was placed in a custom-made reservoir, which is a part of the solenoid nozzle, and maintained at 40 °C. Neon at a backing pressure of ~2 bar was used as the carrier gas to deliver the sample vapor to the microwave spectrometer. The gas mixture was supersonically expanded into the vacuum chamber via a pulsed valve (General valve Series 9) operating at a repetition rate of 10 Hz. The rotationally cooled molecular ensemble was polarized by a pulse train of ten 800 MHz chirped-pulse segments covering the entire bandwidth of 8 GHz. The free induction decay (FID) in the time domain was collected for each segment after the excitation, processed by the detection circuit, and digitized on a 3.2 GS/s digitizer card. Each gas pulse got polarized three times by the pulse train, leading to a 30 Hz effective repetition rate of the experiment. In total, 2.1 million FID acquisitions were co-added and averaged for each segment. The time-domain signal was transformed into the frequency domain by means of the fast Fourier transformation (FFT) algorithm. The obtained ten frequency segments were concatenated together to achieve the final rotational spectrum from 18 – 26 GHz, the resolution of which is ~100 kHz.

Afterwards, a second reservoir containing distilled water was installed in the gas line situated upstream outside the vacuum chamber. Water vapor was then carried by neon and mixed with MOM kept in the internal nozzle reservoir. A spectrum averaged with 2.4 million FIDs was obtained. In addition, the spectrum of MOM with water between 2 – 8 GHz was measured as well under a similar sample condition using our COMPACT microwave spectrometer.[2] In this experiment, the molecular beam was polarized by microwave chirps spanning 2 – 8 GHz with an effective repetition rate of 64 Hz. In total, 3 million FIDs were collected on a fast oscilloscope with a sampling rate of 25 GS/s, and the spectral resolution is ~25 kHz.

1.2. Computational calculations

Quantum-chemical calculations were performed with the ORCA 4.2.1 program unless otherwise mentioned.[3] The conformational arrangements of the MOM monomer were investigated by scanning the torsional potential around the C_1-C_6 and C_6-O_7 bonds at the B3LYP/def2-QZVP level of theory[4-7] in combination with Grimme's D4 dispersion corrections[8] and at the MP2/cc-pVQZ level of theory.[9] The obtained local minima were fully optimized at the same level of theory. The geometries of the complexes of MOM with up to six water molecules, MOM-$(H_2O)_n$ (n = 1 – 6), were exhaustively searched using the GFN-xTB method with the CREST routine[10-12] and the obtained preliminary geometries were further optimized at the B3LYP-D4/def2-QZVP level of theory in combination with frequency calculations. The main isomers of the hydrate complexes are also optimized at the MP2/cc-pVQZ level of theory.

References

[1] M. Fatima, C. Pérez, B. E. Arenas, M. Schnell, A. L. Steber, *Phys. Chem. Chem. Phys.* **2020**, *22*, 17042-17051.
[2] D. Schmitz, V. Alvin Shubert, T. Betz, M. Schnell, *J. Mol. Spectrosc.* **2012**, *280*, 77-84.
[3] F. Neese, *Wiley Interdiscip. Rev.: Comput. Mol. Sci.* **2018**, *8*, e1327.
[4] A. D. Becke, *Phys. Rev. A* **1988**, *38*, 3098-3100.
[5] A. D. Becke, *J. Chem. Phys.* **1993**, *98*, 5648-5652.
[6] P. J. Stephens, F. J. Devlin, C. F. Chabalowski, M. J. Frisch, *J. Phys. Chem. Lett.* **1994**, *98*, 11623-11627.
[7] F. Weigend, R. Ahlrichs, *Phys. Chem. Chem. Phys.* **2005**, *7*, 3297-3305.
[8] E. Caldeweyher, J.-M. Mewes, S. Ehlert, S. Grimme, *Phys. Chem. Chem. Phys.* **2020**, *22*, 8499-8512.
[9] C. Møller, M. S. Plesset, *Phys. Rev.* **1934**, *46*, 618-622.
[10] S. Grimme, C. Bannwarth, P. Shushkov, *J. Chem. Theory Comput.* **2017**, *13*, 1989-2009.
[11] C. Bannwarth, S. Ehlert, S. Grimme, *J. Chem. Theory Comput.* **2019**, *15*, 1652-1671.
[12] P. Pracht, F. Bohle, S. Grimme, *Phys. Chem. Chem. Phys.* **2020**, *22*, 7169-7192.

2. Results

2.1. MOM monomer

Figure S1. Potential energy curves obtained by rotating the hydroxyl group about the C_6-O_7 bond in the *gauche*- (a) and *cis*-$O_4C_1C_6O_7$ (b) conformations of MOM monomer at the B3LYP-D4/def2-QZVP level of theory.

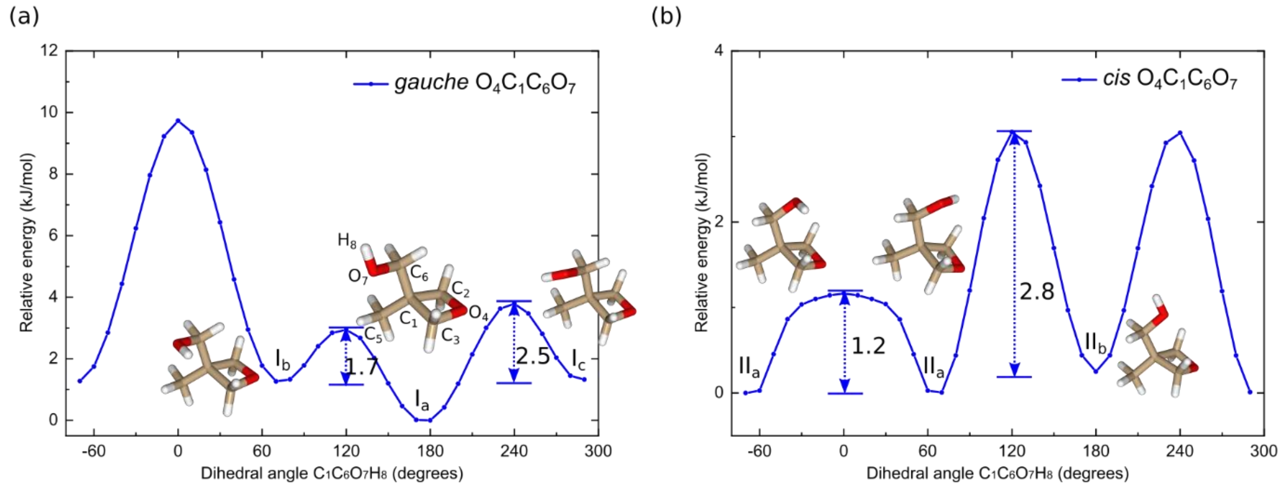

Table S1. Spectroscopic parameters of the MOM monomer calculated at the B3LYP-D4/def2-QZVP level of theory.

Parameters	I_a	I_b	I_c	II_a	II_b
A [MHz]	4147.4	4122.8	4110.2	3320	3428.5
B [MHz]	2083.2	2059.8	2070.5	2568	2525.2
C [MHz]	1839.6	1820.3	1819.5	1889.4	1889.3
$\lvert\mu_a\rvert$ [D]	1.8	0.2	1.2	0.2	2.4
$\lvert\mu_b\rvert$ [D]	0.4	0.5	2.5	2.1	2.6
$\lvert\mu_c\rvert$ [D]	1.4	0.2	0.6	1.1	0
ΔE [kJ/mol]	0.0	1.3	1.3	1.6	1.8
ΔE_{ZPE} [kJ/mol]	0.0	1.6	1.4	1.4	1.7

Figure S2. Potential energy curves obtained by rotating the hydroxyl group about the C_6-O_7 bond in the *gauche*- (a) and *cis*-$O_4C_1C_6O_7$ (b) conformations of MOM monomer at the MP2/cc-pVQZ level of theory.

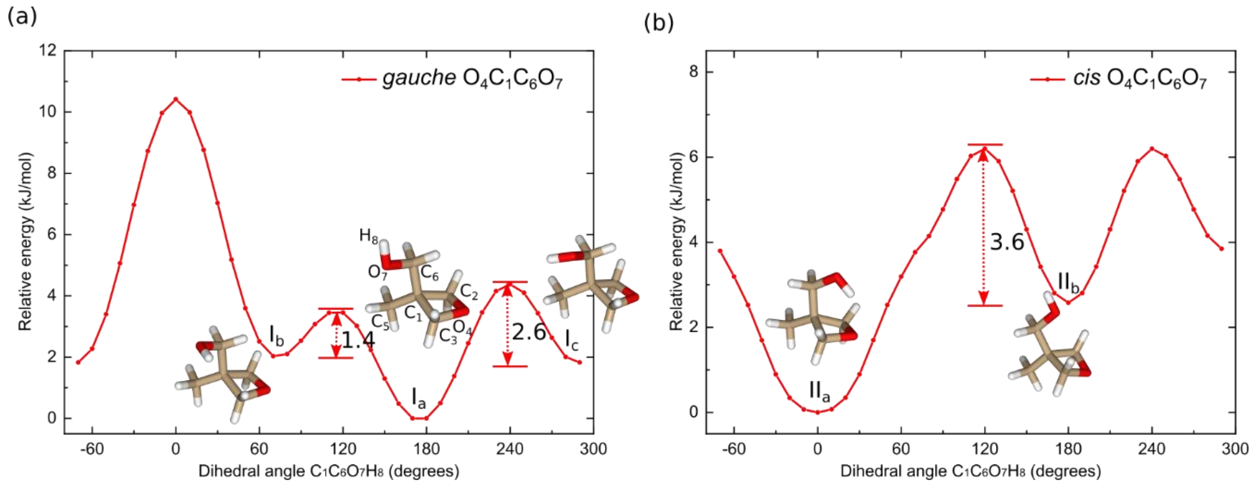

Table S2. Spectroscopic parameters of the MOM monomer calculated at the MP2/cc-pVQZ level of theory.

Parameters	I_a	I_b	I_c	II_a	II_b
A [MHz]	4097.2	4063.1	4055.9	3222.1	3561.1
B [MHz]	2163.4	2140.5	2151.5	2928.6	2491.0
C [MHz]	1876.2	1855.3	1855.3	2010.3	1899.4
$\|\mu_a\|$ [D]	1.7	0.1	1.2	2.9	2.6
$\|\mu_b\|$ [D]	0.4	0.7	2.6	0.8	2.4
$\|\mu_c\|$ [D]	1.3	0.2	0.6	0.0	0.0
ΔE [kJ/mol]	0.4	2.3	2.2	0	2.8

Figure S3. Part of the broadband spectrum of the parent species of MOM-I$_a$ and MOM-II$_a$ collected with 2.1×10^6 FIDs.

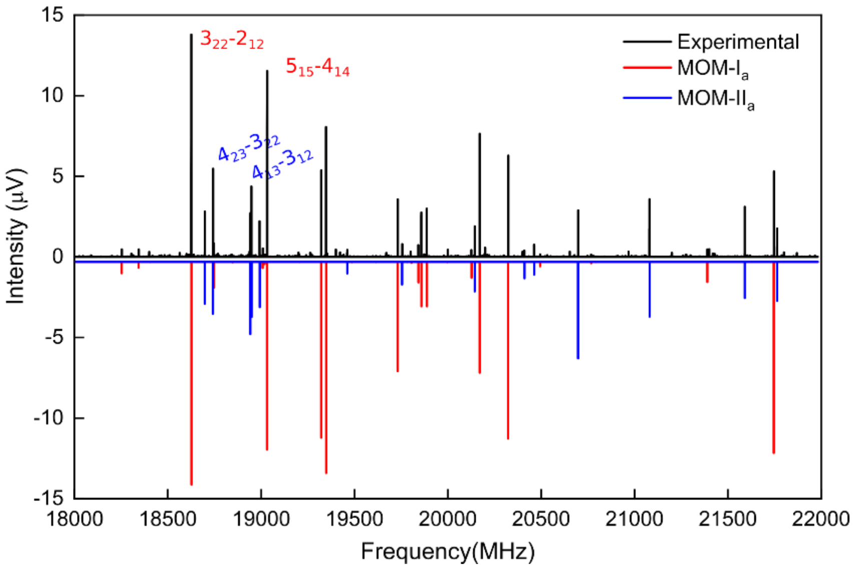

Figure S4. Highlighted rotational transition J$_{KaKc}$: 3_{22} — 3_{12} for the ^{13}C isotopologues of MOM-I$_a$.

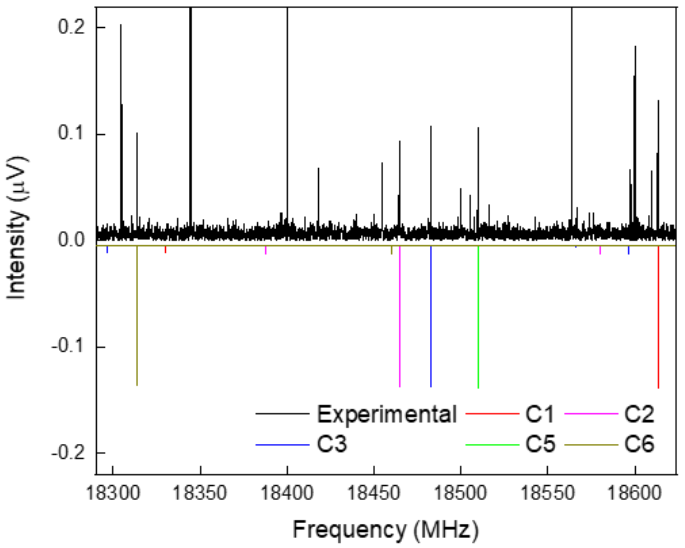

Figure S5. Highlighted rotational transition 4_{13} — 3_{12} for the ^{13}C isotopologues of MOM-II$_a$.

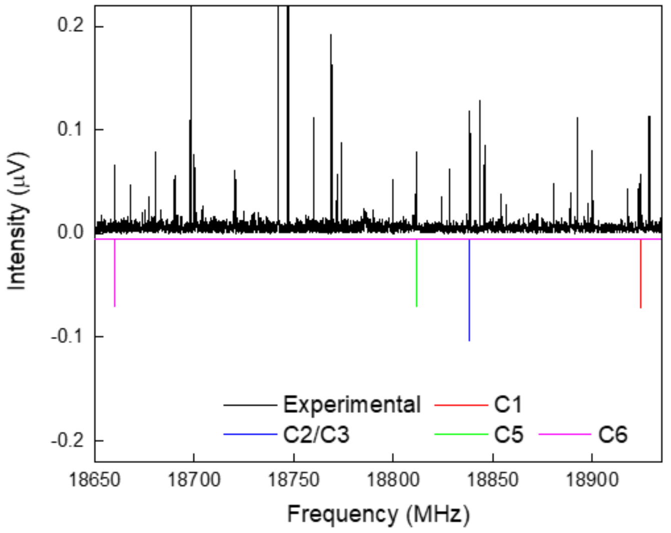

Figure S6. Part of the broadband spectrum the $^2H_8$ isotopologues of MOM-I$_a$ and MOM-II$_a$ collected with 1.9×10^6 FIDs.

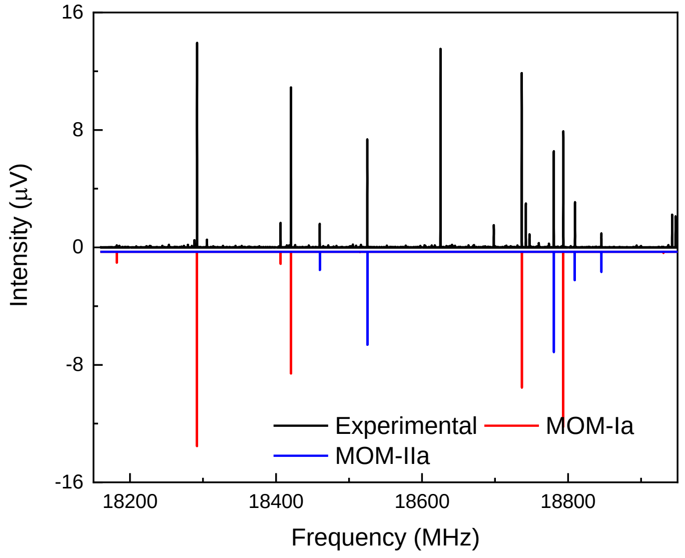

Table S3. Ground state spectroscopic constants of the parent species of MOM-I$_a$ and MOM-II$_a$.

Parameters	MOM-I$_a$	MOM-II$_a$
A [MHz]	4097.60493(37)[a]	3144.9601(10)[a]
B [MHz]	2110.84467(28)	2874.01219(92)
C [MHz]	1846.51469(27)	1966.32603(71)
Δ_J [kHz]	0.6099(31)	0.489(20)
Δ_{JK} [kHz]	-2.143(12)	2.740(69)
Δ_K [kHz]	5.557(14)	-2.770(63)
δ_J [kHz]	0.1974(14)	0.1320(90)
δ_K [kHz]	0.814(48)	0.967(28)
a-type[c]	+++	+++
b-type[b]	+	++

c-type[b]	+++	NO
P_{cc} [μÅ^2] [d]	…	39.76
σ [kHz] [e]	5.3	8.3
N [f]	83	64

[a] Standard errors within parentheses are expressed in units of the last two digits. [b] Values in brackets fixed to the corresponding values of the parent species. [c] Strength of the observed type of transitions: +++ (strong), ++ (medium), + (weak), NO (not observed). [d] Planar moment along the molecule's c-axis, which is calculated using the formula, $P_{cc} = (I_a + I_b - I_c)/2$. [e] Root-mean-square deviation of the fit. [f] number of the lines in the fit.

Table S4. Ground state spectroscopic constants of the ^{13}C, ^{18}O and $^{2}H_8$ isotopologues of MOM-I_a.

Parameters	$^{13}C_1$	$^{13}C_2$	$^{13}C_3$	$^{18}O_4$
A [MHz]	4093.4456(10)	4058.23930(66)	4071.73721(47)	4067.3304(51)
B [MHz]	2110.99140(91)	2096.68980(58)	2089.21194(54)	2051.8292(18)
C [MHz]	1845.8166(12)	1841.33246(82)	1834.35752(71)	1795.4383(24)
Δ_J [kHz]	0.611(14)	0.5872(92)	0.6068(86)	0.623(29)
Δ_{JK} [kHz]	-2.160(53)	-2.008(34)	-1.903(19)	-2.16(24)
Δ_K [kHz]	[5.557] [b]	[5.557]	[5.557]	[5.557]
δ_J [kHz]	0.2107(73)	0.1837(48)	0.1811(44)	0.192(13)
δ_K [kHz]	[0.814]	[0.814]	[0.814]	[0.814]
σ [kHz] [c]	5.2	3.9	3.7	8.8
N [d]	20	24	26	15
Parameters	$^{13}C_5$	$^{13}C_6$	$^{18}O_7$	$^{2}H_8$
A [MHz]	4073.49150(60)	3996.49573(91)	4071.1309(30)	4053.64302(28)
B [MHz]	2096.45113(62)	2108.17136(77)	2032.5110(12)	2043.67903(37)
C [MHz]	1837.14531(85)	1824.5321(11)	1782.3569(18)	1786.64346(33)
Δ_J [kHz]	0.5736(99)	0.563(12)	0.535(21)	0.6485(46)
Δ_{JK} [kHz]	-1.832(26)	-1.798(46)	-2.12(17)	-2.092(17)
Δ_K [kHz]	[5.557]	[5.557]	[5.557]	5.757(18)
δ_J [kHz]	0.2011(49)	0.1902(63)	0.1904(98)	0.1808(18)
δ_K [kHz]	[0.814]	[0.814]	[0.814]	0.682(65)
σ [kHz]	4.0	5.4	6.7	2.3
N	23	26	17	44

[a] Standard errors within parentheses are expressed in units of the last two digits. [b] Values in brackets fixed to the corresponding values of the parent species. [c] Root-mean-square deviation of the fit. [d] number of the lines in the fit.

Table S5. Ground state spectroscopic constants of the ^{13}C, ^{18}O and $^{2}H_8$ isotopologues of MOM-II_a.

Parameters	$^{13}C_1$	$^{13}C_2$/$^{13}C_3$	$^{18}O_4$	$^{13}C_5$

A [MHz]	3141.2453(10)	3115.84384(60)	3042.02179(43)	3124.98710(50)
B [MHz]	2871.5088(11)	2851.90938(79)	2869.69970(62)	2856.84751(68)
C [MHz]	1963.6748(14)	1960.42887(94)	1923.48236(43)	1950.54115(66)
Δ_J [kHz]	0.330(28)	0.413(17)	0.463(12)	0.381(13)
Δ_{JK} [kHz]	[2.740][b]	[2.740]	[2.740]	[2.740]
Δ_K [kHz]	[-2.770]	[-2.770]	[-2.770]	[-2.770]
δ_J [kHz]	0.0745(99)	0.1003(61)	0.1131(54)	0.0816(55)
δ_K [kHz]	[0.967]	[0.967]	[0.967]	[0.967]
P_{cc} [µÅ^2] [c]	39.76	40.81	39.75	39.76
σ [kHz] [d]	7.9	6.3	14.5	3.5
N[e]	23	31	11	21

Parameters	$^{13}C_6$	$^{18}O_7$	$^{2}H_8$
A [MHz]	3105.29183(97)	3144.82149(75)	3140.1882(10)
B [MHz]	2834.7260(12)	2745.4595(10)	2830.5029(10)
C [MHz]	1932.5314(11)	1905.2215(10)	1943.9372(11)
Δ_J [kHz]	0.417(24)	0.411(24)	0.489(27)
Δ_{JK} [kHz]	[2.740]	[2.740]	1.85(10)
Δ_K [kHz]	[-2.770]	[-2.770]	-1.949(83)
δ_J [kHz]	0.121(10)	0.0519(94)	0.255(13)
δ_K [kHz]	[0.967]	[0.967]	0.952(47)
P_{cc} [µÅ^2]	39.76	39.76	39.75
σ [kHz]	6.2	2.3	5.9
N	20	10	31

[a] Standard errors within parentheses are expressed in units of the last two digits. [b] Values in brackets fixed to the corresponding values of the parent species. [c] Planar moment along the molecule's c-axis, which is calculated using the formula, $P_{cc} = (I_a + I_b - I_c)/2$. [d] Root-mean-square deviation of the fit. [e] number of the lines in the fit.

Table S6. Spectroscopic parameters of the MOM-II$_a$ calculated at the B3LYP-D4/def2-QZVP and MP2/cc-pVQZ levels of theory, where the O···O distance is constrained at 2.933 Å, together with the

Parameters	B3LYP-D4	MP2	EXPT
A [MHz]	3158.7	3176.5	3144.9601(10)
B [MHz]	2882.3	2909.3	2874.01219(92)
C [MHz]	1973.4	1986.2	1966.32603(71)
$\|\mu_a\|$ [D]	2.5	2.5	+++[a]
$\|\mu_b\|$ [D]	1.3	1.4	++[a]
$\|\mu_c\|$ [D]	0.0	0.0	NO[a]

[a] Strength of the observed type of transitions: +++ (strong), ++ (medium), NO (not observed).

Table S7. Substitution coordinates (r_s) of heavy atoms (^{13}C and ^{18}O) and H_8 in the principal inertial axes systems of the parent species of MOM-I$_a$ and equilibrium coordinates (r_e) calculated at the B3LYP-D4/def2-QZVP and MP2/cc-pVQZ levels of theory, respectively.

Atoms	a/Å			b/Å			c/Å		
	r_e (B3LYP)	r_e (MP2)	r_s	r_e (B3LYP)	r_e (MP2)	r_s	r_e (B3LYP)	r_e (MP2)	r_s
C_1	0.005636	0.004754	0.00(1)	0.344406	0.374599	0.351(4)	0.075897	0.068760	0.051(29)
C_2	0.779572	0.772489	0.771(2)	-0.434503	-0.384970	-0.414(4)	-1.011429	-1.022181	-1.018(1)
C_3	1.321788	1.327798	1.325(1)	0.222153	0.264268	0.243(6)	0.873992	0.838419	0.859(2)
O_4	1.893365	1.839042	1.863(1)	-0.660844	-0.708325	-0.700(2)	-0.114112	-0.097698	-0.071(21)
C_5	-0.453786	-0.515662	-0.485(3)	1.747192	1.753616	1.754(1)	-0.281592	-0.258746	-0.263(6)
C_6	-1.103077	-1.047359	-1.075(1)	-0.481120	-0.502386	-0.491(3)	0.705229	0.704849	0.707(2)
O_7	-2.167849	-2.115746	-2.149(1)	-0.604651	-0.645804	-0.625(2)	-0.234700	-0.221305	-0.221(7)
H_8	-2.870577	-2.749451	-2.791(1)	-1.132087	-1.267237	-1.189(1)	0.150444	0.142180	0.138(11)

Table S8. Substitution coordinates (r_s) of heavy atoms (^{13}C and ^{18}O) in the principal inertial axes systems of the parent species of MOM-II$_a$ and equilibrium coordinates (r_e) optimized at the B3LYP-D4/def2-QZVP and MP2/cc-pVQZ levels of theory, respectively, while the $O_4 \cdots O_7$ distance is fixed at 2.933 Å.

	a/Å			b/Å			c/Å		
	r_e (B3LYP)	r_e (MP2)	r_s	r_e (B3LYP)	r_e (MP2)	r_s	r_e (B3LYP)	r_e (MP2)	r_s
C_1	-0.439364	-0.426544	-0.393(4)	-0.429422	-0.436018	-0.442(3)	0.000037	-0.000004	0.000(35)
C_2	-0.509619	-0.524383	-0.553(3)	0.718965	0.703730	0.680(2)	1.030670	1.024465	1.030(1)
C_3	-0.510446	-0.524947	-0.553(3)	0.719025	0.703780	0.680(2)	-1.030494	-1.024363	-1.030(1)
O_4	-0.187552	-0.224038	-0.300(5)	1.689479	1.687398	1.680(1)	-0.000015	-0.000025	0.000(19)
C_5	-1.560041	-1.518605	-1.457(1)	-1.451315	-1.478829	-1.550(1)	0.000224	0.000240	0.000(30)
C_6	0.926147	0.943604	0.995(2)	-1.112271	-1.089706	-1.051(1)	-0.000344	-0.000406	0.000(35)
O_7	2.035492	2.034553	2.046(1)	-0.223782	-0.183766	0.080(19)	0.000007	0.000149	0.000(53)

SUPPORTING INFORMATION

Figure S7. Potential energy curves obtained by rotating the hydroxyl group about the C_6-O_7 bond in the *cis*-$O_4C_1C_6O_7$ (b) conformations of the MOM monomer at various levels of theory, including (a). B3LYP-D4/def2-QZVP(B3D4) and CCSD(T)//B3LYP-D4, which is single-point energy calculations with the CCSD(T)/cc-pVTZ functional performed on the scanned geometries of the B3LYP-D4 PEC; (b). MP2/cc-pVQZ (mp2) and CCSD(T)//MP2, which is single-point energy calculation with the CCSD(T)/cc-pVTZ functional performed on the scanned geometries of the MP2 PEC; (c). M06-2X-D3/def2-QZVP (M06-2X-D3) and PW6B95-D4/ def2-QZVP (PW6B95-D4); (d) B2PLYP-D4/def2-QZVP (B2PLYP-D4) and PWPB95-D4/def2-QZVP (PWPB95-D4) levels of theory.

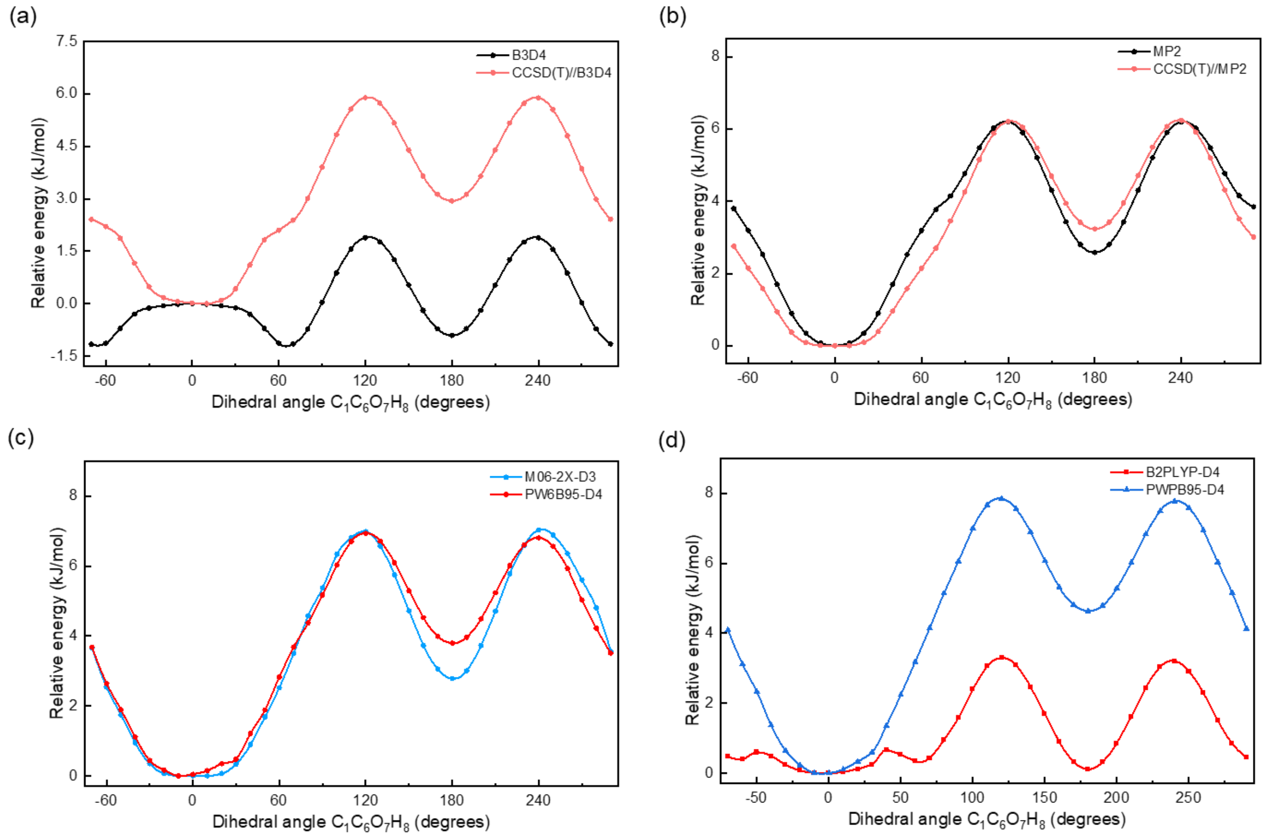

Figure S8. Relative energies (in kJ/mol) of the five conformers of the MOM monomer calculated at different levels of theory, including (a). B3LYP-D4/def2-QZVP (A1), CCSD(T)/cc-pVTZ//B3LYP-D4/def2-QZVP (A2), DLPNO-CCSD(T)/cc-pVTZ//B3LYP-D4/def2-QZVP (A3), and DLPNO-CCSD(T)/cc-pVQZ//B3LYP-D4/def2-QZVP (A4); (b). MP2/cc-pVQZ (B1), CCSD(T)/cc-pVTZ//MP2/cc-pVQZ (B2), DLPNO-CCSD(T)/cc-pVTZ// MP2/cc-pVQZ (B3), and DLPNO-CCSD(T)/cc-pVQZ// MP2/cc-pVQZ (B4); (c). M06-2X/def2-QZVP (C1), PW6B95-D4/def2-QZVP (C2), B2PLYP-D4/def2-QZVP (C3), and PWPB95-D4/def2-TZVP (C4). Note that the CCSD(T)//B3LYP-D4, DLPNO-CCSD(T)//B3LYP-D4, CCSD(T)//MP2, and DLPNO-CCSD(T)//MP2 functionals are used for performing single-point energy calculations with the CCSD(T) or DLPNO-CCSD(T) functional on the geometries optimized at the B3LYP-D4/def2-QZVP and MP2/cc-pVQZ levels of theory, respectively.

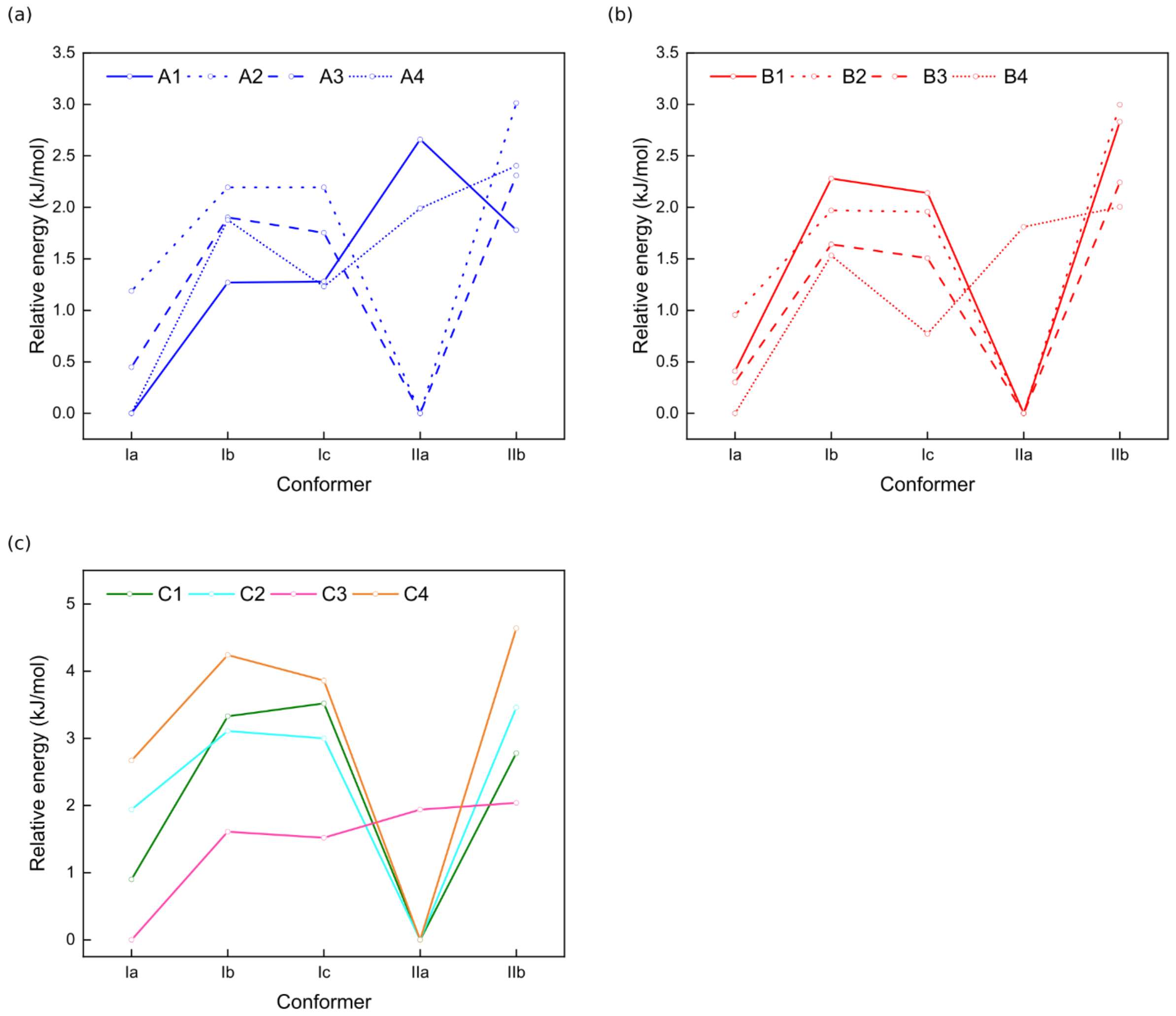

2.2. MOM monohydrate

Table S9. Cartesian coordinates (Å) for the equilibrium structure of *cis*MOM-1w$_a$ obtained at the B3LYP-D4/def2-QZVP level of theory.

Atom	a	b	c
C	-1.034185	0.033053	0.073960
C	-2.537530	-0.051062	0.281087
H	-2.825988	0.386965	1.238822
H	-2.880645	-1.085671	0.271205
H	-3.070426	0.489062	-0.503015
C	-0.567833	1.492830	0.178157
H	-1.251930	2.121429	-0.395024
H	-0.650104	1.801903	1.229086
O	0.715623	1.764281	-0.338329
H	1.404145	1.232885	0.093226
C	-0.461672	-0.727616	-1.141918
H	0.083288	-0.130801	-1.870274
H	-1.171357	-1.389815	-1.640815
O	0.442839	-1.490029	-0.290758
C	-0.190469	-0.957069	0.905202

H	0.541699	-0.531154	1.592121
H	-0.765944	-1.729439	1.419168
O	2.747820	-0.136228	0.336070
H	2.143030	-0.790227	-0.062042
H	3.502547	-0.066852	-0.252292

Table S10. Cartesian coordinates (Å) for the equilibrium structure of *g*MOM-1w$_b$ obtained at the B3LYP-D4/def2-QZVP level of theory.

Atom	a	b	c
C	0.734298	-0.392537	0.136540
C	2.130592	-0.989327	0.144084
H	2.789439	-0.436521	-0.523739
H	2.111453	-2.031901	-0.174431
H	2.562278	-0.949880	1.146147
C	0.726490	1.065374	0.569736
H	-0.299268	1.441007	0.582339
H	1.133463	1.142390	1.585857
O	1.526526	1.809261	-0.347964
H	1.344219	2.744103	-0.234443
C	-0.372646	-1.196241	0.850758
H	-0.868481	-0.714745	1.694812
H	-0.091013	-2.216183	1.119457
O	-1.207547	-1.182893	-0.338263
C	-0.116813	-0.627495	-1.129331
H	-0.426581	0.258463	-1.680915
H	0.282668	-1.375717	-1.815855
O	-2.795359	1.040558	0.157116
H	-2.434108	0.169459	-0.086061
H	-3.736137	0.997509	-0.023960

Figure S9. Part of the broadband spectrum of *cis*MOM-1w$_a$ collected with 2.4×10^6 FIDs. The upper trace is obtained from the experiment and the bottom trace is the simulation based on the fit.

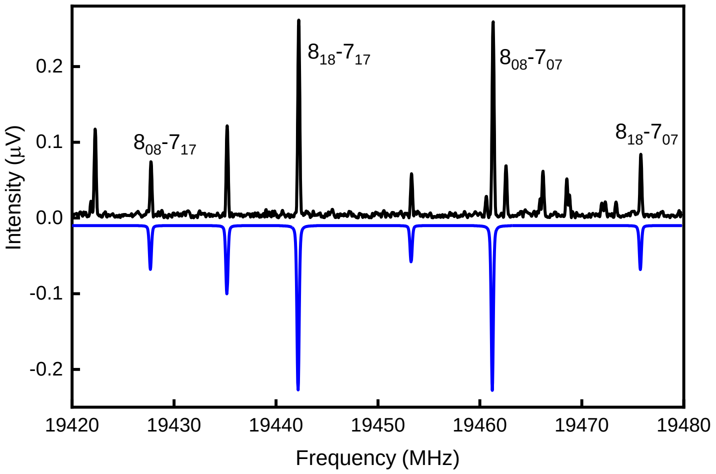

Table S11. Spectroscopic constants for *cis*MOM-1w$_a$ and *g*MOM-1w$_b$ obtained at the B3LYP-D4/def2-QZVP level of theory.

Parameters	*cis*MOM-1w$_a$	*g*MOM-1w$_b$
A [MHz]	2619.1	2368.0
B [MHz]	1556.4	1462.8
C [MHz]	1189.2	1089.2
$\|\mu_a\|$ [D]	1.6	0.5
$\|\mu_b\|$ [D]	1.0	0.3
$\|\mu_c\|$ [D]	0.2	0.6
ΔE [kJ/mol]	0.0	10.0
ΔE_{ZPE} [kJ/mol]	0.0	6.5

Table S12. Spectroscopic constants for *cis*MOM-1w$_a$ and *g*MOM-1w$_b$ obtained at the MP2/cc-pVQZ level of theory.

Parameters	*cis*MOM-1w$_a$	*g*MOM-1w$_b$
A [MHz]	2675.2	2373.9
B [MHz]	1559.9	1514.2
C [MHz]	1207.6	1117.6
$\|\mu_a\|$ [D]	1.6	0.4
$\|\mu_b\|$ [D]	0.9	0.3
$\|\mu_c\|$ [D]	0.1	0.5
ΔE [kJ/mol]	0.0	9.8

Table S13. Experimental spectroscopic constants of *cis*MOM-1w$_a$.

Parameters	*cis*MOM-1w$_a$
A [MHz]	2647.27498(85)[a]
B [MHz]	1513.39671(48)
C [MHz]	1171.12611(35)
Δ_J [kHz]	0.3731(32)
Δ_{JK} [kHz]	-0.595(17)
Δ_K [kHz]	1.655(31)
δ_J [kHz]	0.1102(13)
δ_K [kHz]	0.369(27)
a-type[b]	+++
b-type[b]	++
c-type[b]	NO
σ [kHz][c]	9.4
N[d]	86

[a] Standard errors within parentheses are expressed in units of the last two digits. [b] Strength of the observed type of transitions: +++ (strong), ++ (medium), NO (not observed). Root-mean-square deviation of the fit. [c] number of the lines in the fit.

2.3. MOM dihydrate

Table S14. Cartesian coordinates (Å) for the equilibrium structure of *cis*MOM-2w$_a$ obtained at the B3LYP-D4/def2-QZVP level of theory.

Atom	a	b	c
C	1.427254	-0.016503	-0.102464
C	2.880900	0.110747	-0.529573
H	3.124428	1.137941	-0.801055
H	3.553296	-0.191761	0.275196
H	3.091890	-0.522508	-1.393705
C	1.090783	-1.475823	0.225438
H	1.848630	-1.860909	0.912290
O	-0.151586	-1.660453	0.861195
H	-0.888215	-1.600097	0.227122

H	1.162234	-2.066332	-0.696661
C	0.409616	0.684874	-1.028188
H	-0.408519	0.072827	-1.405950
H	0.847316	1.259523	-1.846298
O	-0.031030	1.562320	0.045429
C	0.949930	0.995092	0.962177
H	0.466864	0.562362	1.835407
H	1.684098	1.746347	1.255845
O	-2.419265	-1.297584	-0.748434
H	-2.678974	-0.419396	-0.397002
H	-3.106538	-1.907924	-0.472500
O	-2.706170	1.198246	0.337826
H	-1.752545	1.434807	0.321339
H	-3.173428	1.936289	-0.058872

Table S15. Cartesian coordinates (Å) for the equilibrium structure of *g*MOM-2w$_b$ obtained at the B3LYP-D4/def2-QZVP level of theory.

Atom	a	b	c
C	1.285178	0.001853	0.048605
C	2.657539	0.531998	-0.318362
H	2.564967	1.477972	-0.851136
H	3.254687	0.712112	0.577529
H	3.201511	-0.169405	-0.951950
C	0.489915	1.009819	0.887595
H	1.108592	1.326772	1.732418
O	0.126693	2.156744	0.149392
H	-0.789480	2.028903	-0.146068
H	-0.398160	0.523088	1.301290
C	1.191946	-1.414679	0.653233
H	0.853598	-1.500464	1.686588
H	2.078434	-2.033529	0.501404
O	0.145724	-1.783536	-0.291517
C	0.437342	-0.593919	-1.090926
H	-0.466060	-0.062701	-1.381813
H	1.010192	-0.867598	-1.978588
O	-2.506622	1.299841	-0.455548
H	-3.225672	1.809670	-0.076596
H	-2.592309	0.393897	-0.095082
O	-2.442820	-1.326272	0.425356
H	-3.024050	-1.988213	0.045251
H	-1.526685	-1.618317	0.226575

Table S16. Cartesian coordinates (Å) for the equilibrium structure of *cis*MOM-2w$_c$ obtained at the B3LYP-D4/def2-QZVP level of theory.

Atom	a	b	c
C	-1.376713	0.287251	0.031861
C	-2.806125	0.735628	0.296023
H	-3.399570	-0.077228	0.714114
H	-3.292229	1.064888	-0.623992
H	-2.830311	1.567956	1.001599
C	-0.593259	1.423596	-0.628747
H	-1.017461	1.598605	-1.624848
O	0.807465	1.235540	-0.719111
H	1.049276	0.347364	-1.048496
H	-0.734014	2.339431	-0.052442
C	-0.665166	-0.410948	1.213466
H	0.288997	0.009488	1.526136
H	-1.296631	-0.590177	2.085210
O	-0.484413	-1.629852	0.441432
C	-1.237499	-1.082605	-0.670637
H	-0.659108	-1.104513	-1.596219
H	-2.175004	-1.621387	-0.818484
O	2.964244	0.730743	0.947607
H	2.246453	1.197918	0.484912
H	3.736974	1.297051	0.894482
O	1.985682	-1.271205	-0.875244
H	2.605648	-0.856323	-0.255942
H	1.313878	-1.683811	-0.308576

Figure S10. Part of the broadband spectrum of *cis*MOM-2w$_a$ and *g*MOM-2w$_b$ collected with 2.4×10^6 FIDs.

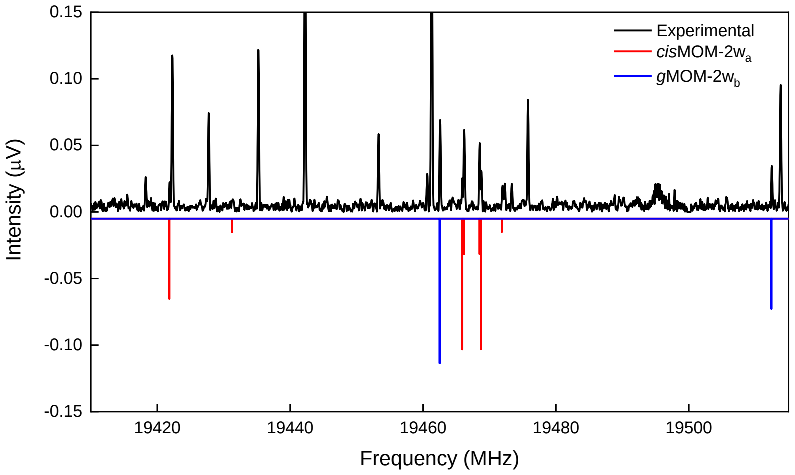

Table S17. Spectroscopic constants for *cis*MOM-2w$_a$, *g*MOM-2w$_b$, and *cis*MOM-2w$_c$ obtained at the B3LYP-D4/def2-QZVP level of theory.

Parameters	*cis*MOM-2w$_a$	*g*MOM-2w$_b$	*cis*MOM-2w$_c$
A [MHz]	1878.3	1660.4	1975.3
B [MHz]	1011.1	1143.4	981.3
C [MHz]	792.4	791.2	837.7
$\|\mu_a\|$ [D]	0.8	0.4	2.5
$\|\mu_b\|$ [D]	1.1	2.1	2.2
$\|\mu_c\|$ [D]	1.3	0.4	0.5
ΔE [kJ/mol]	0.0	2.6	9.3
ΔE_{ZPE} [kJ/mol]	0.0	2.7	10.3

Table S18. Spectroscopic constants for *cis*MOM-2w$_a$, *g*MOM-2w$_b$, and *cis*MOM-2w$_c$ obtained at the MP2/cc-pVQZ level of theory.

Parameters	*cis*MOM-2w$_a$	*g*MOM-2w$_b$	*cis*MOM-2w$_c$
A [MHz]	1911.4	1681.6	1990.8
B [MHz]	1018.4	1161.6	998.3
C [MHz]	808.8	809.2	854.0
$\|\mu_a\|$ [D]	0.8	0.5	2.4
$\|\mu_b\|$ [D]	0.9	2.1	2.2
$\|\mu_c\|$ [D]	1.4	0.3	0.5
ΔE [kJ/mol]	0.0	0.0	7.0

Table S19. Experimental spectroscopic constants of *cis*MOM-2w$_a$ and *g*MOM-2w$_b$.

Parameters	*cis*MOM-2w$_a$	*g*MOM-2w$_b$
A [MHz]	1865.31011(48)[a]	1641.09353(38)
B [MHz]	993.95876(30)	1131.82654(34)
C [MHz]	782.85788(33)	779.36667(22)

Δ_J [kHz]	0.1806(39)	0.2090(23)
Δ_{JK} [kHz]	0.982(17)	0.6312(88)
Δ_K [kHz]	-0.317(14)	-0.4917(74)
δ_J [kHz]	0.0042(28)	0.0403(12)
δ_K [kHz]	0.737(28)	0.5483(74)
a-type[b]	++	+
b-type[b]	++	+++
c-type[b]	+++	NO
σ [kHz] [c]	7.1	7.5
N[d]	90	96

[a] Standard errors within parentheses are expressed in units of the last two digits.
[b] Strength of the observed type of transitions: +++ (strong), ++ (medium), + (weak), NO (not observed). [c] Root-mean-square deviation of the fit.
[d] number of the lines in the fit.

2.4. MOM trihydrate

Table S20. Cartesian coordinates (Å) for the equilibrium structure of *cis*MOM-3w$_a$ obtained at the B3LYP-D4/def2-QZVP level of theory.

Atom	a	b	c
C	-1.650175	-0.112113	0.016659
C	-3.168578	-0.211185	0.024341
H	-3.620533	0.708487	-0.345962
H	-3.511851	-1.032165	-0.607698
H	-3.546592	-0.387407	1.033191
C	-1.049351	-1.410687	0.559328
H	-1.438769	-2.248324	-0.022653
O	0.363105	-1.499636	0.487875
H	0.799699	-0.959067	1.178656
H	-1.382899	-1.550478	1.593057
C	-1.071362	1.184774	0.626902
H	-0.346057	1.071825	1.430900
H	-1.819685	1.927711	0.909234
O	-0.431418	1.541908	-0.627627
C	-1.023373	0.397945	-1.300616
H	-0.263971	-0.231581	-1.760127
H	-1.744420	0.718911	-2.054627
O	2.363518	1.290896	-0.338755
H	2.505458	0.494258	-0.878479
H	1.445750	1.550960	-0.539077
O	2.123426	-1.245715	-1.591793
H	2.738797	-1.978764	-1.653403
H	1.472620	-1.484108	-0.900800
O	1.806946	0.202121	2.049835
H	2.567372	-0.058582	2.572980
H	2.152818	0.663675	1.243392

Table S21. Cartesian coordinates (Å) for the equilibrium structure of *cis*MOM-3w$_b$ obtained at the B3LYP-D4/def2-QZVP level of theory.

Atom	a	b	c
C	1.721985	0.269660	0.111939
C	3.068535	0.784017	0.597264
H	3.711661	1.053395	-0.242746
H	2.947671	1.670941	1.221668
H	3.587275	0.027538	1.185715
C	1.027773	1.336456	-0.735006
H	1.655427	1.552485	-1.604114
O	-0.253926	0.956469	-1.210351

H	-0.936032	1.365657	-0.639543
H	0.949295	2.258533	-0.151734
C	0.835955	-0.390824	1.191735
H	-0.177707	-0.005036	1.291445
H	1.295322	-0.476171	2.177710
O	0.856698	-1.665840	0.493420
C	1.744258	-1.136411	-0.526236
H	1.304004	-1.216572	-1.520486
H	2.711564	-1.641311	-0.508229
O	-3.360929	-0.502040	0.678564
H	-4.262018	-0.790473	0.523738
H	-2.785020	-0.997744	0.046725
O	-1.530567	-1.596325	-0.967410
H	-0.827840	-1.929142	-0.382525
H	-1.138017	-0.773193	-1.304473
O	-2.172435	1.918537	0.471065
H	-2.756746	2.615702	0.166368
H	-2.735125	1.123234	0.610002

Table S22. Cartesian coordinates (Å) for the equilibrium structure of *cis*MOM-3w$_c$ obtained at the B3LYP-D4/def2-QZVP level of theory.

Atom	a	b	c
C	-1.80593	0.03804	-0.05934
C	-3.25046	-0.03799	-0.52959
H	-3.94103	0.13536	0.29781
H	-3.45204	0.71405	-1.29483
H	-3.47632	-1.01632	-0.95413
C	-1.50674	1.41728	0.5331
H	-2.23374	1.61678	1.32511
O	-0.22541	1.52913	1.10736
H	0.42289	1.77642	0.42424
H	-1.6634	2.17568	-0.24361
C	-0.77305	-0.48459	-1.08187
H	0.09588	0.14988	-1.2452
H	-1.18051	-0.81464	-2.03833
O	-0.44849	-1.62407	-0.23357
C	-1.34545	-1.15017	0.81177
H	-0.79061	-0.87286	1.70608
H	-2.10567	-1.8971	1.04239
O	3.54665	0.25799	0.07223
H	3.08897	-0.6122	0.09773
H	3.82538	0.42449	0.9756
O	2.16907	-2.072	0.24759
H	2.42452	-2.87991	-0.20163
H	1.2051	-1.95458	0.08458
O	1.6790	1.99636	-0.8446
H	2.06643	2.84168	-1.07823
H	2.42029	1.41511	-0.55594

Figure S11. Part of the broadband spectrum of *cis*MOM-3w$_a$ and *cis*MOM-3w$_b$ collected with 3.1×10^6 FIDs.

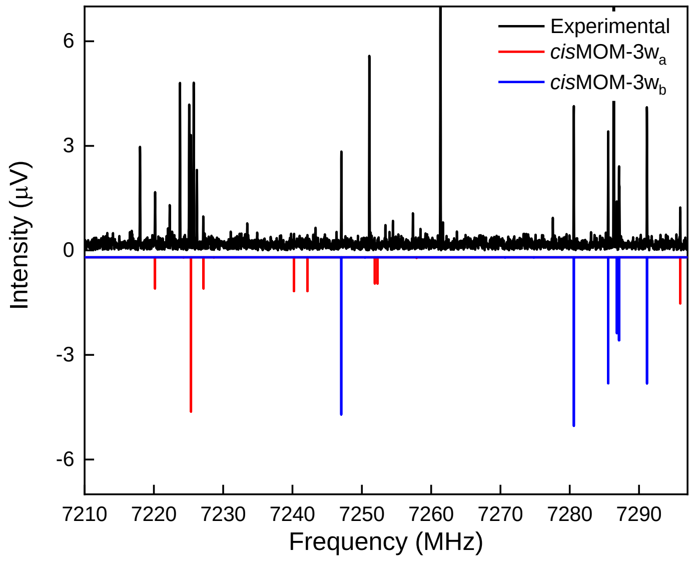

Table S23. Spectroscopic constants for *cis*MOM-3w$_a$, *cis*MOM-3w$_b$, and *cis*MOM-3w$_c$ obtained at the B3LYP-D4/def2-QZVP level of theory.

Parameters	*cis*MOM-3w$_a$	*cis*MOM-3w$_b$	*cis*MOM-3w$_c$
A [MHz]	1359.8	1470.2	1348.4
B [MHz]	757.4	712.8	699.3
C [MHz]	736.1	595.9	537.6
$\|\mu_a\|$ [D]	1.9	0.8	1.1
$\|\mu_b\|$ [D]	2.5	2.1	0.3
$\|\mu_c\|$ [D]	0.9	0.2	0.9
ΔE [kJ/mol]	0.0	3.4	9.6
ΔE_{ZPE} [kJ/mol]	0.0	2.6	6.4

Table S24. Spectroscopic constants for *cis*MOM-3w$_a$, *cis*MOM-3w$_b$, and *cis*MOM-3w$_c$ obtained at the MP2/cc-pVQZ level of theory.

Parameters	*cis*MOM-3w$_a$	*cis*MOM-3w$_b$	*cis*MOM-3w$_c$
A [MHz]	1368.9	1471.9	1345.8
B [MHz]	763.6	730.1	716.3
C [MHz]	747.9	610.8	548.2
$\|\mu_a\|$ [D]	1.9	0.6	1.1
$\|\mu_b\|$ [D]	2.5	2.2	0.0
$\|\mu_c\|$ [D]	1.1	0.3	0.9
ΔE [kJ/mol]	0.0	3.2	11.2

Table S25. Experimental spectroscopic constants of *cis*MOM-3w$_a$ and *cis*MOM-3w$_b$.

Parameters	*cis*MOM-3w$_a$	*cis*MOM-3w$_b$
A [MHz]	1342.97316(39)	1451.59571(26)

B [MHz]	736.20819(55)	700.09120(26)
C [MHz]	721.03640(58)	585.82918(25)
Δ_J [kHz]	0.0968(27)	0.2991(23)
Δ_{JK} [kHz]	0.0892(85)	-0.8618(99)
Δ_K [kHz]	0.2742(73)	2.3057(86)
δ_J [kHz]	0.0179(76)	0.0458(20)
δ_K [kHz]	⋯	0.435(35)
a-type[b]	+++	++
b-type[b]	+++	+++
c-type[b]	+	NO
σ [kHz][c]	6.9	5.2
N[d]	59	87

[a] Standard errors within parentheses are expressed in units of the last two digits.
[b] Strength of the observed type of transitions: +++ (strong), ++ (medium),
+ (weak), NO (not observed). [c] Root-mean-square deviation of the fit.
[d] number of the lines in the fit.

2.5. MOM tetrahydrate

Table S26 Cartesian coordinates (Å) for the equilibrium structure of *cis*MOM-4w$_a$ obtained at the B3LYP-D4/def2-QZVP level of theory.

Atom	a	b	c
C	1.90962	0.17468	0.12886
C	3.37094	0.48862	0.41357
H	3.51567	1.55556	0.59272
H	3.72154	-0.04919	1.29407
H	4.00512	0.20301	-0.42783
C	1.44979	0.92865	-1.11856
H	2.06233	0.61158	-1.96583
O	0.09651	0.70304	-1.47374
H	-0.48747	1.3373	-1.00368
H	1.62191	1.99966	-0.97474
C	0.96995	0.27716	1.35123
H	0.07379	0.88099	1.2224
H	1.45909	0.54033	2.29051
O	0.67033	-1.14278	1.29728
C	1.54839	-1.32749	0.15436
H	1.00384	-1.70785	-0.70857
H	2.37263	-1.99992	0.39734
O	-3.18356	0.69229	1.062
H	-2.79876	-0.21318	0.91814
H	-3.475	0.72372	1.97499
O	-2.00492	-1.65779	0.63536
H	-1.09231	-1.57653	0.96862
H	-1.89678	-1.76733	-0.32671
O	-1.49732	2.40437	-0.10172
H	-1.99641	3.05386	-0.60124
H	-2.1628	1.84659	0.37212
O	-1.23456	-1.60495	-2.06668
H	-1.78532	-1.50291	-2.84514
H	-0.73197	-0.77008	-1.96784

Table S27. Cartesian coordinates (Å) for the equilibrium structure of *cis*MOM-4w$_b$ obtained at the B3LYP-D4/def2-QZVP level of theory.

Atom	a	b	c
C	-1.9526	-0.2001	-0.15174

C	-3.42421	-0.49423	-0.40076
H	-3.79702	0.07142	-1.25433
H	-3.58326	-1.55425	-0.60599
H	-4.03251	-0.22915	0.46645
C	-1.47489	-0.97183	1.07993
H	-1.73189	-2.02583	0.95718
O	-0.07919	-0.92818	1.31278
H	0.21629	-0.06097	1.67099
H	-2.01649	-0.60584	1.95874
C	-1.57151	1.29751	-0.17828
H	-1.04415	1.68645	0.69161
H	-2.38729	1.97177	-0.44442
O	-0.67391	1.09433	-1.30224
C	-1.035	-0.3093	-1.39044
H	-0.16418	-0.9546	-1.29322
H	-1.55323	-0.52079	-2.32752
O	0.92338	1.44826	2.07621
H	1.39921	1.64511	1.2251
H	1.55914	1.55116	2.78667
O	1.76012	-2.33256	-0.06482
H	1.09178	-1.85697	0.47882
H	2.05982	-3.07441	0.46427
O	3.50659	-0.3936	-0.92556
H	3.75805	-0.57208	-1.83384
H	2.95502	-1.15134	-0.63952
O	1.89085	1.80715	-0.36717
H	1.07389	1.61829	-0.86182
H	2.51571	1.09774	-0.61357

Table S28. Cartesian coordinates (Å) for the equilibrium structure of *cis*MOM-4w$_c$ obtained at the B3LYP-D4/def2-QZVP level of theory.

Atom	a	b	c
C	-2.09456	0.44219	0.0156
C	-3.5352	0.92299	-0.04849
H	-4.22788	0.08467	-0.12218
H	-3.79605	1.4975	0.8418
H	-3.69481	1.56624	-0.91595
C	-1.13961	1.63427	0.1112
H	-1.43042	2.24976	0.96532
O	0.21184	1.27883	0.30424
H	0.63962	0.98239	-0.53151
H	-1.25183	2.25326	-0.78638
C	-1.69801	-0.60799	-1.04417
H	-0.83556	-0.37019	-1.66543
H	-2.51657	-0.96783	-1.66883
O	-1.3654	-1.58416	-0.01619
C	-1.80471	-0.6792	1.03764
H	-1.00412	-0.48392	1.74891
H	-2.67982	-1.07923	1.55144
O	3.92973	0.87727	-0.20067
H	4.27586	1.75019	-0.00558
H	3.45224	0.58255	0.60453
O	2.20581	-0.10562	1.7251
H	1.46094	0.46555	1.47463
H	2.01138	-0.9375	1.26687
O	1.68043	0.23718	-1.71505
H	2.54101	0.5642	-1.38796
H	1.65747	-0.68454	-1.41661
O	1.27301	-2.13593	-0.13085
H	1.46977	-3.06908	-0.23745
H	0.296	-2.0578	-0.03999

Figure S12. Part of the broadband spectrum of *cis*MOM-4w$_a$ collected with 0.5×10^6 FIDs.

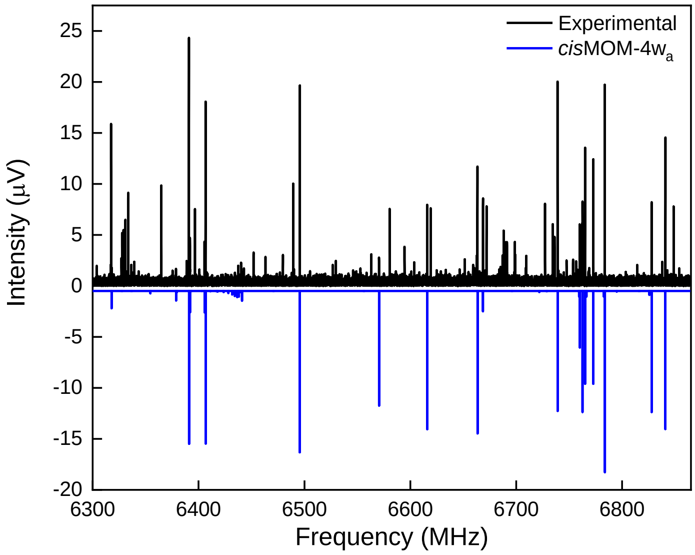

Table S29. Spectroscopic constants for *cis*MOM-4w$_a$, *cis*MOM-4w$_b$, and *cis*MOM-4w$_c$ obtained at the B3LYP-D4/def2-QZVP level of theory.

Parameters	*cis*MOM-4w$_a$	*cis*MOM-4w$_b$	*cis*MOM-4w$_c$
A [MHz]	978.2	1021.7	1293.4
B [MHz]	585.1	566.6	516.6
C [MHz]	546.8	524.7	469.2
$\|\mu_a\|$ [D]	1.9	2.3	3.9
$\|\mu_b\|$ [D]	2.7	2.8	0.3
$\|\mu_c\|$ [D]	0.7	0.6	0.1
ΔE [kJ/mol]	0.0	2.4	3.8
ΔE_{ZPE} [kJ/mol]	0.0	2.5	5.4

Table S30. Spectroscopic constants for *cis*MOM-4w$_a$, *cis*MOM-4w$_b$, and *cis*MOM-4w$_c$ obtained at the MP2/cc-pVQZ level of theory.

Parameters	*cis*MOM-4w$_a$	*cis*MOM-4w$_b$	*cis*MOM-4w$_c$
A [MHz]	977.4	1020.7	1312.3
B [MHz]	595.0	575.0	516.4
C [MHz]	555.5	531.6	469.6
$\|\mu_a\|$ [D]	1.8	2.2	3.9
$\|\mu_b\|$ [D]	2.8	2.8	0.2
$\|\mu_c\|$ [D]	0.9	0.8	0.0
ΔE [kJ/mol]	0.0	3.1	2.0

Table S31. Experimental spectroscopic constants of *cis*MOM-4w$_a$.

Parameters	*cis*MOM-4w$_a$
A [MHz]	942.04666(15)[a]
B [MHz]	582.10462(17)

C [MHz]	543.30530(15)
Δ_J [kHz]	0.1348(15)
Δ_{JK} [kHz]	-0.3118(66)
Δ_K[kHz]	0.6228(52)
δ_J [kHz]	0.0169(10)
δ_K [kHz]	-0.128(30)
a-type[b]	+++
b-type[b]	+++
c-type[b]	+
σ [kHz][c]	3.6
N[d]	97

[a] Standard errors within parentheses are expressed in units of the last two digits. [b] Strength of the observed type of transitions: +++ (strong), ++ (medium), + (weak), NO (not observed). [c] Root-mean-square deviation of the fit. [d] number of the lines in the fit.

Table S32. Spectroscopic constants for *cis*MOM-4w$_d$ – *cis*MOM-4w$_h$ obtained at the B3LYP-D4/def2-QZVP level of theory.

Parameters	*cis*MOM-4w$_d$	*cis*MOM-4w$_e$	*cis*MOM-4w$_f$	*cis*MOM-4w$_g$	*cis*MOM-4w$_h$
A [MHz]	976.3	1109.2	1109.0	1083.4	1125.0
B [MHz]	613.8	570.7	555.6	592.9	485.4
C [MHz]	517.1	506.0	495.3	520.4	403.6
$\lvert\mu_a\rvert$ [D]	3.3	4.3	2.7	3.5	1.4
$\lvert\mu_b\rvert$ [D]	1.1	0.4	0.8	2.9	2.1
$\lvert\mu_c\rvert$ [D]	1.0	1.8	1.0	2.4	0.6
ΔE [kJ/mol] [a]	4.8	6.4	6.8	7.0	7.5

[a] The relative energy is relative to the potential energy of MOM-4w$_a$.

Figure S13. Five high-energy isomers of the MOM tetrahydrate within an energy window of 8 kJ/mol predicted at the B3LYP-D4/def2-QZVP level of theory. The relative energies (in kJ/mol) to *cis*MOM-4w$_a$ are given in the parentheses.

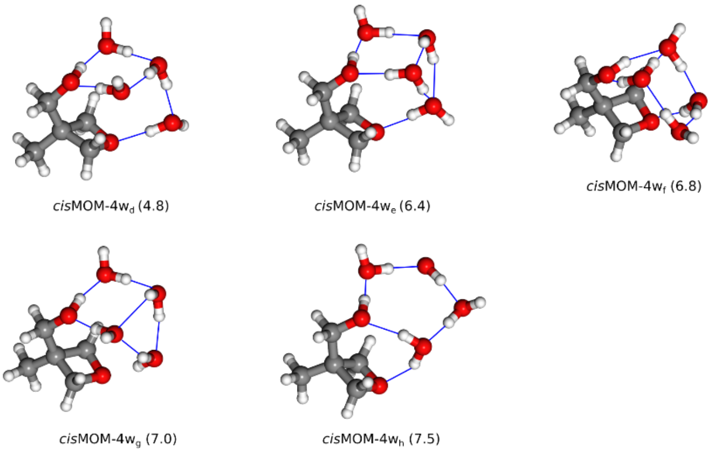

2.6. MOM pentahydrate

Figure S14. Nine isomers of the MOM pentahydrate within an energy window of 6 kJ/mol predicted at the B3LYP-D4/def2-QZVP level of theory. The relative energies (in kJ/mol) are given in the parentheses.

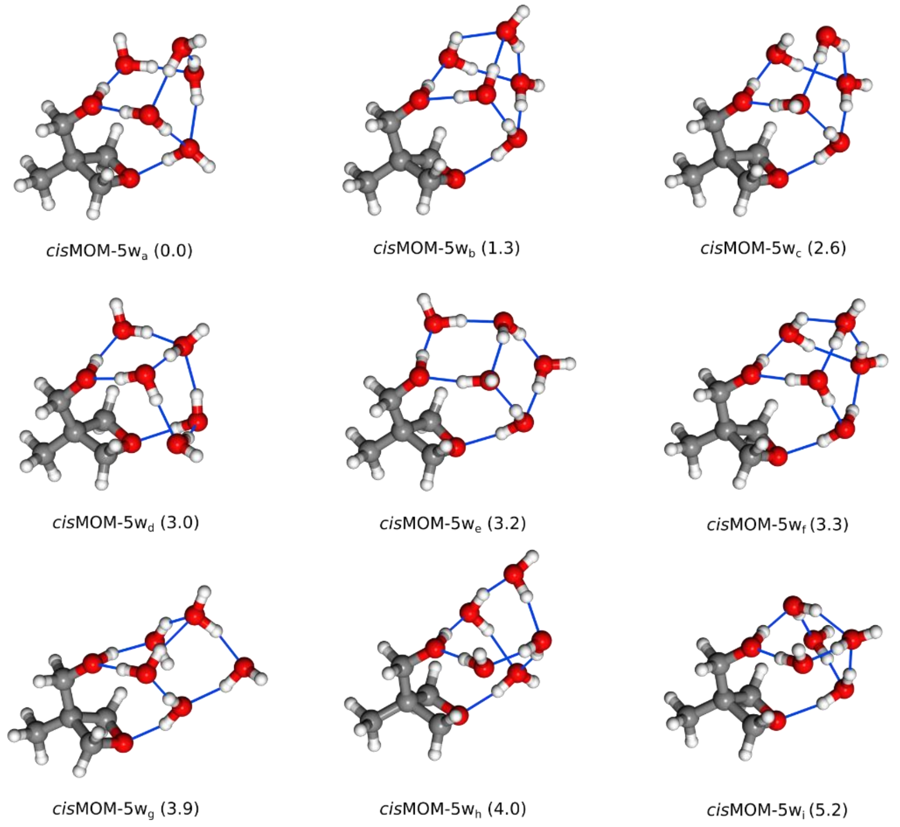

Table S33. Spectroscopic constants for *cis*MOM-5w$_a$ – *cis*MOM-5w$_i$ obtained at the B3LYP-D4/def2-QZVP level of theory.

Parameters	*cis*MOM-5w$_a$	*cis*MOM-5w$_b$	*cis*MOM-5w$_c$	*cis*MOM-5w$_d$	*cis*MOM-5w$_e$	*cis*MOM-5w$_f$	*cis*MOM-5w$_g$	*cis*MOM-5w$_h$	*cis*MOM-5w$_i$
A [MHz]	979.4	1002.2	953.7	793.5	853.1	987.2	1190.9	954.6	767.1
B [MHz]	417.5	425.3	442.0	500.7	459.8	426.1	380.6	421.6	516.6
C [MHz]	397.4	394.3	410.6	428.6	387.5	391.5	347.9	403.3	429.1
$\|\mu_a\|$ [D]	3.2	0.1	4.2	0.9	3.2	0.0	0.8	3.3	0.8
$\|\mu_b\|$ [D]	1.0	0.9	0.5	2.1	1.8	1.3	0.6	0.9	2.2
$\|\mu_c\|$ [D]	0.0	2.3	0.9	0.1	1.8	2.1	0.2	0.0	0.4
ΔE [kJ/mol]	0.0	1.3	2.6	3.0	3.2	3.3	3.9	4.0	5.2
ΔE_{ZPE} [kJ/mol]	0.0	3.4	4.3	…	…	…	…	…	…

Table S34. Cartesian coordinates (Å) for the equilibrium structure of *cis*MOM-5w$_a$ obtained at the B3LYP-D4/def2-QZVP level of theory.

Atom	a	b	c
C	-2.37106	-0.2509	-0.14525
C	-3.86338	-0.54194	-0.15466
H	-4.42934	0.28599	0.27616
H	-4.09103	-1.44071	0.42419
H	-4.22581	-0.7022	-1.17341
C	-1.59692	-1.41569	-0.7647
O	-0.20198	-1.20316	-0.83157
H	0.22985	-1.50839	0.00221
H	-1.81685	-2.33022	-0.20116

H	-1.95369	-1.571	-1.78764
C	-1.80512	0.25736	1.19947
H	-2.55033	0.42415	1.98046
H	-0.95235	-0.28501	1.60663
O	-1.40145	1.52406	0.59739
C	-1.96211	1.13455	-0.68921
H	-1.20161	1.13681	-1.47084
H	-2.78915	1.79184	-0.96737
O	1.65604	0.57711	-1.91296
H	0.96027	-0.07439	-1.69785
H	1.5477	1.27304	-1.24182
O	1.1955	2.17353	0.44161
H	1.27645	3.11941	0.59812
H	0.24119	1.94621	0.57423
O	2.79849	0.06604	1.54257
H	3.35292	-0.13417	0.75778
H	2.3302	0.88778	1.31333
O	0.9477	-1.8461	1.51036
H	1.6647	-1.15681	1.60237
H	1.38289	-2.69863	1.60858
O	3.90643	-0.58329	-0.89812
H	4.71231	-0.28131	-1.32699
H	3.15291	-0.1985	-1.40696

Table S35. Cartesian coordinates (Å) for the equilibrium structure of *cis*MOM-5w$_b$ obtained at the B3LYP-D4/def2-QZVP level of theory.

Atom	a	b	c
C	-2.39265	-0.28765	0.07009
C	-3.86412	-0.56002	0.34204
H	-4.38932	0.35644	0.61683
H	-3.98595	-1.27506	1.15986
H	-4.35545	-0.97662	-0.54108
C	-1.67777	-1.57857	-0.32719
O	-0.31065	-1.4005	-0.64015
H	0.24469	-1.47518	0.16834
H	-1.79048	-2.31595	0.47652
H	-2.16334	-1.99113	-1.21685
C	-1.67584	0.55332	1.149
H	-2.29559	0.86609	1.9928
H	-0.72994	0.15251	1.51756
O	-1.46311	1.64863	0.22031
C	-2.10897	0.91551	-0.85586
H	-1.41795	0.72504	-1.67843
H	-2.98879	1.44913	-1.22271
O	1.58442	0.05669	-2.11216
H	0.85564	-0.51095	-1.79992
H	2.38586	-0.34934	-1.73977
O	1.22658	2.13677	-0.3385
H	0.27543	2.09141	-0.1145
H	1.3462	1.50864	-1.08883
O	3.6901	-0.91636	-0.35825
H	3.59374	-0.16683	0.26702
H	4.62626	-0.99714	-0.56444
O	1.45673	-1.58429	1.41913
H	1.77897	-0.69794	1.6713
H	2.20102	-1.94399	0.91379
O	2.70729	0.93664	1.41993
H	2.14086	1.49469	0.79492
H	3.09618	1.52809	2.07063

Table S36. Cartesian coordinates (Å) for the equilibrium structure of *cis*MOM-5w$_c$ obtained at the B3LYP-D4/def2-QZVP level of theory.

Atom	a	b	c
C	-2.31841	-0.30596	0.11219
C	-3.76992	-0.60107	0.45817
H	-4.31271	0.31675	0.69068
H	-3.83997	-1.26158	1.32612
H	-4.28059	-1.09063	-0.37524
C	-1.58129	-1.60068	-0.22507
O	-0.22088	-1.41582	-0.56562
H	0.35859	-1.48051	0.23998
H	-1.66045	-2.29324	0.62029

H	-2.07479	-2.07522	-1.07927
C	-1.59134	0.62997	1.10264
H	-2.18618	0.97386	1.95167
H	-0.61364	0.29321	1.44959
O	-1.46479	1.67025	0.09554
C	-2.11268	0.83938	-0.90274
H	-1.43958	0.61832	-1.73368
H	-3.02327	1.31106	-1.27942
O	1.50152	-0.09999	-2.13322
H	0.77076	-0.61453	-1.71427
H	1.49991	-0.29492	-3.07511
O	1.21067	2.18197	-0.46647
H	0.25231	2.13952	-0.27359
H	1.34788	1.56915	-1.21024
O	3.61866	-0.96572	-0.33845
H	3.69225	-0.19826	0.25049
H	3.03443	-0.659	-1.05344
O	1.5223	-1.54559	1.43327
H	1.72528	-0.65449	1.77277
H	2.30593	-1.71312	0.87013
O	2.57972	1.06883	1.54342
H	2.10419	1.56896	0.82798
H	2.93917	1.72097	2.15179

Figure S15. Part of the broadband spectrum of *cis*MOM-5w$_a$ collected with 0.5×10^6 FIDs.

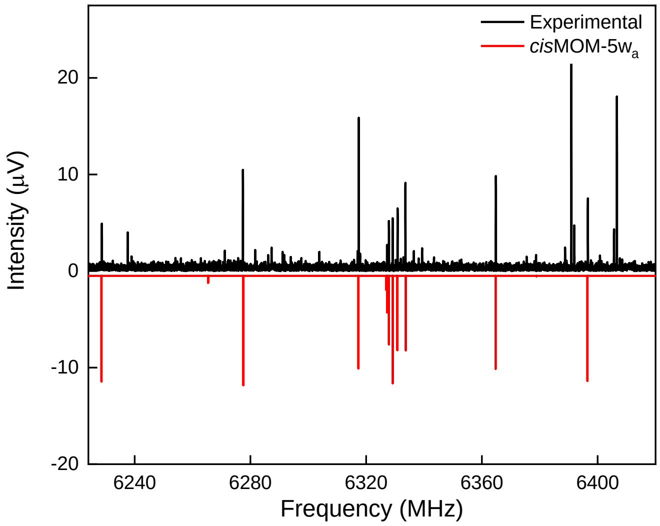

Table S37. Spectroscopic constants for *cis*MOM-5w$_a$, *cis*MOM-5w$_b$, and *cis*MOM-5w$_c$ obtained at the MP2/cc-pVQZ level of theory.

Parameters	*cis*MOM-5w$_a$	*cis*MOM-5w$_b$	*cis*MOM-5w$_c$
A [MHz]	984.0	1003.3	954.2
B [MHz]	419.3	430.0	445.1
C [MHz]	398.8	398.3	416.8
$\|\mu_a\|$ [D]	3.2	0.2	4.0
$\|\mu_b\|$ [D]	0.8	1.0	0.2
$\|\mu_c\|$ [D]	0.0	2.3	0.9
ΔE [kJ/mol]	0.0	1.8	2.5

Table S38. Experimental spectroscopic constants of *cis*MOM-5w$_a$.

Parameters	*cis*MOM-5w$_a$
A [MHz]	955.924(22)[a]

B [MHz]	405.97907(36)
C [MHz]	384.64133(38)
Δ_J [kHz]	0.05664(75)
Δ_{JK} [kHz]	-0.1174(43)
δ_J [kHz]	0.0082(11)
a-type[b]	+++
b-type[b]	NO
c-type[b]	NO
σ [kHz][c]	3.5
N[d]	47

[a] Standard errors within parentheses are expressed in units of the last two digits. [b] Strength of the observed type of transitions: +++ (strong), NO (not observed). [c] Root-mean-square deviation of the fit. [d] number of the lines in the fit.

2.7. MOM hexahydrate

Table S39. Spectroscopic constants for *cis*MOM-6w$_a$ – *cis*MOM-6w$_i$ obtained at the B3LYP-D4/def2-QZVP level of theory.

Parameters	*cis*MOM-6w$_a$	*cis*MOM-6w$_b$	*cis*MOM-6w$_c$	*cis*MOM-6w$_d$	*cis*MOM-6w$_e$	*cis*MOM-6w$_f$	*cis*MOM-6w$_g$	*cis*MOM-6w$_h$	*cis*MOM-6w$_i$
A [MHz]	698.6	706.9	886.8	898.0	719.6	875.1	879.3	782.9	784.3
B [MHz]	384.7	387.9	327.0	323.9	383.4	331.2	329.8	359.7	345.7
C [MHz]	368.0	358.6	306.1	299.3	353.6	307.6	305.6	313.1	308.7
$\lvert\mu_a\rvert$ [D]	2.2	2.8	1.9	0.9	2.7	1.2	2.0	0.2	2.9
$\lvert\mu_b\rvert$ [D]	1.4	1.7	1.0	0.2	1.7	0.1	0.8	0.9	0.3
$\lvert\mu_c\rvert$ [D]	0.8	0.5	1.1	1.6	0.6	1.5	1.4	0.4	0.7
ΔE [kJ/mol]	0.0	4.6	5.6	5.6	5.7	6.7	7.1	7.1	7.4
ΔE_{ZPE} [kJ/mol]	0.0	5.1	6.3	…	…	…	…	…	…

Figure S16. Nine isomers of the MOM hexahydrate within an energy window of 8 kJ/mol predicted at the B3LYP-D4/def2-QZVP level of theory. The relative energies (in kJ/mol) are given in the parentheses.

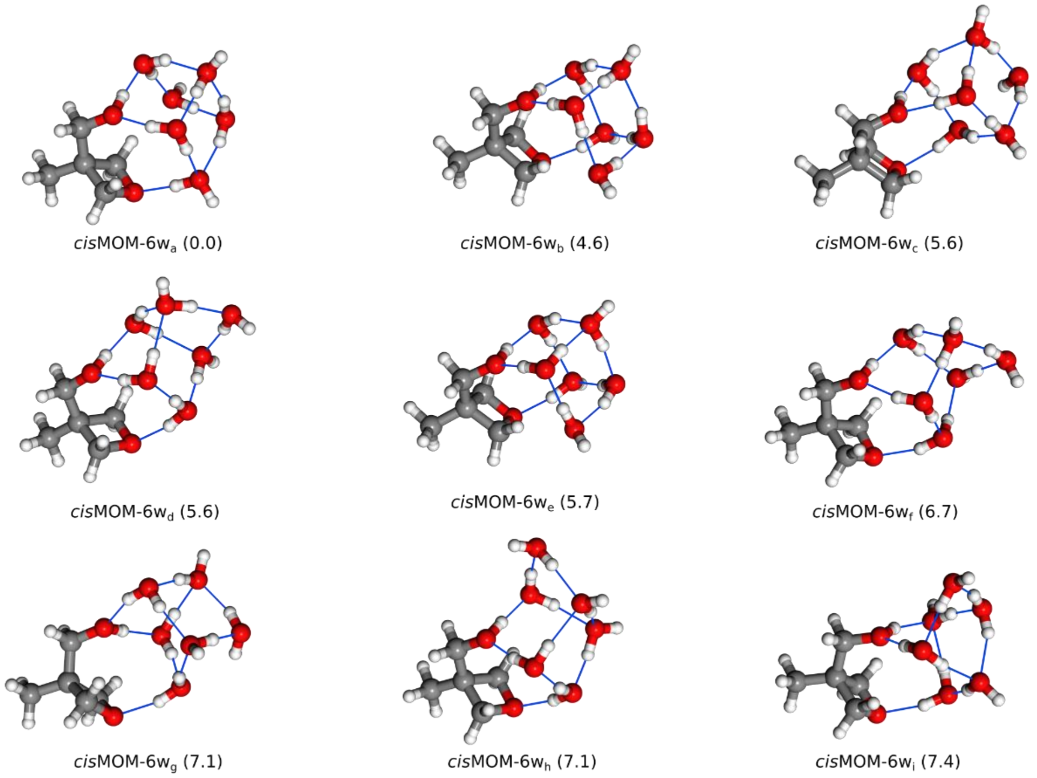

Table S40. Cartesian coordinates (Å) for the equilibrium structure of *cis*MOM-6w$_a$ obtained at the B3LYP-D4/def2-QZVP level of theory.

Atom	a	b	c
C	-2.46703	0.27835	0.01414
C	-3.88572	0.70707	-0.32727
H	-4.35014	0.01188	-1.02908
H	-3.89466	1.70013	-0.78357
H	-4.51	0.74689	0.56917
C	-1.84021	1.24965	1.01342
O	-0.528	0.88913	1.38857
H	0.135	1.46661	0.92608
H	-1.84544	2.25904	0.58689
H	-2.46347	1.27539	1.91458
C	-1.58032	-0.07354	-1.20065
H	-2.06553	0.00369	-2.1755
H	-0.59877	0.39553	-1.24228
O	-1.49104	-1.47001	-0.77986
C	-2.30907	-1.20923	0.39446
H	-1.75619	-1.40466	1.31467
H	-3.2244	-1.80514	0.37379
O	1.48634	1.31223	-2.3712
H	1.92612	1.71004	-3.12825
H	2.01967	0.51622	-2.11723
O	3.28563	0.44305	1.19954
H	4.05615	0.50184	1.77217
H	2.60784	-0.11765	1.66872
O	2.88735	-0.74474	-1.34999
H	2.27326	-1.4519	-1.0817
H	3.21818	-0.36887	-0.51282
O	0.91475	-2.50896	-0.2942
H	0.9091	-3.45798	-0.45318
H	0.01479	-2.16903	-0.53921
O	1.28623	-1.05601	2.12952
H	1.17367	-1.70657	1.41317
H	0.52809	-0.44533	2.0309
O	1.36748	2.31594	0.19984
H	2.15841	1.88767	0.57344
H	1.40134	2.11734	-0.75981

Table S41. Cartesian coordinates (Å) for the equilibrium structure of *cis*MOM-6w$_b$ obtained at the B3LYP-D4/def2-QZVP level of theory.

Atom	a	b	c
C	-2.47668	0.35084	-0.05927
C	-3.92074	0.69294	-0.39664
H	-4.41295	-0.13837	-0.90466
H	-4.49244	0.91953	0.50732
H	-3.97212	1.56599	-1.05168
C	-1.82782	1.50637	0.70422
O	-0.43161	1.40008	0.86229
H	-0.15041	0.64803	1.43419
H	-2.32662	1.60709	1.67792
H	-2.00166	2.43628	0.15637
C	-2.286	-1.04102	0.58056
H	-3.19944	-1.62048	0.73338
H	-1.67526	-1.07287	1.48532
O	-1.54791	-1.52176	-0.57576
C	-1.64924	-0.21921	-1.23405
H	-0.66678	0.22337	-1.3909
H	-2.18099	-0.31023	-2.183
O	1.91546	2.34174	-0.24104
H	1.01905	2.20548	0.12381
H	1.88123	1.91115	-1.12075
O	0.82429	-0.5926	2.21346
H	0.83101	-1.42215	1.70486
H	1.71296	-0.21126	2.07077
O	1.69265	0.65338	-2.43315
H	2.15758	-0.09892	-1.9857
H	2.10179	0.75297	-3.29785
O	0.73381	-2.64158	0.22426
H	-0.10561	-2.29902	-0.17607
H	0.66455	-3.60071	0.25532
O	2.90265	-1.24267	-0.94401
H	2.21275	-1.84239	-0.60681
H	3.18955	-0.72466	-0.16872
O	3.2002	0.563	1.22091
H	3.97983	0.90304	1.6698
H	2.79528	1.32118	0.70235

Table S42. Cartesian coordinates (Å) for the equilibrium structure of *cis*MOM-6w$_c$ obtained at the B3LYP-D4/def2-QZVP level of theory.

Atom	a	b	c
C	2.79278	-0.2274	0.04964
C	4.29135	-0.46651	0.15599
H	4.84234	0.47498	0.12115
H	4.64774	-1.09404	-0.66524
H	4.54211	-0.96942	1.09347
C	2.04341	-1.56152	0.06509
O	0.63967	-1.43715	0.14841
H	0.2412	-1.10563	-0.69459
H	2.33068	-2.13905	-0.82333
H	2.35911	-2.13514	0.94094
C	2.3682	0.74994	-1.07091
H	3.19194	1.18983	-1.63839
H	1.59605	0.39862	-1.75723
O	1.83694	1.69771	-0.10462
C	2.22343	0.84354	1.00604
H	1.3557	0.53356	1.59013
H	2.95729	1.33895	1.64643
O	-2.26911	0.83946	1.90576
H	-2.35736	1.06799	2.8354
H	-2.07662	-0.14182	1.84458
O	-0.93772	1.90671	-0.34259
H	0.02928	2.03335	-0.26759
H	-1.23495	1.63422	0.54463
O	-3.28361	-1.39905	-0.9985
H	-3.99311	-1.89837	-1.41407
H	-3.62622	-0.4841	-0.82856
O	-0.76134	-0.40478	-1.8846
H	-0.9094	0.46131	-1.44455
H	-1.61568	-0.86663	-1.8232
O	-3.86645	1.15305	-0.35556
H	-3.25661	1.73738	-0.82674

H	-3.5165	1.14502	0.55786
O	-1.79558	-1.70667	1.43037
H	-2.34253	-1.84025	0.63479
H	-0.87043	-1.76338	1.11838

Figure S17. Part of the broadband spectrum of *cis*MOM-6w$_a$ collected with 0.5×10^6 FIDs.

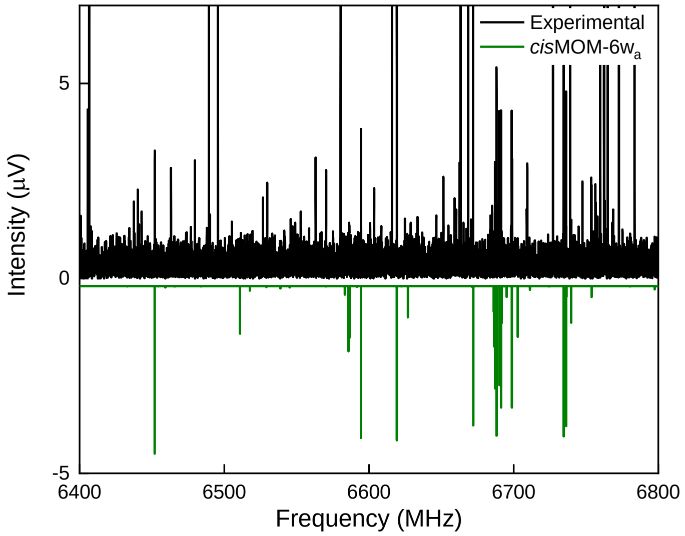

Table S43. Spectroscopic constants for *cis*MOM-6w$_a$, *cis*MOM-6w$_b$, and *cis*MOM-6w$_c$ obtained at the MP2/cc-pVQZ level of theory.

Parameters	*cis*MOM-6w$_a$	*cis*MOM-6w$_b$	*cis*MOM-6w$_c$
A [MHz]	701.8	708.5	886.6
B [MHz]	386.8	388.8	333.6
C [MHz]	372.8	359.2	312.0
$\|\mu_a\|$ [D]	2.0	2.8	2.0
$\|\mu_b\|$ [D]	1.2	1.5	0.9
$\|\mu_c\|$ [D]	0.8	0.4	1.1
ΔE [kJ/mol]	0.0	5.1	4.0

Table S44. Experimental spectroscopic constants of *cis*MOM-6w$_a$.

Parameters	*cis*MOM-6w$_a$
A [MHz]	675.61995(23)[a]
B [MHz]	379.52130(24)
C [MHz]	363.13650(20)
Δ_J [kHz]	0.05067(97)
Δ_{JK} [kHz]	-0.0441(46)
Δ_K [kHz]	0.1604(79)
δ_J [kHz]	-0.00789(62)
δ_K [kHz]	-0.171(51)
a-type[b]	+++
b-type[b]	++

c-type[b]	++
σ [kHz][c]	3.3
N[d]	89

[a] Standard errors within parentheses are expressed in units of the last two digits. [b] Strength of the observed type of transitions: +++ (strong), ++ (medium). [c] Root-mean-square deviation of the fit. [d] number of the lines in the fit.

2.8. Observed Transition Frequencies

Table S45. Assigned transitions for the parent species of MOM-I$_a$.

J'	*K$_a$'*	*K$_c$'*	*J''*	*K$_a$''*	*K$_c$''*	*ν*/MHz	*Δν*/kHz
5	0	5	4	1	4	18253.34	0.4
6	0	6	5	1	4	18344.498	-2.4
3	2	2	2	1	2	18625.28	2.3
3	2	1	2	1	2	18747.166	2.2
7	2	5	6	3	3	18846.013	3.4
6	5	2	6	4	2	18994.02	-7.3
6	5	1	6	4	3	18997.193	2.7
6	1	5	5	2	3	19008.625	0
5	5	1	5	4	1	19017.478	4.6
5	5	0	5	4	2	19018.104	-1.9
5	1	5	4	1	4	19031.994	1.5
3	3	1	3	0	3	19206.738	-7.8
4	1	3	3	0	3	19321.605	-2.9
5	0	5	4	0	4	19347.239	2
5	2	4	4	2	3	19731.845	0.7
6	1	5	5	2	4	19803.748	-3.7
5	4	2	4	4	1	19841.575	6.9
5	4	1	4	4	0	19842.124	-3.3
5	3	3	4	3	2	19857.301	8.2
5	3	2	4	3	1	19887.092	0.3
5	3	3	5	0	5	19996.833	0.9
8	1	8	7	2	6	20015.36	-0.2
8	2	7	7	3	5	20042.196	-2.4
5	1	5	4	0	4	20125.89	0.1
5	2	3	4	2	2	20170.539	-0.8
5	1	4	4	1	3	20323.627	0.3
3	2	1	2	0	2	20494.272	1.6
7	0	7	6	1	5	20767.344	-3.6
6	3	4	6	0	6	20791.693	1.8
4	2	3	3	1	2	21390.15	0.3
4	2	2	3	1	2	21746.581	0
6	0	6	5	1	5	22269.184	4.8
3	3	1	2	2	0	22456.553	1.6
3	3	0	2	2	0	22457.998	1.6
3	3	1	2	2	1	22481.212	3.5
3	3	0	2	2	1	22482.658	4.5
6	1	6	5	1	5	22790.172	1.8
7	1	6	6	2	4	22894.795	1.5
4	2	3	3	1	3	22974.609	0.8
6	0	6	5	0	5	23047.834	1.9
4	2	2	3	1	3	23331.039	-0.3
8	2	6	7	3	4	23465.004	-9.9
6	1	6	5	0	5	23568.825	1.9
6	2	5	5	2	4	23633.503	2.8
6	5	2	5	5	1	23806.112	-4.8
6	5	1	5	5	0	23806.152	4.4
6	4	3	5	4	2	23827.055	-8.2
6	4	2	5	4	1	23829.558	-4.8
6	3	4	5	3	3	23842.692	0.8
6	3	3	5	3	2	23920.348	-3.5
5	1	4	4	0	4	24050.576	7.3
6	1	5	5	1	4	24298.1	1.7
6	2	4	5	2	3	24326.797	-0.5

7	1	6	6	2	5	24383.228	10.3
4	2	2	3	0	3	24764.173	4.9
5	2	4	4	1	3	24817.979	5.8
5	2	3	4	1	3	25613.105	5
2	0	2	1	0	1	7890.0256	-16.5
2	1	2	1	1	1	7650.3781	-9.2
2	1	1	1	1	0	8179.0227	-5.4
3	2	1	3	1	3	7286.3521	-6.4
2	2	0	2	1	2	6777.8646	-6.4
2	2	1	2	1	2	6753.2112	-2.7
3	1	2	2	2	0	6267.3871	-5.5
1	1	0	0	0	0	6208.4341	-9.3
2	2	1	2	1	1	5960.2426	-5.4
1	1	1	0	0	0	5944.1118	-6.5
2	0	2	1	1	1	5903.2635	-17.2
3	2	1	3	1	2	5701.8975	-2.5
2	0	2	1	1	0	5638.947	-8.5
3	2	2	3	1	2	5580.0062	-7.7
4	2	3	4	1	3	5086.1258	-3
3	1	3	2	2	1	4707.5842	-7
5	1	4	5	0	5	4703.3259	-5.7
5	2	4	5	1	4	4494.3402	-6.2
1	0	1	0	0	0	3957.3529	-4
5	1	4	5	1	5	3924.6726	-6.1
6	2	5	6	1	5	3829.7439	-4.5
4	1	3	4	0	4	3726.9392	-2.7
3	1	2	3	0	3	3017.5859	-1.2
4	1	3	4	1	4	2633.0399	-4.6
2	1	1	2	0	2	2540.0711	-1.5
1	1	0	1	0	1	2251.0823	-4.2
8	0	8	7	1	7	19427.74	2.1
5	3	3	4	2	2	19435.22	16.9
7	2	6	6	1	5	19453.3	8.3
8	1	8	7	0	7	19475.79	4.2
4	4	1	3	3	0	19878.68	5.7
4	4	0	3	3	1	19887.78	0.2
5	3	2	4	2	3	20521.03	12.9
8	2	7	7	1	6	21431.93	26
6	3	4	5	2	3	21537.66	-9.4
9	0	9	8	1	8	21782.68	-1.4
5	4	2	4	3	1	22550.34	-1.6
5	4	1	4	3	2	22614.37	9.4
7	3	5	6	2	4	23427.14	-2.7
5	5	1	4	4	0	25177.5	-20.8
5	5	0	4	4	1	25178.46	14.6
6	4	2	5	3	3	25389.48	3.3
3	1	2	3	0	3	2602.702	2.7
4	2	2	4	1	3	3228.029	-1
3	2	1	3	1	2	3252.009	0.6
2	2	0	2	1	1	3468.098	-6.7
5	2	3	5	1	4	3516.13	4.4
4	1	3	4	0	4	3680.683	-0.3
1	1	1	0	0	0	3818.402	1.5
2	2	1	2	1	2	4428.417	-9.5
3	2	2	3	1	3	4980.009	-1.1
7	3	4	7	2	5	4863.755	-0.7
4	3	1	4	2	2	5913.69	-3.7
2	1	2	1	0	1	6160.645	-4.5
3	3	0	3	2	1	6254.095	-12.1
3	3	1	3	2	2	6563.166	-11.3
5	2	4	5	1	5	6651.439	-1.9
6	1	5	6	0	6	6665.308	-5.9
4	3	2	4	2	3	6727.178	-4
3	0	3	2	1	2	6944.578	-8.8
5	3	3	5	2	4	7029.965	-2.4
6	3	4	6	2	5	7504.941	3.1
4	0	4	3	1	2	7581.763	-24
6	2	5	6	1	6	7738.912	-0.9
3	1	3	2	0	2	8360.041	-11.6
2	2	1	1	1	0	9112.919	-9.2

Table S46. Assigned transitions for the $^{13}C_1$ isotopologue of MOM-I_a.

J'	*Ka'*	*Kc'*	*J''*	*Ka''*	*Kc''*	*v*/MHz	*Δv*/kHz
3	2	2	2	1	2	18613.235	-4.9
5	1	5	4	1	4	19026.423	-2.8
4	1	3	3	0	3	19320.117	-3.8
5	0	5	4	0	4	19341.332	5
5	2	4	4	2	3	19728.645	5.8
5	3	3	4	3	2	19855.041	-9.3
5	3	2	4	3	1	19885.241	0.9
5	1	4	4	1	3	20321.884	4.5
4	2	2	3	1	2	21732.976	1.9
3	3	0	2	2	0	22436.871	10.5
3	3	1	2	2	0	22435.391	-5.1
3	3	0	2	2	1	22461.719	-1.1
6	1	6	5	1	5	22783.185	0.6
4	2	3	3	1	3	22963.243	-2
6	0	6	5	0	5	23040.017	-3.2
6	2	5	5	2	4	23629.301	5.6
6	3	4	5	3	3	23840.033	3.7
5	1	4	4	0	4	24051.36	4.9
6	1	5	5	1	4	24295.149	-9.3
5	2	3	4	1	3	25600.414	-2.5

Table S47. Assigned transitions for the $^{13}C_2$ isotopologue of MOM-I$_a$.

J'	*Ka'*	*Kc'*	*J''*	*Ka''*	*Kc''*	*v*/MHz	*Δv*/kHz
3	2	2	2	1	2	18464.71	-3.3
5	1	5	4	1	4	18962.501	0
4	1	3	3	0	3	19167.102	-0.4
5	0	5	4	0	4	19272.305	0.2
5	2	4	4	2	3	19638.086	1.8
5	3	3	4	3	2	19757.093	4.2
5	3	2	4	3	1	19784.744	-1.2
5	2	3	4	2	2	20055.09	1.1
5	1	4	4	1	3	20211.425	-3.1
4	2	2	3	1	2	21588.644	-6.4
3	3	1	2	2	0	22250.588	1.9
3	3	0	2	2	0	22251.928	1.8
3	3	1	2	2	1	22273.926	-1.3
3	3	0	2	2	1	22275.265	-2.4
6	1	6	5	1	5	22709.033	4.3
4	2	3	3	1	3	22781.499	-1.3
6	0	6	5	0	5	22964.408	-4.2
6	2	5	5	2	4	23523.347	-0.8
6	3	4	5	3	3	23722.021	-5.2
6	3	3	5	3	2	23794.172	-0.6
5	1	4	4	0	4	23849.106	2.5
6	1	5	5	1	4	24168.667	5.4
6	2	4	5	2	3	24184.284	-5.6
5	2	3	4	1	3	25432.048	10.9

Table S48. Assigned transitions for the $^{13}C_3$ isotopologue of MOM-I$_a$.

J'	*Ka'*	*Kc'*	*J''*	*Ka''*	*Kc''*	*v*/MHz	*Δv*/kHz
3	2	2	2	1	2	18482.763	-4
5	1	5	4	1	4	18892.553	-5.7
4	1	3	3	0	3	19126.913	0.8
5	0	5	4	0	4	19204.785	3.5
5	2	4	4	2	3	19566.516	3.3
5	3	3	4	3	2	19684.033	2.4
5	3	2	4	3	1	19711.006	7.8
5	2	3	4	2	2	19978.784	-1.5
5	1	5	4	0	4	20005.785	-3.1
5	1	4	4	1	3	20139.658	-4.8
3	3	1	2	2	0	22310.972	-0.5
3	3	0	2	2	0	22312.281	2.2
3	3	1	2	2	1	22333.996	0.8
3	3	0	2	2	1	22335.303	1.6
6	1	6	5	1	5	22625.537	0.6
4	2	3	3	1	3	22784.401	-5.2
6	0	6	5	0	5	22884.178	5.8
6	2	5	5	2	4	23438.012	-7.8
6	4	3	5	4	2	23618.818	-4.9
6	4	2	5	4	1	23621.012	0
6	3	4	5	3	3	23634.352	3

6	3	3	5	3	2	23704.736	-0.1
5	1	4	4	0	4	23792.159	1.5
6	1	5	5	1	4	24083.917	0.3
6	2	4	5	2	3	24092.552	0.9
5	2	3	4	1	3	25415.349	2.6

Table S49. Assigned transitions for the $^{18}O_4$ isotopologue of MOM-I_a.

J'	*K$_a$'*	*K$_c$'*	*J''*	*K$_a$''*	*K$_c$''*	*ν*/MHz	*Δν*/kHz
3	2	2	2	1	2	18357.422	15.1
5	1	5	4	1	4	18507.422	-5
5	0	5	4	0	4	18824.047	-9
4	1	3	3	0	3	18863.015	-1.1
5	2	4	4	2	3	19185.206	8
5	2	3	4	2	2	19596.703	13.3
5	1	4	4	1	3	19762.556	-5.4
4	2	2	3	1	2	21380.566	-2.9
6	1	6	5	1	5	22163.409	8.2
6	0	6	5	0	5	22426.736	-7.5
4	2	3	3	1	3	22585.077	3.4
5	1	4	4	0	4	23455.716	-10.8
6	1	5	5	1	4	23631.9	11.7
6	2	4	5	2	3	23634.769	-1.9
5	2	3	4	1	3	25125.527	-12.1

Table S50. Assigned transitions for the $^{13}C_5$ isotopologue of MOM-I_a.

J'	*K$_a$'*	*K$_c$'*	*J''*	*K$_a$''*	*K$_c$''*	*ν*/MHz	*Δν*/kHz
3	2	2	2	1	2	18509.743	-4.3
5	1	5	4	1	4	18928.592	0.6
4	1	3	3	0	3	19187.649	-5.8
5	0	5	4	0	4	19241.357	4.4
5	2	4	4	2	3	19614.786	0
5	3	3	4	3	2	19736.377	0
5	3	2	4	3	1	19764.844	-6.5
5	2	3	4	2	2	20040.602	0.1
5	1	4	4	1	3	20196.429	2.7
4	2	2	3	1	2	21618.896	1.9
3	3	1	2	2	0	22324.405	-0.3
3	3	0	2	2	0	22325.784	-1.4
3	3	1	2	2	1	22348.272	-1.5
6	1	6	5	1	5	22667.425	1.5
4	2	3	3	1	3	22827.93	2.8
6	0	6	5	0	5	22924.488	-6.3
6	2	5	5	2	4	23494.455	4.7
6	4	2	5	4	1	23684.174	-2.2
6	3	4	5	3	3	23697.376	6.1
5	1	4	4	0	4	23877.237	5.9
6	1	5	5	1	4	24148.606	-3.2
6	2	4	5	2	3	24168.676	-6.7
5	2	3	4	1	3	25458.789	5.5

Table S51. Assigned transitions for the $^{13}C_6$ isotopologue of MOM-I_a.

J'	*K$_a$'*	*K$_c$'*	*J''*	*K$_a$''*	*K$_c$''*	*ν*/MHz	*Δν*/kHz
3	2	2	2	1	2	18313.913	-7.8
3	2	1	2	1	2	18459.887	0.1
5	1	5	4	1	4	18843.44	4.7
5	0	5	4	0	4	19148.739	4.4
4	1	3	3	0	3	19239.938	1.1
5	2	4	4	2	3	19597.586	-1.9
5	3	3	4	3	2	19746.333	-5
5	3	2	4	3	1	19786.25	-5.1
5	2	3	4	2	2	20111.254	7.6
5	1	4	4	1	3	20221.505	2.5
4	2	2	3	1	2	21391.09	-6.8
3	3	1	2	2	0	21936.581	2.5
3	3	0	2	2	0	21938.525	1.7
3	3	1	2	2	1	21966.185	-4.5
3	3	0	2	2	1	21968.132	-2.3
6	1	6	5	1	5	22556.599	3.6
4	2	3	3	1	3	22666.558	-5.3
6	0	6	5	0	5	22793.948	0.5
6	2	5	5	2	4	23463.623	-13.9

6	3	4	5	3	3	23710.004	2.7
6	3	3	5	3	2	23813.397	2.7
5	1	4	4	0	4	24009.261	1.9
6	1	5	5	1	4	24154.632	-5.4
6	2	4	5	2	3	24261.666	1.9
5	2	3	4	1	3	25269.845	6.8
4	3	2	3	2	2	25952.725	10.4

Table S52. Assigned transitions for the $^{18}O_7$ isotopologue of MOM-I$_a$.

J'	K_a'	K_c'	J''	K_a''	K_c''	ν/MHz	$\Delta\nu$/kHz
3	2	2	2	1	2	18310.868	6.2
5	1	5	4	1	4	18365.681	5.8
5	0	5	4	0	4	18683.022	3.6
4	1	3	3	0	3	18719.741	4.9
5	2	4	4	2	3	19026.123	-4.5
5	2	3	4	2	2	19416.753	-6.6
5	1	4	4	1	3	19591.946	-3.5
4	2	2	3	1	2	21311.65	-4.8
6	1	6	5	1	5	21995.411	-6.2
3	3	0	2	2	0	22255.367	-2.1
6	0	6	5	0	5	22262.965	4.9
3	3	1	2	2	1	22275.828	-1.5
4	2	3	3	1	3	22497.026	-13.1
5	1	4	4	0	4	23259.584	4.7
6	2	4	5	2	3	23415.947	2.4
6	1	5	5	1	4	23432.457	-6.3
5	2	3	4	1	3	25015.795	15.7

Table S53. Assigned transitions for the $^{2}H_8$ isotopologue of MOM-I$_a$.

J'	K_a'	K_c'	J''	K_a''	K_c''	ν/MHz	$\Delta\nu$/kHz
6	1	5	5	2	3	18181.852	2
3	2	2	2	1	2	18291.887	0
3	2	1	2	1	2	18406.201	0.1
5	1	5	4	1	4	18420.471	1
5	0	5	4	0	4	18736.625	-1.6
4	1	3	3	0	3	18793.569	0.1
6	1	5	5	2	4	18930.883	2.2
5	2	4	4	2	3	19100.076	0.8
6	5	1	6	4	3	19177.55	-0.5
5	5	1	5	4	1	19196.662	1.8
5	4	2	4	4	1	19202.921	1.2
5	4	1	4	4	0	19203.403	-3.3
5	3	3	4	3	2	19218.09	-2.2
5	3	2	4	3	1	19245.03	-0.2
5	2	3	4	2	2	19514.25	-0.1
5	1	5	4	0	4	19552.424	0.2
5	1	4	4	1	3	19678.51	-2.7
7	0	7	6	1	5	20032.939	-0.9
3	2	1	2	0	2	20182.252	-1.4
4	2	3	3	1	2	20962.695	-2.6
4	2	2	3	1	2	21297.555	1.5
6	0	6	5	1	5	21505.395	-5.3
7	1	6	6	2	4	21972.41	-0.3
6	1	6	5	1	5	22058.78	3
3	3	1	2	2	0	22173.841	-4.3
3	3	0	2	2	0	22175.145	-4.9
3	3	1	2	2	1	22196.955	2.8
3	3	0	2	2	1	22198.256	-0.7
6	0	6	5	0	5	22321.2	2.4
4	2	3	3	1	3	22503.539	0.9
4	2	2	3	1	3	22838.396	2.1
6	1	6	5	0	5	22874.579	4.9
6	3	3	5	3	2	23145.663	1.3
5	1	4	4	0	4	23371.559	-1.7
6	1	5	5	1	4	23530.312	1.4
6	2	4	5	2	3	23536.12	-5.9
5	2	4	4	1	3	24277.945	2.4
4	2	2	3	0	3	24306.298	6
5	2	3	4	1	3	25026.977	3.7
7	1	7	6	1	6	25680.687	-2.9
7	0	7	6	0	6	25876.987	0
4	3	2	3	2	1	25955.508	-2

4	3	1	3	2	1	25964.599	-0.6
6	3	4	5	3	3	23075.338	1.6

Table S54. Assigned transitions for the parent species of MOM-II$_a$.

J'	*K$_a$'*	*K$_c$'*	*J''*	*K$_a$''*	*K$_c$''*	*ν*/MHz	*Δν*/kHz
4	1	3	3	2	2	18698.318	-1.7
4	2	3	3	2	2	18742.169	0.9
3	3	0	2	2	1	18942.566	5.9
4	1	3	3	1	2	18947.304	2.8
4	2	3	3	1	2	18991.15	0.5
4	2	2	3	3	1	19460.645	-4.8
5	2	3	4	4	0	19613.777	-16.6
3	3	0	2	1	1	19755.42	13.4
5	3	3	4	4	0	19781.454	-8.1
4	3	2	3	3	1	20144.171	-1.9
3	2	1	2	1	2	20409.756	-9.8
3	2	1	2	0	2	20461.883	0.5
5	1	5	4	1	4	20697.51	-1.8
5	0	5	4	0	4	20698.096	-6
4	2	2	3	2	1	21079.957	7.1
4	3	1	3	3	0	21590.274	8.3
4	3	2	3	2	1	21763.477	4
3	3	1	2	1	2	22029.069	3.1
3	3	1	2	0	2	22081.187	4.4
5	1	4	4	2	3	22748.092	-3.9
5	2	4	4	2	3	22753.856	-3.6
5	1	4	4	1	3	22791.944	-0.2
5	2	4	4	1	3	22797.707	-0.9
5	3	2	4	4	1	24245.872	11
4	4	1	3	3	0	24255.957	10.2
5	2	3	4	3	2	24429.906	0.8
5	3	3	4	3	2	24597.579	5.3
6	0	6	5	0	5	24630.199	-31.9
6	1	6	5	0	5	24630.199	-37
6	1	6	5	1	5	24630.199	22.4
6	0	6	5	1	5	24630.199	27.5
4	4	0	3	3	1	24960.303	18.5
5	2	3	4	2	2	25113.438	9.7
5	3	3	4	2	2	25281.096	-0.7
5	4	2	4	4	1	25648.486	0.8
3	3	0	3	2	1	2068.6694	-8.6
3	2	1	3	2	2	2352.9792	-6.7
4	3	1	4	2	2	2578.995	1.1
3	2	1	3	1	2	2601.9658	-1.5
2	1	1	2	1	2	2723.0424	5
4	4	0	4	3	1	2920.6219	-18.8
2	2	1	2	1	2	3535.8867	2.8
2	2	1	2	0	2	3588.0005	0
3	3	1	3	2	2	3972.2701	-15.8
4	2	2	4	2	3	4690.7653	-2.4
4	2	2	4	1	3	4734.6128	-3.2
1	0	1	0	0	0	4840.3253	-10.9
3	1	2	3	1	3	4996.6788	1.1
3	1	2	3	0	3	5003.0822	0.9
1	1	1	0	0	0	5111.2751	-8.8
3	2	2	3	1	3	5245.6643	5.2
3	2	2	3	0	3	5252.0637	1
4	3	2	4	2	3	5374.2934	2.6
4	3	2	4	1	3	5418.1359	-3.1
5	4	2	5	3	3	5612.0585	-4.8
5	4	2	5	2	3	5779.7244	-7.4
5	2	3	5	2	4	7050.3153	-21
5	2	3	5	1	4	7056.0909	-9.1
4	1	3	4	1	4	7181.2424	-4.1
4	1	3	4	0	4	7181.8935	-2.7
5	3	3	5	2	4	7217.9998	-5
5	3	3	5	1	4	7223.7634	-5.1
4	2	3	4	1	4	7225.0947	-0.1
4	2	3	4	0	4	7225.7456	1
2	1	2	1	1	1	8772.9589	-12.8
2	0	2	1	0	1	8991.7804	-22.3
2	1	2	1	0	1	9043.9003	-19.1

Table S55. Assigned transitions for the $^{13}C_1$ isotopologue of MOM-II$_a$.

J'	*K$_a$'*	*K$_c$'*	*J''*	*K$_a$''*	*K$_c$''*	*ν*/MHz	*Δν*/kHz
4	1	3	3	2	2	18677.226	1.5
4	2	3	3	2	2	18720.528	-3.4
3	3	0	2	2	1	18923.291	11.7
4	1	3	3	1	2	18924.195	1.3
4	2	3	3	1	2	18967.504	3.2
4	2	2	3	3	1	19445.875	-7
3	2	1	2	1	2	20393.087	8.9
3	2	1	2	0	2	20444.76	3.2
5	1	5	4	1	4	20670.642	0.2
5	0	5	4	0	4	20671.215	-7.4
4	2	2	3	2	1	21057.984	1.4
4	3	1	3	3	0	21572.507	-5.1
4	3	2	3	2	1	21736.352	10
5	2	4	4	2	3	22726.395	-2.8
5	1	4	4	2	3	22720.727	-2.2
5	1	4	4	1	3	22764.039	2.7
5	2	4	4	1	3	22769.703	-1.8
4	4	1	3	3	0	24226.591	-1
5	2	3	4	3	2	24405.297	12.2
5	3	3	4	3	2	24570.947	2.3
6	1	6	5	1	5	24598.047	29.3
6	0	6	5	0	5	24598.047	-23.9
4	4	0	3	3	1	24933.694	-27.8
5	2	3	4	2	2	25083.645	0.7

Table S56. Assigned transitions for the $^{13}C_2$/$^{13}C_3$ isotopologue of MOM-II$_a$.

J'	*K$_a$'*	*K$_c$'*	*J''*	*K$_a$''*	*K$_c$''*	*ν*/MHz	*Δν*/kHz
4	1	3	3	2	2	18597.475	0.3
4	2	3	3	2	2	18639.6	6.1
3	3	0	2	2	1	18771.729	-0.5
4	1	3	3	1	2	18838.461	0.1
4	2	3	3	1	2	18880.58	0
4	2	2	3	3	1	19356.893	-16.2
3	3	0	2	1	1	19563.532	-1.2
4	3	2	3	3	1	20019.128	18.4
3	2	1	2	1	2	20216.813	4.5
3	2	1	2	0	2	20267.228	5.7
5	1	5	4	1	4	20619.117	-4.7
5	0	5	4	0	4	20619.687	2.2
4	2	2	3	2	1	20934.369	-6.6
4	3	1	3	3	0	21442.725	-1.5
4	3	2	3	2	1	21596.582	6
3	3	1	2	1	2	21794.262	-12.8
3	3	1	2	0	2	21844.685	-3.6
5	1	4	4	2	3	22631.628	-3
5	2	4	4	2	3	22637.124	-2.4
5	1	4	4	1	3	22673.747	-3.2
5	2	4	4	1	3	22679.248	2.4
4	4	1	3	3	0	24039.99	5.5
5	2	3	4	3	2	24287.644	-5.4
5	3	3	4	3	2	24448.811	-2.7
6	1	6	5	1	5	24539.99	2.4
6	0	6	5	1	5	24539.99	7.1
6	0	6	5	0	5	24540.039	0
6	1	6	5	0	5	24540.039	-4.8
4	4	0	3	3	1	24736.379	-2.6
5	2	3	4	2	2	24949.849	-0.8
5	3	3	4	2	2	25111.014	-0.1
5	4	2	4	4	1	25485.262	8
4	3	1	3	2	2	25777.562	5.3

Table S57. Assigned transitions for the $^{18}O_4$ isotopologue of MOM-II$_a$.

J'	*K$_a$'*	*K$_c$'*	*J''*	*K$_a$''*	*K$_c$''*	*ν*/MHz	*Δν*/kHz
4	1	3	3	2	2	18422.184	2.2
4	2	3	3	2	2	18433.849	-1.6
4	1	3	3	1	2	18527.3	1.6
4	2	3	3	1	2	18538.965	-2.2
3	3	0	2	2	1	18614.106	-0.7
5	1	5	4	1	4	20263.533	0.9
4	2	2	3	2	1	20766.723	-1.8

4	3	1	3	3	0	21710.085	0.8
5	1	4	4	1	3	22328.98	-0.1
4	4	1	3	3	0	23421.675	1.2
6	0	6	5	0	5	24110.375	-3.4
6	1	6	5	1	5	24110.375	2.5
6	1	6	5	0	5	24110.375	-3.7
6	0	6	5	1	5	24110.375	2.9

Table S58. Assigned transitions for the $^{13}C_5$ isotopologue of MOM-I$_a$.

J'	*K$_a$'*	*K$_c$'*	*J''*	*K$_a$''*	*K$_c$''*	*ν*/MHz	*Δν*/kHz
4	1	3	3	2	2	18566.785	0.7
4	2	3	3	2	2	18609.524	-0.8
3	3	0	2	2	1	18828.313	3.9
4	1	3	3	1	2	18811.479	0
4	2	3	3	1	2	18854.217	-2.5
4	2	2	3	3	1	19339.763	0.5
3	3	0	2	1	1	19632.726	-1.5
5	1	5	4	1	4	20537.048	3.2
5	0	5	4	0	4	20537.621	5.2
4	2	2	3	2	1	20942.365	-1.3
4	3	1	3	3	0	21459.783	-1.2
4	3	2	3	2	1	21614.811	1.7
5	2	4	4	2	3	22588.478	-3.4
5	1	4	4	2	3	22582.902	-6.3
5	2	4	4	1	3	22631.216	-6
4	4	1	3	3	0	24098.467	2.5
5	3	3	4	3	2	24430.389	7.4
6	1	6	5	1	5	24438.135	-1.3
6	0	6	5	1	5	24438.135	3.5
6	0	6	5	0	5	24438.189	0.5
6	1	6	5	0	5	24438.189	-4.3
4	4	0	3	3	1	24806.836	-4.3
5	2	3	4	2	2	24939.277	3.1

Table S59. Assigned transitions for the $^{13}C_6$ isotopologue of MOM-II$_a$.

J'	*K$_a$'*	*K$_c$'*	*J''*	*K$_a$''*	*K$_c$''*	*ν*/MHz	*Δν*/kHz
4	1	3	3	2	2	18410.406	4.6
4	2	3	3	2	2	18454.54	6.1
4	1	3	3	1	2	18659.938	7.4
3	3	0	2	2	1	18701.138	-7.9
4	2	3	3	1	2	18704.071	7.9
3	3	0	2	1	1	19512.837	-7.4
4	3	2	3	3	1	19846.594	-4
5	1	5	4	1	4	20353.874	0.9
5	0	5	4	0	4	20354.458	-11.3
4	2	2	3	2	1	20778.9	-3.9
4	3	1	3	3	0	21282.026	-5
4	3	2	3	2	1	21463.561	2.7
5	1	4	4	2	3	22392.886	-0.3
5	2	4	4	2	3	22398.711	-0.5
5	2	4	4	1	3	22442.844	0
6	1	6	5	1	5	24218.969	4.4
6	0	6	5	1	5	24218.969	9.6
6	0	6	5	0	5	24219.02	0.2
6	1	6	5	0	5	24219.02	-4.9
5	3	3	4	3	2	24230.762	-1.8
5	2	3	4	2	2	24746.723	-6.5
4	3	1	3	1	2	25931.063	13.2

Table S60. Assigned transitions for the $^{18}O_7$ isotopologue of MOM-II$_a$.

J'	*K$_a$'*	*K$_c$'*	*J''*	*K$_a$''*	*K$_c$''*	*ν*/MHz	*Δν*/kHz
4	2	3	3	2	2	18123.362	-2.8
4	1	3	3	1	2	18498.403	-3
3	3	0	2	2	1	18623.688	1.6
5	1	5	4	1	4	20072.096	2.2
5	0	5	4	0	4	20074.779	-2.2
4	3	2	3	2	1	21644.949	1
4	3	1	3	3	0	20387.053	1.1
4	2	3	3	1	2	18635.391	3.7
5	2	4	4	2	3	22073.682	0.7

4	4	0	3	3	1	24664.115	-2.2

Table S61. Assigned transitions for the $^{2}H_{8}$ isotopologue of MOM-II$_{a}$.

J'	*Ka'*	*Kc'*	*J''*	*Ka''*	*Kc''*	*v*/MHz	*Δv*/kHz
4	1	3	3	2	2	18459.807	-9.9
4	2	3	3	2	2	18525.023	6.4
4	1	3	3	1	2	18780.439	-1.3
3	3	0	2	2	1	18809.481	-4.1
4	2	3	3	1	2	18845.647	6.9
4	2	2	3	3	1	18980.649	5
3	3	0	2	1	1	19738.553	1.1
4	3	2	3	3	1	19843.994	-1.5
3	2	1	2	1	2	20155.348	3.2
3	2	1	2	0	2	20223.301	5.5
5	1	5	4	1	4	20469.167	4.9
5	0	5	4	0	4	20470.159	-2.5
4	2	2	3	2	1	20829.216	7
4	3	1	3	3	0	21187.741	0.1
4	3	2	3	2	1	21692.553	-7.5
3	3	1	2	1	2	22003.908	-1.7
3	3	1	2	0	2	22071.864	3.5
5	1	4	4	2	3	22500.276	-5.9
5	2	4	4	2	3	22510.14	-5
5	1	4	4	1	3	22565.479	-2.6
5	2	4	4	1	3	22575.336	-8.6
5	2	3	4	3	2	24045.268	6.2
4	4	1	3	3	0	24219.525	9.7
5	3	3	4	3	2	24290.867	-8.3
4	4	0	3	3	1	24820.718	-8.1
5	2	3	4	2	2	24908.616	2.6
5	3	3	4	2	2	25154.239	12.1
5	4	2	4	4	1	25233.4	2.9
4	3	1	3	2	2	25673.999	-3.9
4	3	1	3	1	2	25994.619	-7.3
6	1	6	5	1	5	24357.27	2.9

Table S62. Assigned transitions for the parent species of *cis*MOM-1w$_{a}$.

J'	*Ka'*	*Kc'*	*J''*	*Ka''*	*Kc''*	*v*/MHz	*Δv*/kHz
7	2	6	6	2	5	18304.717	6.9
7	1	6	6	1	5	18768.877	-0.6
7	3	5	6	3	4	18970.278	5.2
7	5	2	6	5	1	19034.182	0.8
7	5	3	6	5	2	19030.019	11.2
7	4	4	6	4	3	19088.421	4.2
7	4	3	6	4	2	19180.313	3.5
8	1	8	7	1	7	19442.235	5.7
8	0	8	7	0	7	19461.296	5.4
7	3	4	6	3	3	19683.029	-2.2
7	2	5	6	2	4	19872.232	-1.7
8	2	7	7	2	6	20747.481	3.5
8	1	7	7	1	6	21059.882	4.8
8	3	6	7	3	5	21604.101	-0.5
9	1	9	8	1	8	21788.78	2.3
9	0	9	8	0	8	21797.177	4.6
8	5	4	7	5	3	21801.057	-4
8	5	3	7	5	2	21817.215	4.3
8	4	5	7	4	4	21851.833	10
8	4	4	7	4	3	22083.078	2.1
8	2	6	7	2	5	22529.839	-6.3
8	3	5	7	3	4	22708.003	-1.4
9	2	8	8	2	7	23152.41	3.4
9	1	8	8	1	7	23335.95	0.7
10	1	10	9	1	9	24132.672	-3.9
10	0	10	9	0	9	24136.256	-3.1
9	3	7	8	3	6	24183.089	0.8
9	4	6	8	4	5	24598.803	13.3
9	5	5	8	5	4	24586.974	-28.6
9	5	4	8	5	3	24636.925	-1.9
9	2	7	8	2	6	25030.828	-2.8
9	4	5	8	4	4	25080.471	4.1
10	2	9	9	2	8	25530.124	-30.3
10	1	9	9	1	8	25627.958	18.8

9	3	6	8	3	5	25665.726	2.7
3	1	2	2	1	1	8520.4753	-10.5
4	1	4	3	1	3	9941.6764	-17.4
3	2	1	2	2	0	8304.3816	-5.6
3	2	2	2	2	1	8053.5384	-2.6
3	0	3	2	0	2	7802.6716	-9.7
3	1	3	2	1	2	7501.952	-5.8
2	1	1	1	1	0	5711.3019	1.2
2	0	2	1	0	1	5302.5495	-6.2
2	1	2	1	1	1	5026.7708	1
5	1	4	5	1	5	4917.9372	0.8
1	0	1	0	0	0	2684.5169	-3.9

Table S63. Assigned transitions for the parent species of *cis*MOM-2w$_a$.

J'	*K$_a$*'	*K$_c$*'	*J*''	*K$_a$*''	*K$_c$*''	*ν*/MHz	*Δν*/kHz
4	2	2	4	1	3	2411.6587	-0.6
3	2	1	3	1	2	2484.7361	3.6
5	2	3	5	1	4	2504.2054	-2.9
1	1	1	0	0	0	2648.1716	4.4
6	2	4	6	1	5	2816.9296	-4.7
1	1	0	0	0	0	2859.2593	-5.7
7	3	5	7	2	5	3128.5748	-1.2
2	1	2	1	1	1	3342.5256	-0.1
2	0	2	1	0	1	3519.7096	-1.1
3	2	2	3	1	3	3583.6977	-1.7
8	3	5	8	2	6	3663.5911	-2.4
6	3	4	6	2	4	3701.6047	-3.5
7	3	4	7	2	5	3729.1327	1.6
2	1	1	1	1	0	3764.7178	-3.5
6	3	3	6	2	4	3964.3256	-7.2
4	2	3	4	1	4	4038.0195	3.5
5	3	3	5	2	3	4178.9218	1.3
2	1	2	1	0	1	4213.8795	2.5
5	3	2	5	2	3	4271.459	-1.5
4	2	2	4	1	4	4498.7833	0.2
3	0	3	2	1	2	4506.0235	2.7
4	3	1	4	2	2	4552.475	-4
5	2	4	5	1	5	4607.5868	-6.6
2	1	1	1	0	1	4847.1727	2.2
4	3	2	4	2	3	4989.3942	0.4
3	1	3	2	1	2	4994.0441	-2.4
4	3	1	4	2	3	5013.2477	1.6
4	1	3	3	2	2	5129.7395	-14.3
5	3	3	5	2	4	5149.4312	-3.2
3	0	3	2	0	2	5200.1859	-1.1
5	3	2	5	2	4	5241.9749	0.3
3	2	2	2	2	1	5330.4125	5.6
6	3	4	6	2	5	5405.623	-6.2
3	2	1	2	2	0	5460.6515	1.2
3	1	2	2	1	1	5623.8801	-0.2
3	1	3	2	0	2	5688.2127	-0.1
5	0	5	4	1	3	5980.775	-4.5
4	0	4	3	1	3	6322.2952	-0.6
2	2	1	1	1	0	6378.767	0
2	2	0	1	1	0	6412.6879	4
6	4	3	6	3	3	6519.5846	-7.8
2	2	1	1	1	1	6589.868	3
2	2	0	1	1	1	6623.7861	4.3
4	1	4	3	1	3	6626.33	0.3
6	0	6	5	1	4	6671.8695	-0.3
5	4	2	5	3	2	6684.0275	-0.6
5	4	1	5	3	2	6686.6189	-10
5	4	2	5	3	3	6776.571	2.7
5	4	1	5	3	3	6779.1634	-5.6
4	0	4	3	0	3	6810.3173	-4.3
5	1	4	4	2	2	6814.2989	-6.9
3	1	2	2	0	2	6951.3411	1
4	2	3	3	2	2	7080.6397	-6.4
4	1	4	3	0	3	7114.3523	-3.1
4	3	2	3	3	1	7165.8366	-1.1
4	3	1	3	3	0	7186.2282	-2.7
4	2	2	3	2	1	7377.2519	-1
4	1	3	3	1	2	7450.3213	-4.7
3	2	2	2	1	1	7944.4582	5.6
5	0	5	4	1	4	8067.9028	-0.4

5	1	5	4	1	4	8239.1927	2.9
5	1	5	4	0	4	8543.2267	3.1
5	1	4	4	1	3	9225.9606	-4.6
8	2	6	7	1	6	18325.78	14.7
10	2	8	9	2	7	18326.51	0
8	3	5	7	2	5	18605.233	-2.8
7	4	4	6	3	3	19113.568	-2.1
7	4	3	6	3	3	19159.123	2.9
7	4	4	6	3	4	19376.316	21.2
7	4	3	6	3	4	19421.853	8.2
6	5	2	5	4	1	19465.91	-15.5
6	5	1	5	4	1	19466.174	5.6
6	5	2	5	4	2	19468.53	3.5
6	5	1	5	4	2	19468.779	9.7
8	1	7	7	0	7	19471.953	14.4
8	2	7	7	1	7	19879.176	-13.3
8	3	6	7	2	6	20088.856	-24.2
9	3	6	8	2	6	20519.676	-8.8
8	4	5	7	3	4	20642.571	2.8
8	4	4	7	3	4	20773.277	9.9
9	2	7	8	1	7	20885.669	-1.5
7	5	3	6	4	2	21241.555	-7.9
7	5	2	6	4	2	21243.023	21.1
7	5	3	6	4	3	21254.35	6.4
7	5	2	6	4	3	21255.769	-13.4
6	6	0	5	5	0	21411.684	3.6
6	6	1	5	5	0	21411.684	5.2
6	6	1	5	5	1	21411.701	0
6	6	0	5	5	1	21411.701	-1.6
8	5	3	7	4	3	23001.136	10.9
8	5	4	7	4	4	23040.54	-15.3
7	7	0	6	6	0	25141.934	-3.6
7	7	0	6	6	1	25141.934	-5.2
7	7	1	6	6	0	25141.934	-3.5
7	7	1	6	6	1	25141.934	-5

Table S64. Assigned transitions for the parent species of *g*MOM-2w$_b$.

J'	*K$_a$'*	*K$_c$'*	*J''*	*K$_a$''*	*K$_c$''*	*ν*/MHz	*Δν*/kHz
4	2	2	4	1	3	2030.0525	3.3
1	1	1	0	0	0	2420.4641	4.2
5	3	2	5	2	3	2460.4483	3
4	3	1	4	2	2	2625.739	1.7
6	3	3	6	2	4	2685.0074	0.7
5	2	3	5	1	4	2832.7985	1.5
2	0	2	1	1	1	3183.3457	5.2
3	2	2	3	1	3	3184.2933	-2.5
7	4	3	7	3	4	3274.7397	-1
8	4	4	8	3	5	3339.0944	5.3
4	1	3	4	0	4	3360.4801	-3.1
6	4	2	6	3	3	3609.3858	6.2
4	3	2	4	2	3	3792.4502	1
4	2	3	4	1	4	3974.9796	0.4
2	1	2	1	0	1	3979.1925	4.2
6	2	4	6	1	5	4006.4375	-2.5
5	4	1	5	3	2	4082.6492	-0.9
5	3	3	5	2	4	4290.1474	1
5	1	4	5	0	5	4611.0232	2.6
5	4	2	5	3	3	4714.257	1.4
5	2	4	5	1	5	4913.084	2.6
6	4	3	6	3	4	4930.0613	5.2
6	3	4	6	2	5	4994.2219	-2.1
3	0	3	2	1	2	5013.0606	-1
3	1	3	2	1	2	5134.4097	-8.2
7	5	2	7	4	3	5201.8329	4.4
3	0	3	2	0	2	5299.6354	-6.6
7	2	5	7	1	6	5333.8486	-2.8
7	4	4	7	3	5	5342.5891	-4
3	1	3	2	0	2	5420.9974	-0.9
6	5	1	6	4	2	5628.7732	-6.9
2	2	1	1	1	0	5702.6375	2.1
6	1	5	6	0	6	5821.4134	5.7
5	5	0	5	4	1	5854.3064	-7.8
6	5	2	6	4	3	5866.3352	0.7
7	3	5	7	2	6	5872.0127	-0.2

7	5	3	7	4	4	5883.1107	-3
5	5	1	5	4	2	5909.2726	-3.9
6	2	5	6	1	6	5943.6881	1.6
9	3	6	9	2	7	5962.1566	-9
8	4	5	8	3	6	5969.825	-2.8
3	1	2	2	1	1	6159.3868	-5.5
2	2	0	1	1	1	6184.8689	4.3
8	2	6	8	1	7	6628.0484	-3.1
4	0	4	3	1	3	6707.2466	-4.6
4	1	4	3	1	3	6749.6381	-3.2
4	0	4	3	0	3	6828.6015	-6
4	1	4	3	0	3	6870.9943	-3.2
4	1	3	3	2	2	6883.4374	-1.3
7	1	6	7	0	7	6977.8861	-3.1
7	2	6	7	1	7	7021.4455	-0.5
9	6	4	9	5	5	7023.2332	-10.3
7	6	2	7	5	3	7140.0228	-4.6
6	6	0	6	5	1	7209.6537	-4.1
6	6	1	6	5	2	7222.2871	-7
3	2	2	2	1	1	7261.3472	3.5
4	2	3	3	2	2	7540.3211	-3.5
4	3	2	3	3	1	7834.8407	-1.1
9	2	7	9	1	8	7841.6606	-2.4
9	3	7	9	2	8	7936.6969	-10.7
4	1	3	3	1	2	7985.3875	-2.5
5	0	5	4	1	4	8319.5837	-7.5
5	1	5	4	0	4	8375.2187	-6
4	2	3	3	1	2	8642.275	-0.9
5	1	4	4	2	3	8955.6205	-12.1
3	3	1	2	2	0	9101.7017	-0.3
3	3	0	2	2	1	9263.8634	-0.3
8	4	5	7	3	4	18266.069	-0.6
10	2	8	9	3	7	18306.402	8.5
10	3	8	9	2	7	18441.326	20
11	2	10	10	1	9	18803.537	-9.9
6	6	1	5	5	0	19028.988	6.9
6	6	0	5	5	1	19030.431	14.5
9	4	6	8	3	5	19229.245	-29.3
12	1	12	11	0	11	19255.301	-9.2
12	0	12	11	1	11	19255.301	-2.9
7	5	3	6	4	2	19462.557	6.7
7	4	3	6	3	4	19512.478	9
7	5	2	6	4	3	19784.243	1.6
12	1	11	11	2	10	20360.093	-23.1
12	2	11	11	1	10	20360.63	25.2
7	6	2	6	5	1	20973.806	8.4
7	6	1	6	5	2	20989.298	8.6
8	5	4	7	4	3	21019.79	-3.5
7	3	4	6	2	5	21231.944	-8.2
8	5	3	7	4	4	22044.155	18.5
9	5	5	8	4	4	22253.205	4.9
7	7	1	6	6	0	22311.778	7.2
7	7	0	6	6	1	22312.025	7.7
8	6	3	7	5	2	22875.765	-2.3
8	6	2	7	5	3	22964.469	-8
8	7	2	7	6	1	24263.861	-0.4
8	7	1	7	6	2	24267.002	-10.2
9	6	4	8	5	3	24656.994	-0.8
9	6	3	8	5	4	25008.29	2.2
8	8	1	7	7	0	25593.91	-5
8	8	0	7	7	1	25593.957	1.3

Table S65. Assigned transitions for the parent species of *cis*MOM-3w$_a$.

J'	*K$_a$'*	*K$_c$'*	*J''*	*K$_a$''*	*K$_c$''*	*ν*/MHz	*Δν*/kHz
3	1	3	2	1	2	4348.7947	1.6
3	0	3	2	0	2	4370.5964	-3.7
4	0	4	3	1	3	5256.0759	-7.5
7	5	3	7	4	4	5527.7788	-1.6
7	5	2	7	4	3	5527.7788	3.4
6	5	2	6	4	3	5528.1873	-4
6	5	1	6	4	2	5528.1873	-2.6
5	5	1	5	4	2	5528.4508	-2.4
5	5	0	5	4	1	5528.4508	-2.1
6	2	5	5	3	2	5667.7974	-3.8
6	2	4	5	3	3	5687.4057	-5.5

4	1	4	3	1	3	5798.0601	-1.5
4	0	4	3	0	3	5826.155	4.7
4	2	3	3	2	2	5828.7351	2.9
4	2	2	3	2	1	5831.5377	2.2
4	1	3	3	1	2	5858.7295	9.3
3	2	2	2	1	1	6192.012	1
4	1	4	3	0	3	6368.1281	-0.3
4	1	3	3	0	3	6519.8118	2.5
5	0	5	4	1	4	6738.613	5.3
7	6	2	7	5	3	6756.7474	-6.4
7	6	1	7	5	2	6756.7474	-6.3
6	6	1	6	5	2	6756.9721	-5.6
6	6	0	6	5	1	6756.9721	-5.6
8	6	3	8	5	4	6756.4303	5.9
8	6	2	8	5	3	6756.4303	6
10	6	5	10	5	6	6755.329	1.1
10	6	4	10	5	5	6755.329	2
6	1	5	5	2	4	7056.0907	-15.2
5	1	5	4	1	4	7247.0546	-3.3
5	0	5	4	0	4	7280.5856	-0.3
5	2	4	4	2	3	7285.5489	2.1
5	4	1	4	4	0	7286.7886	3.9
5	4	2	4	4	1	7286.7886	4.1
5	3	3	4	3	2	7287.1	1.7
5	3	2	4	3	1	7287.1678	1.3
5	2	3	4	2	2	7291.1375	2.4
5	1	4	4	1	3	7322.8375	6.7
4	2	3	3	1	2	7626.448	6
4	2	2	3	1	3	7721.6774	5.7
5	1	5	4	0	4	7789.028	-8.1
8	7	2	8	6	3	7985.2471	-1.9
8	7	1	8	6	2	7985.2471	-1.9
9	7	3	9	6	4	7984.9479	-16.4
9	7	2	9	6	3	7984.9479	-16.4
6	0	6	5	1	5	8225.211	1.7
6	1	6	5	1	5	8695.7142	-11.5
6	0	6	5	0	5	8733.6561	-3.3
6	2	4	5	2	3	8751.8367	-2.5
6	3	4	5	3	3	8744.8297	0.6
6	4	2	5	4	1	8744.3172	-5.1
6	4	3	5	4	2	8744.3172	-4
6	3	3	5	3	2	8745.0047	-6.1
7	7	0	6	6	1	18186.787	13.3
7	7	1	6	6	0	18186.787	13.3
8	6	2	7	5	3	18415.526	6.2
8	6	3	7	5	2	18415.526	6.2
9	5	4	8	4	5	18643.889	-5.5
9	5	5	8	4	4	18643.889	10.1
10	4	7	9	3	6	18868.644	12.3
10	4	6	9	3	7	18871.711	1.8
8	7	1	7	6	2	19644.016	0.9
8	7	2	7	6	1	19644.016	0.9
9	6	3	8	5	4	19872.69	8.9
9	6	4	8	5	3	19872.69	9
8	8	0	7	7	1	20872.436	-0.5
8	8	1	7	7	0	20872.436	-0.5
9	7	2	8	6	3	21101.215	-6
9	7	3	8	6	2	21101.215	-6
10	6	4	9	5	5	21329.757	-1.5
10	6	5	9	5	4	21329.757	-1.2
9	8	1	8	7	2	22329.636	-17.2
9	8	2	8	7	1	22329.636	-17.2
10	7	3	9	6	4	22558.361	-16.6
10	7	4	9	6	3	22558.361	-16.6
11	6	5	10	5	6	22786.719	-5.7
11	6	6	10	5	5	22786.719	-4.9
9	9	0	8	8	1	23558.023	8.1
9	9	1	8	8	0	23558.023	8.1
10	8	2	9	7	3	23786.832	-3.9
10	8	3	9	7	2	23786.832	-3.9
11	7	4	10	6	5	24015.47	1
11	7	5	10	6	4	24015.47	1
10	9	2	9	8	1	25015.219	16.2
10	9	1	9	8	2	25015.219	16.2

Table S66. Assigned transitions for the parent species of *cis*MOM-3w$_b$.

J'	K_a'	K_c'	J''	K_a''	K_c''	ν/MHz	$\Delta\nu$/kHz
5	2	3	5	1	4	2010.435	1.8
1	1	1	0	0	0	2037.4226	-1.5
4	2	2	4	1	3	2052.5964	-6
6	2	4	6	1	5	2056.6873	3.5
7	2	5	7	1	6	2216.275	2.4
5	2	4	5	1	5	3320.8299	-4.5
7	3	4	7	2	5	3397.1594	7.3
6	3	3	6	2	4	3601.8105	-1.4
3	1	3	2	1	2	3679.1689	-1.1
5	3	2	5	2	3	3782.7397	2.2
4	3	1	4	2	2	3914.4638	2.8
3	1	2	2	1	1	4021.1435	3.8
4	3	2	4	2	3	4081.6009	0.1
5	3	3	5	2	4	4142.1321	3.3
6	3	4	6	2	5	4242.3061	1.5
3	1	3	2	0	2	4328.4845	1.6
7	3	5	7	2	6	4391.5559	1.7
4	0	4	3	1	3	4512.1592	2.4
4	1	4	3	1	3	4893.2015	0.8
4	0	4	3	0	3	5030.2894	-5
2	2	0	1	1	1	5066.9247	9.4
4	2	3	3	2	2	5134.2261	-0.5
4	2	2	3	2	1	5247.4969	3.8
4	1	3	3	1	2	5345.4485	2.4
4	1	4	3	0	3	5411.3403	2
7	4	3	7	3	4	5480.5082	-0.6
6	4	2	6	3	3	5565.3096	-2.9
8	2	7	7	3	4	5597.4963	-10.1
5	4	1	5	3	2	5611.9875	-6.1
6	4	3	6	3	4	5627.9196	0.4
7	4	4	7	3	5	5628.9402	1.5
5	4	2	5	3	3	5633.6034	-8.5
4	4	0	4	3	1	5634.9563	-5
8	4	5	8	3	6	5644.7548	-9.5
9	4	6	9	3	7	5685.4125	8
5	0	5	4	1	4	5839.3665	0.6
3	2	2	2	1	1	6112.2576	9.3
5	0	5	4	0	4	6220.4114	1.6
5	2	4	4	2	3	6402.7146	0.6
5	3	3	4	3	2	6463.2407	-1.2
5	3	2	4	3	1	6479.6783	-3.6
5	1	5	4	0	4	6479.8028	-2.3
3	2	1	2	1	2	6514.4772	4.5
5	2	3	4	2	2	6611.4065	0.9
5	1	4	4	1	3	6653.5754	0.6
10	5	5	10	4	6	7047.0626	-6.5
6	0	6	5	1	5	7130.6483	2.2
9	5	4	9	4	5	7132.8117	-4.3
9	5	5	9	4	6	7183.7092	-5.5
8	5	3	8	4	4	7186.8868	-4.2
8	5	4	8	4	5	7207.2143	-2.4
7	5	2	7	4	3	7220.1741	-2
4	2	3	3	1	2	7225.3416	6.4
7	5	3	7	4	4	7227.1321	-8
6	1	6	5	1	5	7295.9507	-11.2
6	0	6	5	0	5	7390.0411	-0.3
8	2	6	7	3	5	7464.5605	-13.4
6	1	6	5	0	5	7555.358	0.6
6	2	5	5	2	4	7661.4611	-1.6
6	4	3	5	4	2	7755.953	7.2
6	4	2	5	4	1	7757.5093	-2.7
6	3	4	5	3	3	7761.6396	1
6	3	3	5	3	2	7804.1946	1.4
7	1	6	6	2	5	7842.7236	-0.7
3	3	1	2	2	0	7895.9422	-2
3	3	0	2	2	1	7908.8114	2.5
6	1	5	5	1	4	7938.8662	-1.9
6	2	4	5	2	3	7985.1198	1
7	0	7	6	1	6	8385.6849	-5.6
7	1	7	6	0	6	8651.05	-5.4
4	3	2	3	2	1	9156.3843	3.8
10	4	6	9	3	7	18790.038	6.7
9	5	5	8	4	4	18838.249	1
9	5	4	8	4	5	18861.333	8.4
11	4	8	10	3	7	19062.035	8.9

7	7	0	6	6	1	19513.793	4.6
7	7	1	6	6	0	19513.793	4.7
10	5	6	9	4	5	20092.372	9.7
9	6	4	8	5	3	20475.931	-13.8
9	6	3	8	5	4	20476.609	1.3
8	7	1	7	6	2	20803.498	-4.5
8	7	2	7	6	1	20803.498	-3.1
10	6	5	9	5	4	21759.113	-2.7
10	6	4	9	5	5	21761.421	-11
9	7	2	8	6	3	22092.55	-5.4
9	7	3	8	6	2	22092.55	4.3
8	8	0	7	7	1	22415.981	11.8
8	8	1	7	7	0	22415.981	11.8
10	7	3	9	6	4	23380.435	-4.6
9	8	1	8	7	2	23705.775	1.3
9	8	2	8	7	1	23705.775	1.4
10	8	2	9	7	3	24995.093	1.6
10	8	3	9	7	2	24995.093	2.1

Table S67. Assigned transitions for the parent species of *cis*MOM-4w$_a$.

J'	K_a'	K_c'	J''	K_a''	K_c''	ν/MHz	$\Delta\nu$/kHz
8	3	6	8	2	7	2042.9421	-4.4
3	1	3	2	2	1	2120.002	-6
2	2	0	1	1	1	3411.2023	-6.2
3	1	3	2	0	2	3640.3368	-6.1
4	0	4	3	1	3	4197.0035	4.2
4	1	4	3	1	3	4418.484	8.2
4	0	4	3	0	3	4472.8926	-2.3
4	2	3	3	2	2	4499.3065	5.5
4	1	3	3	1	2	4572.8139	0.1
3	2	1	2	1	2	4587.192	10.2
4	1	4	3	0	3	4694.3726	1.2
5	1	4	4	2	3	4759.3753	-3.7
6	2	5	5	3	2	4813.3386	-2
4	1	3	3	0	3	5081.3613	0.4
3	3	1	2	2	0	5271.7276	-1.8
3	3	0	2	2	0	5271.8612	-10.8
3	3	1	2	2	1	5274.6998	-0.2
3	3	0	2	2	1	5274.8443	1.6
5	0	5	4	1	4	5350.723	-0.2
5	1	5	4	1	4	5518.4075	0.4
4	2	3	3	1	2	5522.8621	-2
5	0	5	4	0	4	5572.1999	0.1
5	2	4	4	2	3	5620.3963	0.3
5	3	3	4	3	2	5635.8066	2.3
5	3	2	4	3	1	5638.7623	-1.6
5	1	4	4	1	3	5709.4311	1.6
5	1	5	4	0	4	5739.8865	2.9
4	2	2	3	1	3	5798.9577	-0.9
6	1	5	5	2	4	5979.8646	3
5	1	4	4	0	4	6317.9	4.6
4	3	2	3	2	1	6390.9018	-1.2
4	3	1	3	2	1	6391.9008	2.5
4	3	2	3	2	2	6405.6412	-0.3
4	3	1	3	2	2	6406.6381	1.2
6	0	6	5	1	5	6495.6687	-0.2
5	2	4	4	1	3	6570.4431	-3.1
6	1	6	5	1	5	6615.8871	2.7
6	0	6	5	0	5	6663.3537	0.9
5	2	3	4	1	3	6668.6265	0
6	2	5	5	2	4	6739.0442	-0.4
6	5	1	5	5	0	6759.8874	0.7
6	5	2	5	5	1	6759.8874	2.8
6	4	3	5	4	2	6762.4413	3
6	4	2	5	4	1	6762.6406	-0.6
6	3	4	5	3	3	6765.0074	1.1
6	3	3	5	3	2	6772.7813	1.2
6	1	6	5	0	5	6783.5694	1.2
6	2	4	5	2	3	6827.8328	0.9
6	1	5	5	1	4	6840.8792	0.8
7	1	6	6	2	4	7019.3174	-2.3
5	2	3	4	1	4	7055.6103	-5.7
4	4	1	3	3	0	7157.3319	-3.1
4	4	0	3	3	1	7157.4879	4.4
7	1	6	6	2	5	7206.2891	1.8

5	3	3	4	2	2	7498.7021	0.3
5	3	2	4	2	2	7502.6574	0.6
5	3	3	4	2	3	7542.1441	-0.7
5	3	2	4	2	3	7546.0955	-4.3
6	2	5	5	1	4	7600.0631	1.6
7	0	7	6	1	6	7628.7639	0.8
7	1	7	6	1	6	7710.975	-1.2
7	0	7	6	0	6	7748.9805	2
6	2	4	5	1	4	7787.0296	0.6
7	1	7	6	0	6	7831.195	3.3
7	2	6	6	2	5	7854.83	0.4
7	6	1	6	6	0	7886.1799	-5.8
7	6	2	6	6	1	7886.1799	-5.7
7	5	3	6	5	2	7888.36	-2.1
7	5	2	6	5	1	7888.3698	-3.5
7	4	4	6	4	3	7892.2792	-3.6
7	4	3	6	4	2	7892.9521	-2.3
7	3	5	6	3	4	7894.3191	-5.1
7	3	4	6	3	3	7911.374	2.6
7	1	6	6	1	5	7965.4752	4.8
9	2	8	8	3	5	7972.092	-1.1
7	2	5	6	2	4	7982.8632	-0.6
6	3	4	5	2	3	8588.5775	2.5
7	2	6	6	1	5	8614.0119	-0.7
8	0	8	7	1	7	8749.8107	1.3
8	1	8	7	0	7	8886.125	-5.3
11	8	3	10	7	4	18071.738	0
11	8	4	10	7	3	18071.738	0.1
12	7	6	11	6	5	18437.836	-7.4
12	7	5	11	6	6	18437.885	-3
10	10	0	9	9	1	18460.565	1.9
10	10	1	9	9	0	18460.565	1.9
11	9	2	10	8	3	18829.546	5.9
11	9	3	10	8	2	18829.546	5.9
12	8	4	11	7	5	19197.424	3.7
12	8	5	11	7	4	19197.424	4.4
11	10	1	10	9	2	19586.947	0.2
11	10	2	10	9	1	19586.947	0.2
13	8	5	12	7	6	20322.656	0.6
13	8	6	12	7	5	20322.656	3.3
11	11	1	10	10	0	20344.117	-7.1
11	11	0	10	10	1	20344.117	-7.1
12	10	2	11	9	3	20713.241	-1.6
12	10	3	11	9	2	20713.241	-1.6
14	8	6	13	7	7	21447.3	-5
14	8	7	13	7	6	21447.3	3.9
12	11	1	11	10	2	21470.519	1
12	11	2	11	10	1	21470.519	1
13	10	3	12	9	4	21839.4	1.7
13	10	4	12	9	3	21839.4	1.7
14	9	5	13	8	6	22206.998	4.7
14	9	6	13	8	5	22206.998	4.8
12	12	1	11	11	0	22227.584	10.9
12	12	0	11	11	1	22227.584	10.9
13	11	2	12	10	3	22596.834	-0.6
13	11	3	12	10	2	22596.834	-0.6
15	9	6	14	8	7	23332.064	-1.1
15	9	7	14	8	6	23332.064	-0.5
13	12	1	12	11	2	23353.972	-4.1
13	12	2	12	11	1	23353.972	-4.1
13	13	0	12	12	1	24110.894	-5.1
13	13	1	12	12	0	24110.894	-5.1

Table S68. Assigned transitions for the parent species of *cis*MOM-5w$_a$.

J'	K_a'	K_c'	J''	K_a''	K_c''	ν/MHz	$\Delta\nu$/kHz
3	1	3	2	1	2	2339.467	-7.7
3	1	2	2	1	1	2403.4739	-1.4
4	2	2	3	2	1	3168.0493	-6.9
4	1	3	3	1	2	3203.8957	5.2
5	0	5	4	0	4	3941.0458	2
5	2	3	4	2	2	3963.7547	0.6
5	1	4	4	1	3	4003.6375	2.1
6	1	6	5	1	5	4675.0914	-3.2
6	0	6	5	0	5	4722.8884	-3.4
6	2	5	5	2	4	4740.936	-4.9

6	2	4	5	2	3	4761.7161	-0.6
6	1	5	5	1	4	4802.5	-0.7
7	1	7	6	1	6	5452.2704	3.1
7	0	7	6	0	6	5501.6866	-1.4
7	2	6	6	2	5	5529.5438	0.5
7	5	2	6	5	1	5536.4713	3.6
7	5	3	6	5	2	5536.4713	3.8
7	3	5	6	3	4	5538.675	-2.4
7	3	4	6	3	3	5540.0372	0.5
7	2	5	6	2	4	5562.1008	-0.2
7	1	6	6	1	5	5600.2477	-1.9
8	1	8	7	1	7	6228.6529	4
8	0	8	7	0	7	6277.4093	0.1
8	2	7	7	2	6	6317.4312	-3.2
8	6	2	7	6	1	6327.215	3.2
8	6	3	7	6	2	6327.215	3.2
8	5	3	7	5	2	6327.8711	-1
8	5	4	7	5	3	6327.8711	-0.6
8	3	6	7	3	5	6330.8576	-0.3
8	3	5	7	3	4	6333.5625	0.2
8	2	6	7	2	5	6364.8197	0
8	1	7	7	1	6	6396.6142	1.6
9	1	9	8	1	8	7004.2207	6
9	0	9	8	0	8	7050.3125	11
9	2	8	8	2	7	7104.525	4.6
9	5	4	8	5	3	7119.4653	-4.2
9	5	5	8	5	4	7119.4653	-2.7
9	3	7	8	3	6	7123.225	-0.1
9	3	6	8	3	5	7128.15	4.8
9	2	7	8	2	6	7169.5125	1.6
9	1	8	8	1	7	7191.2853	0
10	1	10	9	1	9	7778.9615	-4.6
10	0	10	9	0	9	7820.8375	-7.1
10	2	9	9	2	8	7890.71	-2
10	5	5	9	5	4	7911.2817	-2.9
10	5	6	9	5	5	7911.2817	1.3
10	4	7	9	4	6	7913.6784	0.4
10	4	6	9	4	5	7913.9475	-3.7
10	3	8	9	3	7	7915.6994	0.1
10	3	7	9	3	6	7924.0424	-0.1
10	2	8	9	2	7	7975.5718	0.3
10	1	9	9	1	8	7983.9302	-0.6

Table S69. Assigned transitions for the parent species of *cis*MOM-6w$_a$.

J'	*K$_a$'*	*K$_{c'}$*	*J''*	*K$_a$''*	*K$_c$''*	*v*/MHz	*Δv*/kHz
4	1	4	3	1	3	2936.5724	-6.6
4	0	4	3	0	3	2964.0678	-2.8
4	2	3	3	2	2	2970.1041	-1.4
4	1	3	3	1	2	3002.0154	3.9
5	1	5	4	1	4	3669.5814	-4.7
5	0	5	4	0	4	3700.356	0.7
5	2	4	4	2	3	3711.7967	-0.7
5	3	3	4	3	2	3715.3742	4.4
5	3	2	4	3	1	3715.7182	-0.8
5	2	3	4	2	2	3724.7046	1.5
6	1	6	5	1	5	4401.9162	-4.5
6	0	6	5	0	5	4433.8679	0.2
6	2	5	5	2	4	4452.9375	1.8
6	3	3	5	3	2	4460.0179	-2.9
6	2	4	5	2	3	4474.9617	3.2
4	3	2	3	2	1	4490.4485	0
4	3	1	3	2	1	4490.5621	-3.1
4	3	2	3	2	2	4493.7448	-2.5
4	3	1	3	2	2	4493.8719	7.8
4	4	1	3	3	0	5100.7375	8.3
4	4	0	3	3	1	5100.7384	-7.7
7	1	7	6	1	6	5133.527	-0.7
7	0	7	6	0	6	5164.5936	-1.5
7	2	6	6	2	5	5193.4119	-2.1
7	5	2	6	5	1	5200.8958	-1.1
7	5	3	6	5	2	5200.8958	-0.8
7	3	5	6	3	4	5203.036	0.8
7	3	4	6	3	3	5205.1084	0.1
7	2	5	6	2	4	5227.3113	-0.9
5	3	3	4	2	2	5229.1608	-4.6

5	3	2	4	2	2	5229.6293	-2.2
5	3	3	4	2	3	5239.0096	-1.9
5	3	2	4	2	3	5239.479	1.4
6	2	5	5	1	4	5245.8276	7
7	1	6	6	1	5	5246.1218	0.2
7	1	7	6	0	6	5301.3593	5.2
8	1	8	7	1	7	5864.3873	0
8	0	8	7	0	7	5892.8683	2.6
8	2	7	7	2	6	5933.1262	-5.3
8	6	3	7	6	2	5943.6833	7.4
8	6	2	7	6	1	5943.6833	7.4
8	5	4	7	5	3	5944.409	-0.8
8	5	3	7	5	2	5944.409	-2.2
8	4	5	7	4	4	5945.7704	3.7
8	4	4	7	4	3	5945.8802	-0.9
8	3	6	7	3	5	5947.1281	-2.9
8	3	5	7	3	4	5951.2326	-0.8
6	3	4	5	2	3	5963.5543	-1.3
8	2	6	7	2	5	5981.3112	1
6	3	3	5	2	4	5987.7029	1.9
8	1	7	7	1	6	5991.322	-2.1
8	1	8	7	0	7	6001.1418	-4.4
5	5	1	4	4	0	6451.9471	-0.7
5	5	0	4	4	1	6451.9471	-1.1
5	5	1	4	4	1	6451.9471	-1.1
5	5	0	4	4	0	6451.9471	-0.8
9	1	9	8	1	8	6594.514	1.8
9	0	9	8	0	8	6619.2751	4.3
9	2	8	8	2	7	6671.9941	-1
9	7	3	8	7	2	6686.4658	-6.7
9	7	2	8	7	1	6686.4658	-6.7
9	6	3	8	6	2	6687.0899	2.1
9	6	4	8	6	3	6687.0899	2.2
9	5	5	8	5	4	6688.1361	1.9
9	5	4	8	5	3	6688.1361	-2.7
9	4	6	8	4	5	6690.0228	2.6
9	4	5	8	4	4	6690.2954	1.9
9	3	7	8	3	6	6691.2886	5.1
9	3	6	8	3	5	6698.6902	3.2
9	1	9	8	0	8	6702.7927	0
9	1	8	8	1	7	6734.5141	-0.8
9	2	7	8	2	6	6736.226	2.5
7	3	5	6	2	5	6736.4056	-1.2
7	3	4	6	2	5	6739.875	1.4
8	2	6	7	1	6	6753.8053	-6.6
6	5	2	5	4	2	7194.7754	-2.2
6	5	1	5	4	1	7194.7754	0.7
6	5	1	5	4	2	7194.7754	-2.3
6	5	2	5	4	1	7194.7754	0.8
10	1	10	9	1	9	7323.9485	5
7	4	4	6	3	3	7327.706	-1.7
7	4	3	6	3	3	7327.764	-1.1
7	4	4	6	3	4	7329.1	-1.4
7	4	3	6	3	4	7329.1625	3.6
10	0	10	9	0	9	7344.4978	0.4
10	2	9	9	2	8	7409.9203	-2.1
8	3	6	7	2	5	7411.4534	2.2
10	8	3	9	8	2	7429.275	-0.4
10	8	2	9	8	1	7429.275	-0.4
10	7	3	9	7	2	7429.8107	-2.6
10	7	4	9	7	3	7429.8107	-2.6
10	6	5	9	6	4	7430.6583	-1.1
10	6	4	9	6	3	7430.6583	-1.2
10	5	6	9	5	5	7432.0968	1.4
10	5	5	9	5	4	7432.11	1.5
10	4	7	9	4	6	7434.598	4.2
10	4	6	9	4	5	7435.183	0.8
10	3	8	9	3	7	7435.364	-2.8
10	3	7	9	3	6	7447.7747	-0.9
10	1	9	9	1	8	7475.2959	-3.5
6	6	0	5	5	1	7803.1364	-0.8
6	6	1	5	5	0	7803.1364	-0.8
6	6	1	5	5	1	7803.1364	-0.8
6	6	0	5	5	0	7803.1364	-0.8
7	5	3	6	4	2	7937.5076	3.7
7	5	2	6	4	2	7937.5076	3.2
7	5	2	6	4	3	7937.52	0

7	5	3	6	4	3	7937.52	0.4
11	0	11	10	0	10	8069.15	-5.6
